# Bolapolyphiles With Bulky Carbosilane‐Based Lateral Groups—Reticular Design of Liquid Crystalline Pentagonal Honeycombs

**DOI:** 10.1002/chem.71595

**Published:** 2026-08-19

**Authors:** Christian Anders, Matthias Wagner, Silvio Poppe, Carsten Tschierske

**Affiliations:** ^1^ Organic Chemistry Institute of Chemistry Martin Luther University Halle‐Wittenberg Halle (Saale) Germany

**Keywords:** carbosilanes, honeycombs, hydrogen bonding, liquid crystals, pentagon tiling, self‐assembly, structural complexity

## Abstract

Periodic pentagon tessellations are rarely observed in self‐assembled molecular systems. Here, it is reported that *p‐*terphenyl‐based bolapolyphiles with sticky glycerol groups at both ends and highly branched carbosilane‐based side‐groups self‐assemble into liquid crystalline honeycombs composed of type‐2 pentagonal prismatic cells and *p*2*gg* plane group symmetry over wide temperature and side‐group volume ranges. It is shown that bulky side‐groups with two cycloalkyl (cyclobutyl, cyclohexyl) or branched alkyl chains (*iso*‐pentyl, *iso*‐hexyl, and *tert*‐hexyl) at the silicon branching point favor the formation of pentagon tilings, while three of them reduce the pentagonal honeycomb range, mainly by favoring crystalline phases.

## Graphical abstract

T‐shaped bolapolyphiles with highly branched carbosilane‐based side‐groups self‐assemble into liquid crystalline honeycombs composed of type‐2 pentagonal prismatic cells and *p*2*gg* plane group symmetry.

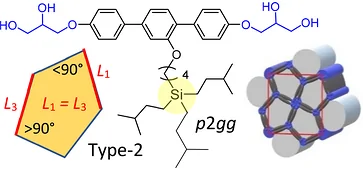

## 1 Introduction

The regular pentagon with five identical sides and angles is well known for its golden ratio and as a building block of the dodecahedron, having 12 pentagonal faces. In contrast, the Euclidian plane cannot be decorated with regular pentagons without overlappings or gaps, and therefore a regular pentagon tiling with fivefold symmetry is impossible. Tessellation with pentagons is only possible after deformation to non‐regular pentagons. There are in total 15 different types of tiling of the Euclidian plane by identical non‐regular convex pentagons (Figure 1) [1–3], the last one has only recently been discovered [4]. It was also proven that this series is now complete and no additional tilings could be expected to be found in the future [5].

**FIGURE 1:**
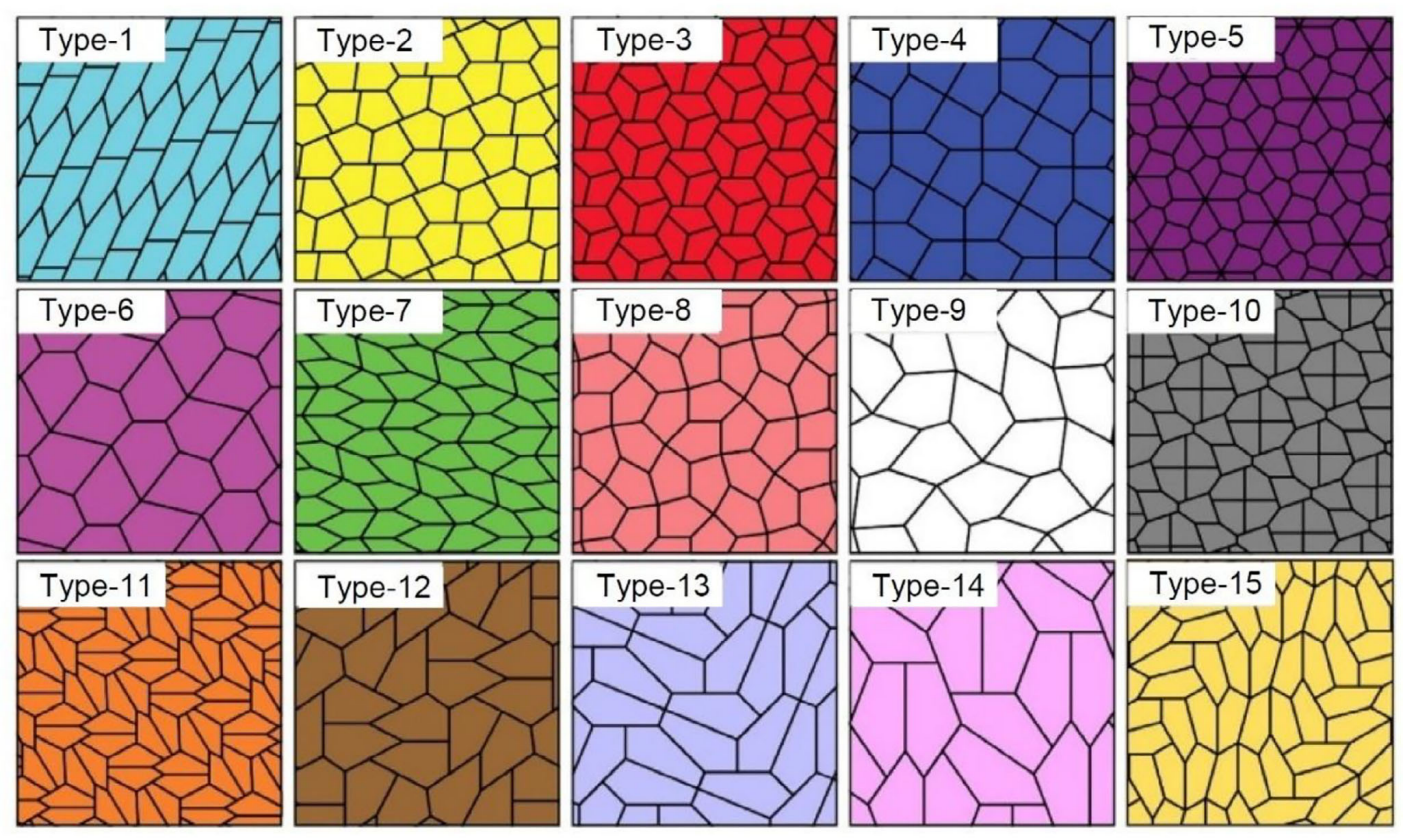
The 15 types of monohedral tiling patterns with non‐regular convex pentagons; note that the type of tiling is related to the type of pentagon involved and does not distinguish between edge‐to‐edge (4‐9) and non‐edge‐to‐edge tilings (1‐3, 10‐15). Type‐1 and type‐2 tilings can form both types, though only the non‐edge‐to‐edge versions are shown here; but only the edge‐to‐edge tilings (see for example Figure 2c,d) can be expected for self‐assembled molecular systems. Adapted from ref [6].

Due to the unique features of the pentagon tiling, there have been numerous attempts to produce them in chemical systems with pentagonal molecules and coordination complexes in bulk or on surfaces [7, 8, 9, 10, 11, 12, 13, 14, 15, 16, 17, 18, 19]. However, either a loose, distorted packing is observed, or rotational disorder leads to the packing on a hexagonal lattice. Also, attempts to transfer fivefold molecular symmetry to liquid crystal self‐assembly have failed with pentagonal molecules [10, 20]. Pentagonal nets [21] based on covalent inorganic 2D materials, such as pentagraphene [22] penta‐PdSe_2_, [23, 24] penta‐PdS_2_ [25] and penta‐NiN_2_ [26] have recently been reported and reviewed [27]. It turned out that most of these monohedral edge‐to‐edge tilings by identical convex pentagons can be traced back to the type‐2 tiling, having plane group *p*2*gg*, and the type‐4 tiling, also known as Cairo‐tiling, with *p*4*gm* plane group (Figure 2d, e). Only one other uniform tiling, the type‐1 tiling, is presently known (penta‐SrP_2_) [28]. In the type‐4 tiling, four pentagons join at 4‐way nodes with 90° angles, while in the type‐2 pentagons the angles are unequal 90° (Figure 2d). Thus, in the type‐4 pentagon the angles α_1_ and α_3_ are 90 ° while in the Type‐2 pentagon α_1_ and α_3_ are different and together give 180 ° (Figure 2e) [2, 3]. Notably, both tiling patterns combine 3‐way and 4‐way junctions in the ratio 2:1, and this requires either a combination of planar trigonal with planar tetragonal junctions or tetrahedral junctions in different orientations with respect to the tiling plane (Figure 2 g, f) [21]. For atomic and molecular pentagonal nets, the angles are limited by the bond angles or coordination geometries and require a buckling in the case of tetrahedral junctions [21, 27]. This problem also arises in attempts to organize properly designed molecules into one of the 15 pentagon tiling motifs using self‐assembly [29], where the geometry of the intermolecular interactions between the available building blocks (for example, by hydrogen bonding between carboxylic acids, amides, or *N*‐heterocycles) and the fixed coordination geometries of metals in coordination complexes limits the possible angles. Therefore, only very few organic/inorganic hybrids and purely organic materials with pentagon tilings have been reported so far, among them very few metal‐organic [21, 30] and covalent organic frameworks with **mcm** topology and pentagonal pores [31, 32, 33].

**FIGURE 2:**
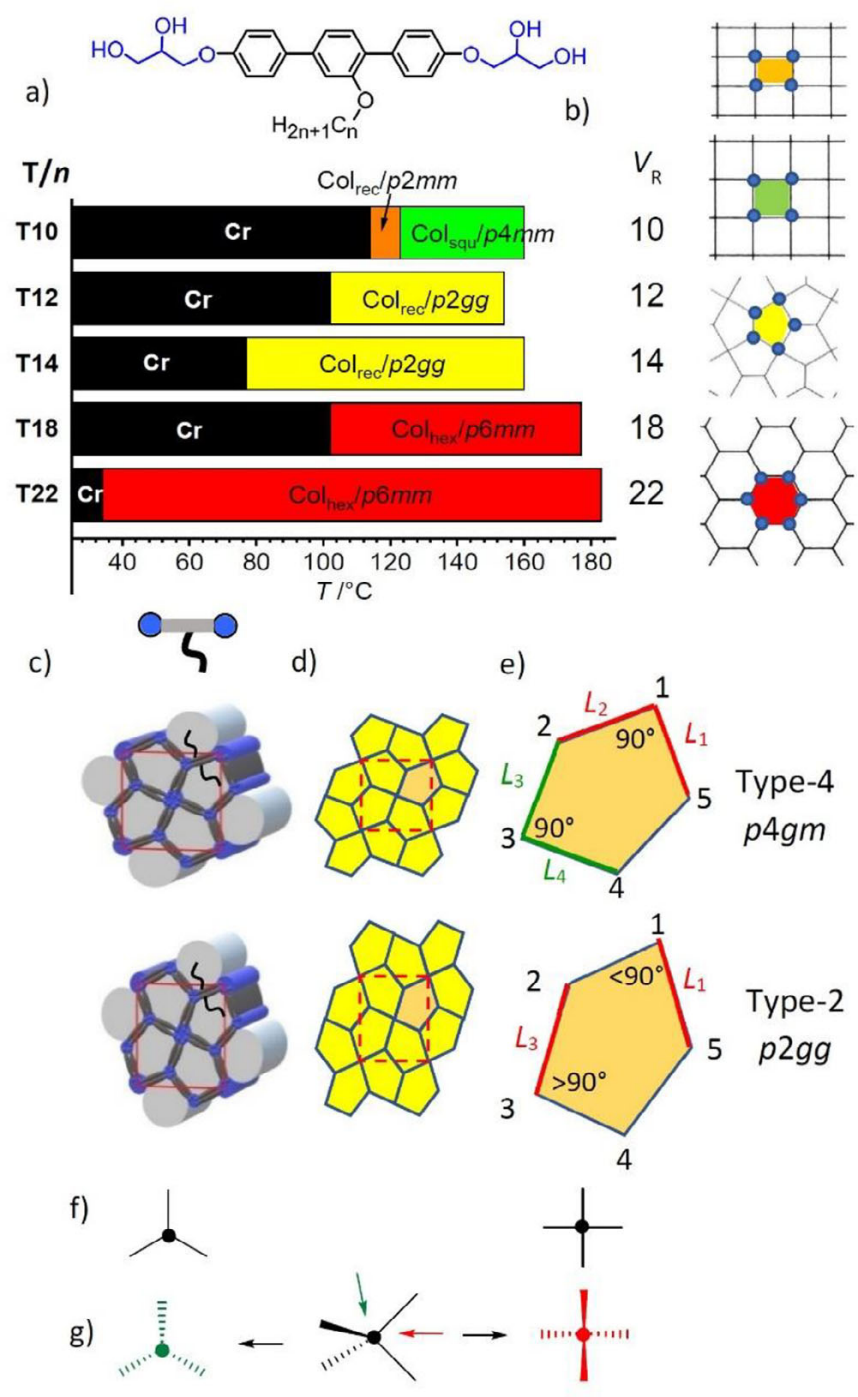
LC honeycombs and their tiling patterns. a) Molecular structure and bar diagram showing the phase transitions of the T‐shaped bolapolyphiles **T*n*** depending on side‐chain length *n* (data on heating) [55] with b) the tiling patterns of their LC honeycomb phases; *V*_R_ is a measure of side‐chain volume and gives the total number of C atoms in the side‐chain; c) shows the schematic sketch of a T‐shaped bolapolyphile and its self‐assembly into type‐4 or type‐2 pentagonal honeycombs (blue = polar glycerol columns, dark gray = rod‐like units and light gray shows the space in the pentagonal prismatic cells filled by the side‐chains); d) shows the corresponding tiling patterns (cuts perpendicular to the honeycomb long axis), and e) shows the involved pentagons, for the type‐4 pentagon the angles α_1_ = α_3_ = 180° and the side‐lengths *L*_1_ = *L*_2_ and *L*_3_ = *L*_4_; note that the numbering chosen for the type‐4 pentagon is retained in the type‐2 pentagon, thus α_1_+ α_3_ = 180°, α_2_ + α_4_ + α_5_ = 360° and *L*_1_ = *L*_3_; f) shows trigonal 3‐way and tetragonal 4‐way junctions as required for the pentagon tessellations, while g) shows that tetrahedral junctions can provide either triangular or square junctions by buckling the pentagon tiling.

If, however, a dynamic and flexible mode of non‐covalent interaction between the molecules is chosen, then almost any required angle can develop during self‐assembly. Such dynamic and flexible intermolecular interactions are provided by the hydrogen bonding in alcohols, water and polyalcohols, like glycerol and carbohydrates in the liquid state. These hydrogen bondings are *highly dynamic* due to a rapid exchange of the protons between donor and acceptor OH‐groups [34], (Grotthuss mechanism) [35], and due to the easy rotation around the involved single bonds. Moreover, these H‐bondings are cooperative in nature, meaning that each H‐bond polarizes the involved atoms and thus strengthens the next developing H‐bonds, leading to large *polymeric networks*, dominating over dimers and small aggregates [34, 36, 37].

Here we use the glycerol unit as flexible and cohesive unit attached to both ends of rod‐like molecular units. Such rigid and lipophilic π‐conjugated rods with sticky polar glycerol groups at both ends represent a specific class of amphiphiles, the so‐called *bolaamphiles* [38, 39]. The rod‐like units of these molecules have a high tendency to aggregate by parallel alignment. Flexible chains were attached side‐on to the π‐conjugated rods in order to retain a liquid‐like state, and hence, the required dynamics. These flexible and lipophilic alkyl chains are incompatible with both the rigid π‐conjugated rods as well as with the polar glycerols, that is, these block‐molecules [43, 44, 48] are composed of three incompatible building blocks, and represent multiple amphiphiles, so‐called *polyphiles*, in the specific case considered here, *bolapolyphiles* [42, 48, 49, 50, 51]. By self‐assembly, these bolapolyphiles organize into periodic structures, while molecular rotations and long‐range translational motions retain the fluidity. These states of matter between the isotropic liquid and the solid‐state crystal represent mesophases combining mobility with long‐range order, also known as *liquid crystals* (LCs) [40, 41, 42, 43, 44, 45, 46, 47].

Bolapolyphiles represent a special kind of LCs, being capable of soft self‐assembly into complex structures where space is divided into three or even more distinct compartments [42, 43, 44]. The most common among them are LC honeycombs [48, 49, 50], though also numerous 3D‐network structures have been discovered [51]. In the honeycomb LCs, the hydrogen‐bonded glycerols at the ends of the rods form strings or columns. These columns fuse the linear polyaromatic rods not only end‐to‐end, but also side‐by‐side, thus forming ribbons, being interconnected by the polar glycerol columns to the polygonal honeycombs (Figure 2c). With a growing volume of the side‐chain, the polygonal cells can assume different sizes and shapes, ranging from triangular to hexagonal (Figure 2a, right) [42, 48, 49]. Besides the regular tilings by triangles, squares, and hexagons, also non‐regular tilings by pentagons were observed at the transition from square to hexagonal cells [52, 53, 54, 55]. It was found that facial polyphiphiles (having exchanged positions of polar and lipophilic groups compared to bolapolyphiles) [52, 56, 57], as well as bolapolyphiles involving relatively long oligothiophene based rod‐like cores [58, 59, 60, 61], preferably form the more symmetric type‐4 tiling with *p*4*gm* lattice, while bolapolyphiles with shorter biphenyl or *p*‐terphenyl cores prefer the type‐2 tiling with *p*2*gg* plane group [53, 54, 55]. As an example, Figure 2a, b shows the development of the distinct honeycombs and their tiling patterns for T‐shaped bolapolyphiles with a *p*‐terphenyl core and a single (even‐numbered) linear *n*‐alkyl chain at the central benzene ring [55]. In this series, the formation of honeycombs with type‐2 pentagonal cells (*p*2*gg*) is observed for chain lengths *n* = 12 and 14.

In order to study the effect of the side‐chain structure on pentagon formation in a systematic way, different chain architectures were studied [62, 63, 64, 65], including those with branched chains [66, 67, 68, 69, 70]. However, the access to highly branched alkyl chains is limited and often requires non‐convergent multistep synthesis. Therefore, we use carbosilanes as side‐groups, where the Si‐atoms are connected via an oxyalkylene spacer unit to the rod‐like core and act as branching points with tunable distance from the core unit. Here, cross‐coupling reactions between Si‐halides and organometal reagents and hydosilylation reactions provide an easy access to a wide range of different branching patterns. In previous work, the carbosilanes were mainly used as end‐groups [71, 72, 73, 74, 75, 76, 77, 78, 79, 80] and as oligomeric and dendritic scaffolds [81, 82, 83], but rarely as side‐groups [80, 84, 85, 86, 87, 88, 89].

To this end, *p*‐terphenyl‐based bolapolyphiles with branched carbosilane side‐groups (R) were synthesized and investigated (see Scheme 1) [89]. Focus herein is on side‐groups with a highly crowded trialkylsilyl end with two or three branched or cyclic aliphatic substituents (R^1^) attached to the Si branching point.

**SCHEME 1:**
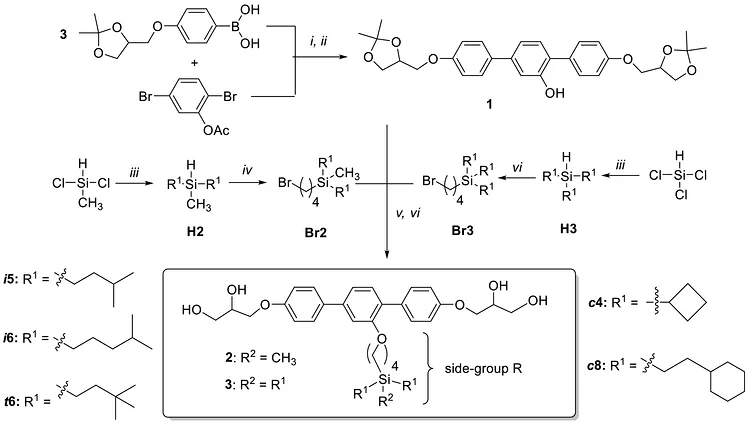
Synthesis of the bolapolyphiles **2** and **3**; conditions: *i*: Pd(PPh_3_)_4_, NaHCO_3_, H_2_O, THF, reflux; *ii*: NaOH, H_2_O, 80°C; *iii*: R‐MgBr, LiCl, THF, 0°C; *iv*: Br‐(CH_2_)_2_‐CH=CH_2_, Karstedt cat., Et_2_O, reflux; *v*: K_2_CO_3_, DMF NBu_4_I, 120°C; *vi*: PPTS, THF, MeOH, 50°C. In the compound abbreviations, the compound number indicates the number of branched/cyclic groups (R^1^) at the Si‐branching point, followed by an italic letter with *i* = iso, *t* = tert, and *c* = cyclic, while the last number indicates the total number of C atoms in each of the substituents R^1^.

It is shown that this kind of molecules can provide a wide range of type‐2 pentagonal honeycomb LCs and that side‐groups with two bulky branched or cyclic substituents R^1^ at the silicon favor the formation of pentagon tiling compared to linear chains, while three of them reduce the pentagonal honeycomb ranges, mainly by favoring crystalline phases.

## 2 Results and Discussion

### 2.1 Synthesis

The synthesis of compounds **2** and **3** is shown in Scheme 1 and starts with *p*‐terphenyl‐2’‐ol **1,** which was synthesized by Suzuki cross‐coupling of the 2,5‐dibromophenylacetate with two equivalents of 4‐[(2,2‐dimethyl‐1,3‐dioxolane‐4‐yl)methoxy]benzene boronic acid, followed by alkaline cleavage of the acetate protecting group as reported previously [55]. The bromo‐functionalized carbosilanes **Br2** and **Br3** were obtained from dichloromethylsilane or trichlorosilane, respectively, by cross‐coupling with two, respectively, three equivalents of the appropriate Grignard reagent in the presence of LiCl, [90] to give the silanes **H2** and **H3,** which were used for the hydrosilylation of 4‐bromobutene [91]. Alkylation of **1** with **Br2** or **Br3,** followed by acidolytic deprotection of the acetonide groups, yielded the bolapolyphiles **2** with two large alkyl substituents and one CH_3_ group at the Si‐branching point and compounds **3** having three identical branched/cyclic alkyl substituents.

### 2.2 Investigations

Investigations were conducted by polarizing optical microscopy (POM, Olympus BX51‐P polarizing microscope, with Mettler FP82‐HAT heating stage), differential scanning calorimetry (DSC; DSC‐8000, Perkin Elmer), and X‐ray scattering at Cu Kα line (*λ* = 1.54 Å) using a standard Coolidge tube source and Vantec 500 area detector. For more details, see Section S1 of the SI.

The transition temperatures and related transition enthalpy values of the LC phases and their lattice parameters are collated in Tables 1 and 3, while Table 2 summarizes the structural data of the mesophases of all compounds. For simplicity, herein we use the total number of carbon (C) and silicon atoms (Si) in these side‐groups as a measure of their volume (*V*_R_) and the number C+Si along the longest branch from the core to the most distant end of the side‐group as a measure of its length (*L*_R_). Note that, in contrast to linear side‐chains, for branched side‐chains *V*_R_ and *L*_R_ are decoupled, because chain branching reduces the chain length at constant chain volume. For completeness, the side‐group volumes calculated with volume increments are given in Table S8 and their volume fractions *ϕ*_R_ in Tables 1 and 3.

**TABLE 1:** Phase transitions, lattice parameters and other data of compounds **2**.^a^

Comp.	*V*_R_	*L*_R_	Phase transitions *T*/°C [Δ*H*/kJ∙mol^−1^]	*a*, *b*/nm (*T*/°C)	*ϕ*_R_
**2*c*4**	14	8	H: Cr_1_ 110 [9.4] Cr_2_ 129 [8.7] Col_rec_/*p*2*mm* ^*b*^ 136 [0.7] Col_squ_/*p*4*mm* 167 [6.8] Iso C: Iso 163 [‐6.8] Col_squ_/*p*4*mm* 132 [‐0.8] Col_rec_/*p*2*mm* ^*b*^ 122 [‐8.6] Cr	2.64 (140)	0.40
**2*i*5**	16	9	H: Cr_1_ 75 [9.1] Cr_2_ 96 [9.3] Col_rec_/*p*2*gg* 168 [7.6] Iso C: Iso 165 [‐7.7] Col_rec_/*p*2*gg* 66 [‐8.5] Cr	6.78, 6.36 (140)	0.45
**2*i*6**	18	10	H: Cr 74 [10.5] Col_rec_/*p*2*gg* 164 [6.7] Iso C: Iso 160 [‐6.8] Col_rec_/*p*2*gg* 69 [‐3.1] Cr	6.74, 6.32 (140)	0.48
**2*t*6**	18	9	H: Cr 106 [14.1] Col_rec_/*p*2*gg* 170 [6.9] Iso C: Iso 167 [‐7.0] Col_rec_/*p*2*gg* 87 [‐10.2] Cr	6.71, 6.37 (140)	0.48
**2*c*8**	22	11	H: Cr 99 [5.5] Col_rec_/*p*2*gg* 151 [5.5] Iso C: Iso 147 [‐5.6] Col_rec_/*p*2*gg* 91 [‐4.5] Cr	6.66, 6.40 (140)	0.52

^a^ Peak temperatures and transition enthalpies as determined by DSC at 10 K min^−1^ on second heating (H:) and cooling (C:); *V*_R_ = total number of carbon (C) and silicon atoms (Si) in the side‐groups; *L*_R_ = number of C+Si along the longest branch from the core to the most distant end of the side‐group; *ϕ*_R_ is the volume fraction of the side‐groups; the calculated volumes of the molecules and their distinct parts can be found in Table S8; Cr, Cr_1_… = crystalline solids which were not investigated, Iso = isotropic liquid, Col_squ_/*p*4*mm* = square LC honeycomb, Col_rec_/*p*2*mm* = LC honeycomb with rectangular cells; Col_rec_/*p*2*gg* = pentagonal LC honeycomb; for additional structural data, see Tables 2 and S8, for textures, see Figures 3c, 4c and S1, S2, and for DSC traces, see Figures 3a, 4a and S3.

^b^ Phase assignment based on POM investigations, see Figure S1a‐d.

**TABLE 2:** Structural parameters of the LC phases of compounds **2** and **3**.^a^

Comp.	Phase	*a*,*b*/nm	*V*_mol_/nm^3^	*V*_cell_/nm^3^	*n*_cell_	*n*_wall_
**2*c*4**	Col_squ_/*p*4*mm*	2.64	0.86	3.21	3.3	1.7
**2*i*5**	Col_rec_/*p*2*gg*	6.78, 6.36	0.94	19.84	18.8	1.9
**2*i*6**	Col_rec_/*p*2*gg*	6.74, 6.32	0.99	19.59	17.7	1.8
**2*t*6**	Col_rec_/*p*2*gg*	6.71, 6.37	0.99	19.66	17.7	1.8
**2*c*8**	Col_rec_/*p*2*gg*	6.66, 6.40	1.06	19.61	16.5	1.7
**3*c*4**	Col_squ_/*p*4*mm*	2.59	0.92	3.09	3.0	1.5
**3*i*5**	Col_rec_/*p*2*gg*	6.55. 6.42	1.04	19.34	16.6	1.7

^a^ *V*_cell_ = unit cell volume, calculated according to *V*_cell_ = $\;a_{\mathrm{squ}}^{2}$∙*h* for Col_squ_, *V*_cell_ = *a∙b*∙*h* for Col_rec_ (*h* = 0.46 nm; this value is an average value for LC phases of compounds with carbosilane chains and used for all compounds **2** and **3**); *V*_mol_ = molecular volume, calculated with the crystal volume increments of Immirzi and Perini; [92] *n*_cell_ = number of molecules per unit cell, calculated according to *n*_cell_ = 0.893 ∙ (*V*_cell_/*V*_mol_), where 0.893 is the correction factor for the packing density in the LC state. (Origin of correction factor: The used crystal volume increments consider the packing coefficient of *k* = 0.7 in the solid state [93]. In the liquid state the packing coefficient is only *k* = 0.55 and therefore *n*_cell, liqu_ = (0.55/0.7)  = 0.786 *n*_cell, cr_ For the LC state an intermediate packing coefficient between the crystalline and the liquid state is assumed, corresponding to the average between them, calculated according to *n*_cell_ = (*n*_cell, cr_ + *n*_cell, liqu_)/2 = (*n*_cell, cr_ + 0.786 *n*_cell, cr_)/2, leading to the used correction factor 0.893); *n*_wall_ = number of molecules in the lateral cross‐section of the honeycomb walls, calculated according to *n*_wall_ =  *n*_cell_/2 for Col_squ_, and *n*_wall_ = *n*_cell_/10 for Col_rec_/*p*2*gg;* for additional structural data, see Table S8.

**TABLE 3:** Phase transitions, lattice parameters and other data of compounds **3**.^a^ ^.^

Comp.	V_R_	*L*_R_	Phase transitions *T*/°C [Δ*H*/kJ∙mol^−1^]	*a, b* /nm (*T*/°C)	*ϕ*_R_
**3*c*4**	17	8	H: Cr_1_ 171 [33.1] Iso C: Iso 159 Col_squ_/*p*4*mm* 157 [‐4.9]^b^ Col_rec_/*p*2*mm* 147 [‐16.9] Cr H^c^: Col_rec_/*p*2*mm* 160 [3.0] Col_squ_/*p*4*mm* 163 [2.0] Iso	2.59 (158)	0.44
**3*i*5**	20	9	H: Cr_1_ 98 [2.5] Cr_2_ 141 [34.6] Col_rec_/*p*2*gg* 152 [4.8] Iso C: Iso 148 [‐4.8] Col_rec_/*p*2*gg* 89 [‐4.3]^*d*^ Cr H^e^: Cr 104 [11.6] Col_rec_/*p*2*gg* 152 [5.0] Iso	6.55, 6.42(140)	0.51
**3*t*6**	23	9	H: Cr_1_ 138 [27.8] Cr_2_ 173 [11.1] Cr_3_ 184 [32.1] Iso C: Iso 157 [‐1.3] ^*d*^ Cr_2_ 111 [‐22.1] Cr_1_	—	0.54

^a^ Determined by DSC at 10 K min^−1^ on first heating (H:) and cooling (C:); peak temperatures are given for the LC phases, while for the crystalline phases the temperatures of the onset of crystallization and the end of melting are given; for abbreviations, see Table 1 and for additional structural data, see Tables 2 and S8, for DSC‐traces, see Figure S3d–f.

^b^ Enthalpies of both transitions together.

^c^ Second heating after cooling to 155°C to suppress crystallization.

^d^ Slow partial crystallization; that no transition to an LC phase takes place at this transition was confirmed by POM.

^e^ Second heating.

### 2.3 Compounds 2 With Two Cyclic or Branched Substituents at the Si Branching Point

#### 2.3.1 Square and Rectangular Honeycombs

All synthesized compounds of the series of compounds **2** show LC phases in broad temperature ranges (Table 1). Compound **2*c*4** with a 4‐(dicyclobutylmethylsilyl)butyl side‐group (Figure 3a), having the smallest side‐group volume of the investigated compounds, shows two LC phases between 129 and 167°C upon heating and between 163°C and 122°C upon cooling (Table 1, Figure 3a). In the high temperature range, between 136 and 167°C upon heating and between 163 and 132°C upon cooling, the typical fan‐like and spherulitic textures of a columnar LC phase are observed between crossed polarizers (Figure 3c). The areas between the birefringent fans, where the columns are aligned perpendicular to the substrate surfaces (homeotropic alignment), appear completely dark between crossed polarizers and remain dark if the sample is rotated between them. This indicates the optical uniaxiality of this LC phase, which thus could be either a hexagonal or a square phase.

**FIGURE 3:**
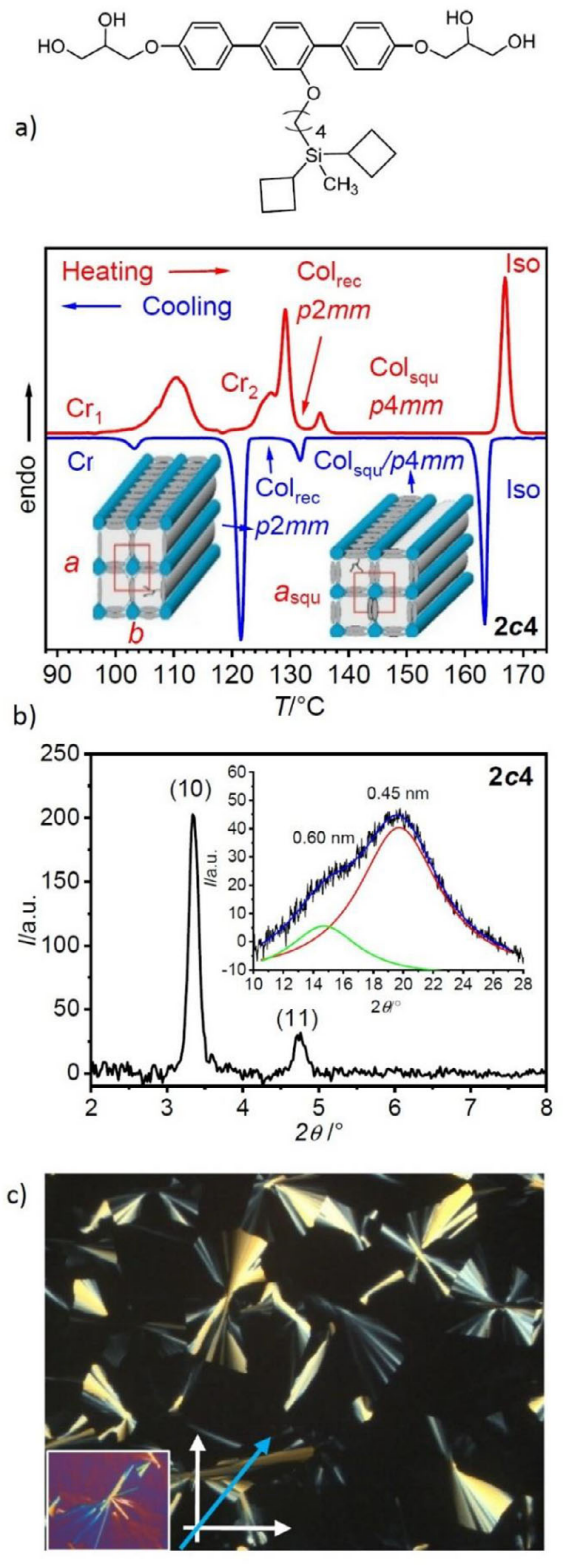
Investigation of compound **2*c*4**; a) molecular structure and second DSC heating and cooling curves at 10 K min^−1^, together with the models of the organization of the molecules in the honeycomb LCs with *p*2*mm* (left) and *p*4*mm* (right) plane groups; b) SAXS pattern of the Col_rec_/*p*4*mm* phase at 145°C with inset showing the WAXS at the same temperature, for numerical X‐ray data, see Table S1; c) POM texture as observed between crossed polarizers in the Col_squ_/*p*4*mm* phase at 145°C, the dark areas indicate optical uniaxiality of this LC phase and the inset shows the texture with λ−retarder plate; the blue shift in SW‐NE direction indicates negative birefringence, that is the major intramolecular π‐conjugation pathway of the terphenyls is perpendicular to the column long axis; white arrows indicate the directions of polarizer and analyzer, the blue arrow indicates the direction of the indicatrix slow axis, for textural changes at the Col_squ_/*p*4*mm—*Col_rec_/*p*2*mm* transition see Figure S1a‐d.

The small‐angle x‐ray scattering (SAXS) pattern shows two reflections with the ratio of 1/*d*‐values 1: √2 at 145°C, indicating a square lattice (Figure 3b). The WAXS scattering is diffuse (see inset in Figure 3b) with a maximum of the diffuse scattering at 0.45 nm, as typical for liquids and LC phases. There is an additional shoulder at 0.57 nm, which we attribute to the mean distance between the Si‐branching points. The lattice parameter of the square cell with *p*4*mm* plane group is *a*_squ_ = 2.64 nm (Table 1) and corresponds to the length of the bolaamphiphilic core of *L*_mol_ = 2.6 nm as measured for all compounds **2** with stretched conformation of both glycerol end groups (see Figure S6a). This means that the *p*‐terphenyl cores form a square honeycomb (see model in Figure 3a, right). In this honeycomb, the alignment of the *p*‐terphenyls is perpendicular to the *c*‐direction of the honeycomb. This mode of alignment is confirmed by the negative birefringence, indicated by POM investigation with an additional λ‐retarder plate (see inset in Figure 3c). The slow optical axis of the retarder plate (blue arrow) is oriented southeast‐northwest (SE‐NW). The alignment of the honeycombs in the birefringent fans is with their *c*‐axis parallel to the substrate surfaces, fusing end‐to‐end to cycles in order to avoid energetically unfavorable interfaces at the ends of the honeycombs, thus giving rise to the typical spherulitic and fan‐like textures. A blue shift of the fans is observed along the indicatrix slow axis (SE‐NW), indicating that the major intramolecular π‐conjugation pathway is in this direction. As the local direction of the honeycomb *c*‐axis is perpendicular to the slow indicatrix axis, it follows that the *p*‐terphenyls are arranged perpendicular to the honeycomb *c*‐axis as required for a honeycomb LC.

The number of molecules per unit cell with an assumed height of *h* = 0.46 nm (average of the maximum of the diffuse WAXS scattering in the LC phases of compounds with carbosilane chains) is *n*_cell_ = 3.4 (*n*_cell_ = 0.893 *V*_cell_/*V*_mol_ with *V*_mol_ calculated with Immirzi's crystal volume increments [92] and 0.893 as a factor considering the reduced packing density in the LC state, (see footnote of Table 2 for details). As there are two molecules per unit cell, about two molecules are arranged in the lateral cross section of the square cylinder walls (*n*_wall_ = *n*_cell_ = *n*_wall_/2 = 1.7, see Table 2), in line with a back‐to‐back arrangement of the terphenyls in the honeycomb walls with their side‐groups filling the resulting square cells (see model in Figure 3a, right). Because LC phases represent highly dynamic systems where individual molecules do not assume fixed positions, *n*_wall_ is space‐and time‐averaged and not necessarily a full number. That *n*_wall_ is a bit smaller than exactly 2.0 is due to some staggering of the molecules along the honeycomb walls (see Figure S6b).

At the phase transitions at 129°C on cooling, a typical stripe pattern appears in the dark homeotropic areas of the Col_squ_/*p*4*mm* phase, indicating the transition to a biaxial phase (Figure S1a‐d). This optical texture and the small transition enthalpy value of 0.8 kJ mol^−1^ (Figure 3a and Table 1) are typical for the transition from Col_squ_/*p*4*mm* to Col_rec_/*p*2*mm* due to the deformation of the square to rectangular cells by stretching two opposite sides (see model in Figure 3a, left). This transition is typically observed for square honeycombs by reducing temperature, as previously found for the T‐shaped compound **T10** (Figure 2a) [55]. However, due to the onset of crystallization during XRD exposure time, the lattice parameters of the *p*2*mm* phase of **2*c*4** could not be determined in this case.

#### 2.3.2 Pentagonal Honeycombs

All the following compounds **2** with two branched or cyclic alkyl groups at Si have a larger side‐group volume *V*_R_ and show a different type of honeycomb phase (Table 1).

The data of compound **2*i*5** with two isopentyl groups at the silicon are shown as a representative example in Figure 4. In this case a LC phase is observed between 96 and 168°C on heating and between 165 and 66°C on cooling (Table 1 and Figure 4a). The POM texture between crossed polarizers is again typical for a columnar phase, and it shows negative birefringence, in line with a honeycomb structure (Figure 4c). However, the areas between the birefringent fan‐like domains show a weakly birefringent stripe pattern, which is a first hint on the optical biaxiality of this LC phase. The SAXS pattern can be indexed to the (11), (20), (02) (21), and (12) reflections of a rectangular lattice with *p*2*gg* plane group, and the parameters *a*_rec_ = 6.78 nm and *b*_rec_ = 6.36 nm at 140°C (see Table 1 and Figure 4b). The WAXS is diffuse as typical for LC phases (inset in Figure 4b). In this case, there is no visible shoulder at larger distances, probably as the contribution of the hydrocarbon groups becomes dominating in determining the average lateral distances. The shift of the scattering maximum from the usually observed value of 0.45 nm to 0.47 nm might be due to the contribution of the larger distance provided by silicon. The lattice parameters of the *p*2*gg* lattice are larger than twice the single molecular length (*L*_mol_ = 2.6 nm), corresponding to 2.4‐2.6 times of its length. This combination of optical properties, plane group symmetry, and lattice parameters are characteristic for a pentagonal honeycomb LC where the unit‐cells involve four identical nonregular pentagonal cells tiling the Euclidian plane without gaps and overlaps (inset model in Figure 4b). The dual of the pentagonal honeycomb, the tiling by cells with regular triangles and square shape in the ratio 2:1 [52], can be excluded, because the honeycomb of **2*i*5** is formed from a square honeycomb by side‐group expansion, while the triangle+square tiling is expected for side‐chain volume reduction at the transition from square to triangular honeycombs. Moreover, the triangle + square LC honeycomb always has the *p*4*gm* space group; a *p*2*gg* lattice has never been found in this case [42, 48, 49, 50]. The *p*2*gg* plane group indicates a type‐2 pentagon tiling, which is supported by the experimentally observed phase biaxiality (Figure 4c), while the type‐4 pentagon tiling would be optically uniaxial due to the square *p*4*gm* symmetry. As calculated from the lattice parameters and the molecular volume, about 19 molecules are organized in each unit cell with an assumed *h* = 0.46 nm (Table 2), in line with the honeycomb walls being formed by the stacking of pairs of back‐to‐back arranged molecules (*n*_wall_ = 1.9, see Table 2). The difference between the parameters *a*_rec_ = 6.78 nm and *b*_rec_ = 6.36 nm is relatively small, with *b*_rec_ = being 94% of *a*_rec_, so the pentagon tiling is close to the type‐4 tiling with square symmetry. For this reason, the homeotropic areas have only weak birefringence (Figure 4c).

**FIGURE 4:**
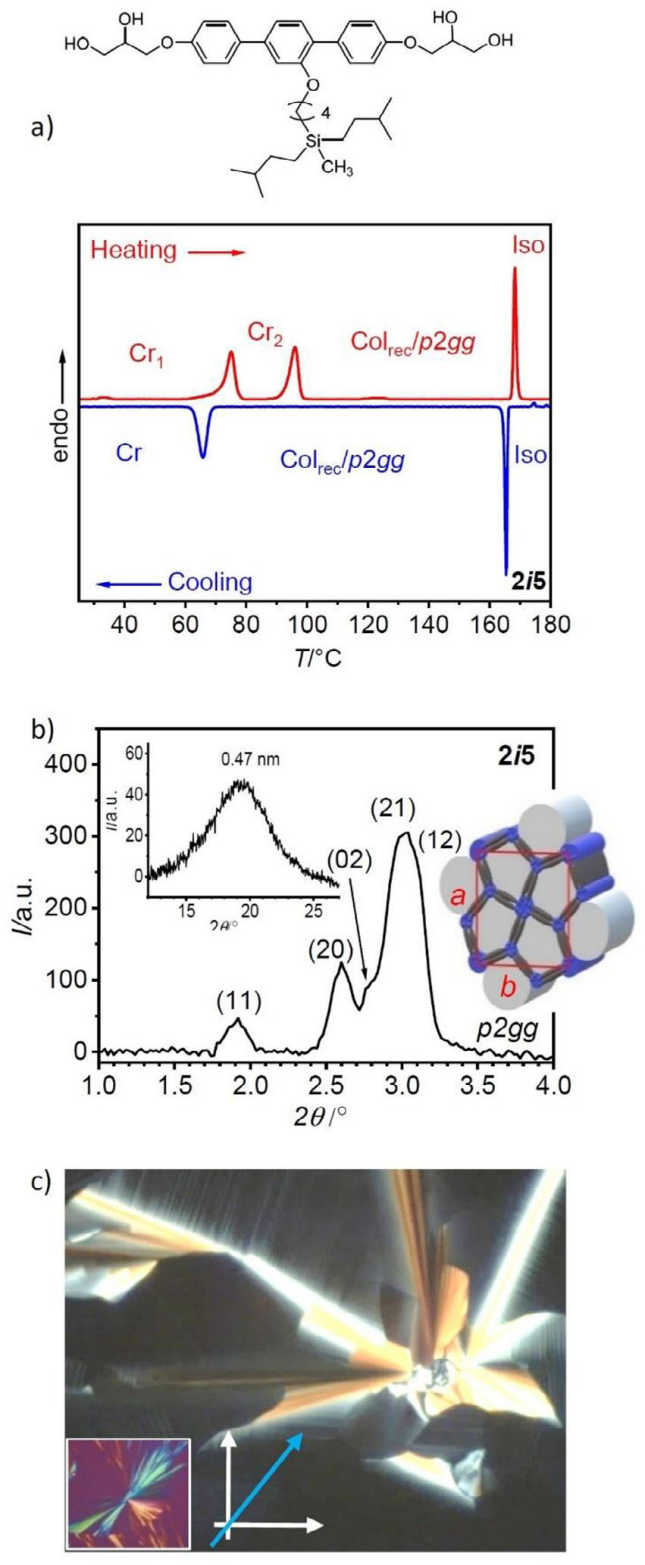
Investigation of compound **2*i*5**; a) molecular structure and second DSC heating and cooling scans at 10 K min^−1^, b) SAXS patterns with insets showing the WAXS of the Col_rec_/*p*2*gg*‐phase at 140°C and the model of the pentagonal honeycomb LC; for numerical X‐ray data, see Table S2; c) texture as observed between crossed polarizers in the Col_rec_/*p*2*gg* phase at 140°C; a weakly birefringent stripe pattern is in line with phase biaxiality; the white arrows indicate the orientations of polarizer and analyzer, while the blue arrow indicates the direction of the indicatrix slow axis of the λ‐plate; for data of compounds **2*i*6**, **2*t*6** and **2*c*8**, see Figures S2–S4 and Table S3–S5.

Remarkably, the pentagonal honeycomb LC with *p*2*gg* lattice is retained for all following compounds with two isohexyl (**2*i*6**), tert‐hexyl (**2*t*6**), and 2‐(cyclohexyl)ethyl groups (**2*c*8**) at the silicon branching point, that is, with growing side‐group volume *V*_R_ from **2*i*5** via **2*i*6** and **2*t*6** to **2c8** (Table 1). For all compounds, the lattice parameter *a*_rec_ is in the same range between *a*_rec_ = 6.32 ‐ 6.40 nm, only marginally increasing from **2*i*6** to **2*c*8**, while the parameter *b*_rec_ decreases from *b*_rec_ = 6.78 to 6.66 with growing side‐group volume. This means that the pentagon deformation decreases with side‐group expansion. From **2*i*5** to **2*c*8,** the unit cell volume (at assumed constant *h* = 0.46 nm) slightly increases from 20.0 nm^3^ to 20.6 nm^3^ while the number of molecules per unit cell decreases from 19.1 to 17.4 (Table 2). This means that with side‐group expansion, the side‐length of the pentagons only marginally changes, and a major adjustment of the cell volume to the side‐group volume is achieved by thinning the honeycomb walls from *n*_wall_ = 1.9 to 1.7 by assuming a staggered packing along the walls (Figure S6b). Figure 5 summarizes the development of the phase transitions depending on effective side‐group length *L*_R_ and side‐group volume *V*_R_. Note that Figure 5 also includes some compounds **3** (indicated with a blue background), but here the focus is on series **2**.

**FIGURE 5:**
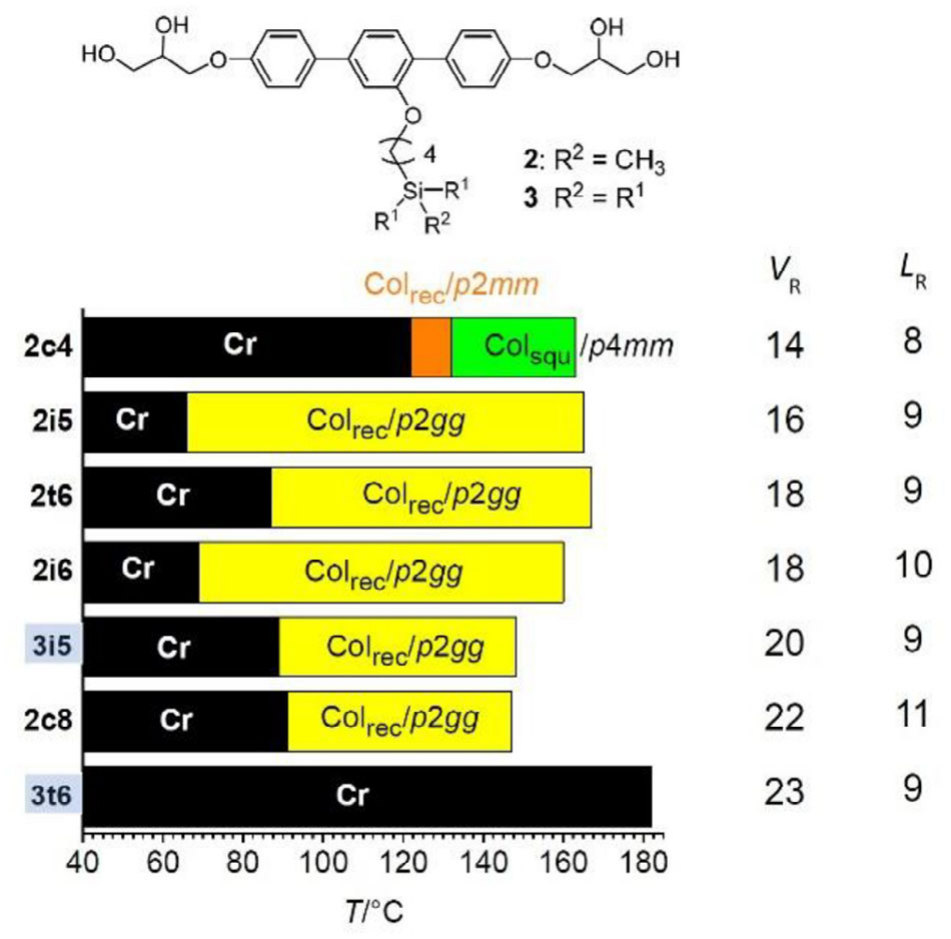
Bar diagram showing the mesophase ranges of the LC compounds of series **2** and **3** (blue background) as observed on heating, preliminary ordered according to growing side‐group volume *V*_R_ and secondly with growing side‐group length *L*_R_; for the structures of the substituents R^1^, see Scheme 1.

#### 2.3.3 Comparison Between Linear Side‐Chains And Branched Side‐Groups

Compared to the related bolapolyphiles **T*n*** [55] with linear *n*‐alkyl chains, the range of the pentagon tiling is expanded for compounds **2**. In the series **T*n*,** it is observed for *n* = *V*_R_ = 12‐14 (see Figure 2a), while in the series **2,** the pentagon range is shifted to larger side‐group volume and the volume range is expanded to *V*_R_ = 16‐22. This shows that expansion of the chain ends by branching is a way to extend the existence range of pentagonal honeycombs. We attribute this to the limitation of the effective length of the side‐groups (*L*_R_) despite the growing volume (*V*_R_). With branched chains, the side‐group length (*L*_R_) and volume (*V*_R_) are decoupled, because chain branching (as well as cyclization) reduces their length at constant volume. Thus, all side‐groups of the pentagon‐forming compounds in series **2** are shorter (*L*_R_ = 8‐11) than the linear chains in the series **T*n*** (*L*_R_ = 12 ‐ 14). The limitation of the side‐group length in the case of compounds with highly branched or cyclic ends limits the distances that can be bridged in the honeycomb cells, that is, it limits their capability to fill the centers of the next larger cells, though it would be required by the volume *V*_R_. Therefore, the pentagon formation is expanded to larger side‐group volume, and it becomes more difficult to reach the next larger polygon (in this case, the hexagon) with these shorter side‐groups. This expands the pentagon range on the expense of the next larger polygonal cells. It is remarkable that the highest stability (highest *p*2*gg*‐Iso transition temperature) of the pentagon tiling is achieved with compound **2*t*6** having the most crowded side‐group of series **2** with two *tert*‐hexyl groups at Si. The decrease of the *p*2*gg*‐Iso transition temperature with further growing *V*_R_ values from **2*t*6** to **2*c*8** indicates that the pentagon honeycomb is destabilized with further increasing side‐group volume. In order to test the limits of pentagonal honeycomb formation, a further increase of *V*_R_ is required. For this purpose, the total number of bulky substituents at Si was increased from two to three as described in the next Section.

### 2.4 Compounds 3 With Three Cyclic or Branched Substituents at the Si Branching Point

In this series of compounds **3**, three identical branched or cyclic groups are attached to Si. The DSC traces of **3*c*4** (Figure S3e) and POM investigations (Figure S1e, f) indicate the formation of two metastable LC phases, Col_squ_/*p*4*mm* and Col_rec_/*p*2*mm*, besides a complicated crystalline polymorphism. The Col_squ_/*p*4*mm* square honeycomb is obviously retained as a small range of a monotropic (metastable) LC phase after replacing CH_3_ of **2*c*4** by the additional cyclobutyl group in **3*c*4** (Table 3). The Col_squ_/*p*4*mm* phase structure is confirmed by X‐ray scattering (Figures S4d, S5d) and optical investigations (Figure S1e), similar as described for **2*c*4**. The lattice parameter *a*_squ_ = 2.59 nm is almost unchanged compared to **2*c*4** despite the increased side‐group volume *V*_R_, because the fixed bolaamphiphilic core length is the major factor determining the polygonal cell size. The *p*4*mm*→Iso transition temperature of this compound **3*c*4** with a tricyclobutylsilyl group is reduced only marginally fom 167°C for **2*c*4** to 163°C for **3*c*4** (Table 3). However, the melting point and the crystallization temperature are significantly enhanced by 42 K/25 K, and for this reason, the square honeycomb becomes metastable (monotropic), only observable upon cooling below the melting point at 171°C (Figure S3e). The high stability of the crystalline phases is likely due to the reduced contribution of flexible alkyl chain segments. POM investigations between crossed polarizers indicate the occurrence of a stripe pattern at 157°C (Figure S1e,f) on cooling, which we attribute to a lattice deformation to a rectangular Col_rec_/*p*2*mm* honeycomb, similar to that observed for **2*c*4**. Due to rapid crystallization, also in this case no XRD confirmation can be presented for the Col_rec_/*p*2*mm* phase.

For **3*i*5** with three isopentyl groups, a Col_rec_/*p*2*gg* phase is found between 141°C and 152°C on heating and down to 89°C on cooling (Table 3, for DSC see Figure 3f, for XRD data see Figures S4c and S5e and Table S7), that is, the temperature range of this pentagonal honeycomb is narrowed compared to **2*i*5**. Similar as found for the cyclobutyl substituted compounds, the transition from **2*i*5** to **3*i*5** raises the melting and crystallization temperatures of the crystalline phases, but in this case, a enantiotropic (stable) phase range of the pentagonal honeycomb is retained, although the LC→Iso transition is reduced by 16 K from **2*i*5** to **3*i*5**. The reduced melting point and crystallization tendency of **3*i*5** compared to **3*c*4** is thought to be due to the increased conformational mobility of the branched isopentyl groups compared to the cyclobutyl groups, thus stabilizing the LC state entropically. Considering the side‐group volume (*V*_R_ = 20), compound **3*i*5** fits well between the compounds **2*i*6** (*V*_R_ = 18) and **2*c*8** (*V*_R_ = 22) of series **2,** in line with the *p*2*gg*‐Iso transition temperature having a value between these two compounds (see Figure 5).

Compound **3*t*6** with three bulky *tert*‐hexyl groups at Si and *V*_R_ = 23 is the first compound of series **3** exceeding the chain volume achieved in series **2**. Compound **3*t*6** has a melting point of 184°C, and no LC phase could be observed before crystallization of this compound takes place at 111°C upon cooling (Table 3, for DSC, see Figure S3d). If there would be a monotropic (metastable) honeycomb phase, its LC‐Iso transition has to be below 111°C. Thus, we can conclude that *V*_R_ = 22 (compound **2*c*8**) represents the maximum side‐group volume to produce the pentagon tiling by LC self‐assembly of *p*‐terphenyl derived bolapolyphiles. Moreover, overcrowding the ends of the side‐group by using three bulky substituents is unfavorable and destabilizes the honeycomb LCs and, more importantly, favours multiple crystalline phases replacing the LC phases. Therefore, further overcrowding the ends of the side‐groups beyond that achieved with **2*c*8** is not favorable for the formation of pentagonal liquid crystalline honeycombs.

## 3 Conclusions

Carbosilane side‐groups with two branched or cyclic substituents and one CH_3_ group at Si provide bolapolyphilic compounds with wide temperature ranges of pentagonal LC honeycombs, while three of them reduce phase ranges or even remove the honeycombs completely, while crystalline phases become dominating. In all cases, the less symmetric type‐2 pentagons are formed. Although pentagon deformation is small and it decreases with growing side‐group volume (see Table 1), in no case a type‐4 pentagon packing with two exact 90° angles and *p*4*gm*‐lattice could be observed in this series of compounds. This dominance of type‐2 pentagons with *p*2*gg* plane group is opposite to the previous observations in LC honeycombs [52, 58, 59, 60, 61] and solid‐state materials [27], where the more symmetric type‐4 pentagon with *p*4*gm* lattice is in most cases dominating. It appears that the *p*4*gm* lattice with higher symmetry and two pairs of identical side lengths is favored if it is difficult to change the length of the polygon sides. In the case of inorganic materials, it is favored by fixed bond lengths and, in addition, fixed bond angles. In contrast, the junctions in the LC honeycombs can assume almost any angle, supporting type‐2 pentagon formation. However, there is also a dependence on the rod length. Bolapolyphiles with long rigid rods and a reduced contribution of the flexible glycerol end groups provide stronger length limitations, thus tending to form type‐4 pentagons, while shorter rods with a larger contribution of the flexible segments prefer type‐2 pentagons. It is noted that the relatively short *p*‐terphenyl core can support the formation of other irregular polygons, too, as previously shown for the case of the non‐regular type‐2 *hexagonal* LC honeycomb with *p*2*gg* plane group [94]. This means that there is the possibility that additional irregular polygonal honeycombs might be achievable with this class of compounds. The easy uniform alignment (dark homeotropic areas of the POM textures where all honeycombs align perpendicular to the substrate surfaces, see Figures S1, S2), and the possibility to change the polygonal shape of nanometer scale tiling patterns by molecular design or by external stimuli provides potential applications, as for example, in nanolithography and template directed self‐assembly for miniaturization of computer chips beyond the scale of present EUV methods.

## 4 Experimental Section

### 4.1 Synthesis

The detailed synthesis procedures (Scheme 1), and the analytical data of compounds **2** and **3** with their intermediates are collated in section S4 and Figures S6–S14.

## Conflicts of Interest

The authors declare no conflict of interest.

## Supporting information



Experimental details, materials synthesis, analytical data, and additional data (POM‐textures, DSCs, XRD and structural data and molecular models).
**Supporting File 1**: chem71595‐sup‐0001‐SuppMat.pdf.

## Data Availability

The data that supports the findings of this study are available in the supporting information of this article.

## References

[1] B. Grünbaum and G. Shephard , Tilings and Patterns (W. H. Freeman, 1987).

[2] C. Zong , “Can You Pave the Plane With Identical Tiles?,” Notices of the American Mathematical Society 67 (2020): 635–646, 10.1090/noti2075.

[3] T. Sugimoto , “Convex Pentagonal Monotiles in the 15 Type Families,'' arXiv:2501.07090v1[math.HO] 13.1. 2025,”.

[4] C. Mann , J. McLoud‐Mann , and D. Von Derau , “Convex Pentagons That Admit *i*‐block Transitive Tilings,” Geometriae Dedicata 194 (2018): 141–167, 10.1007/s10711-017-0270-9.

[5] M. Rao , “Exhaustive Search of Convex Pentagons Which Tile the Plane'' arXiv:1708.00274v1 [math.CO] 1 Aug 2017,”.

[6] Wikimedia Commons. https://commons.wikimedia.org/wiki/File:PentagonTilings15.svg. Creative Commons Attribution‐Share Alike 4.0 International, https://creativecommons.org/licenses/by‐sa/4.0/deed.en.

[7] C. S. Hartley and J. S. Moore , “Programmed Dynamic Covalent Assembly of Unsymmetrical Macrocycles,” Journal of the American Chemical Society 129 (2007): 11682–11683, 10.1021/ja0745963. 17725357

[8] L. Zhao , K. Ghosh , Y. Zheng , M. M. Lyndon , T. I. Williams , and P. J. Stang , “Construction of Coordination‐Driven Self‐Assembled [5 + 5] Pentagons Using Metal−Carbonyl Dipyridine Ligands,” Inorganic Chemistry 48 (2009): 5590–5592, 10.1021/ic900649m. PMC2878388 19476323

[9] X. Q. Zeng , H. M. Li , H. L. Sun , H. Beckers , H. Willner , and H. F. Schaefer , “SN_2_ P_2_: A Neutral Five‐Membered Sulfur–Pnictogen(III) Ring,” Angewandte Chemie International Edition 54 (2015): 1327–1330, 10.1002/anie.201409868. 25475709

[10] T. Nagano , K. Nakamura , Y. Tokimaru , et al., “Functionalization of Azapentabenzocorannulenes by Fivefold C−H Borylation and Cross‐Coupling Arylation: Application to Columnar Liquid‐Crystalline Materials,” Chemistry—A European Journal 24 (2018): 14075–14078, 10.1002/chem.201803676. 30043435

[11] C. Ren , F. Zhou , B. Qin , et al., “Crystallographic Realization of the Mathematically Predicted Densest all‐Pentagon Packing Lattice by _5_ ‐Symmetric “Sticky” Fluoropentamers,” Angewandte Chemie International Edition 50 (2011): 10612–10615, 10.1002/anie.201101553. 21928457

[12] A. Velian and C. C. Cummins , “Synthesis and Characterization of P_2_ N^−^: An Aromatic Ion Composed of Phosphorus and Nitrogen,” Science 348 (2015): 1001–1004, 10.1126/science.aab0204. 26023134

[13] C. S. Campos‐Fernández , R. Clérac , J. M. Koomen , D. H. Russell , and K. R. Dunbar , “Fine‐Tuning the Ring‐Size of Metallacyclophanes: a Rational Approach to Molecular Pentagons,” Journal of the American Chemical Society 123 (2001): 773–774, 10.1021/ja002960r. 11456607

[14] S. Guieu , A. K. Crane , and M. J. MacLachlan , “Campestarenes: Novel Shape‐persistent Schiff Base Macrocycles With 5‐fold Symmetry,” Chemical Communications 47 (2011): 1169–1171, 10.1039/C0CC04493H. 21125107

[15] L. Zoppi , T. Bauert , J. S. Siegel , K. K. Baldridge , and K.‐H. Ernst , “Pentagonal Tiling With Buckybowls: Pentamethylcorannulene on Cu(111),” Physical Chemistry Chemical Physics 14 (2012): 13365, 10.1039/c2cp41732d. 22935627

[16] K. Tahara , T. Balandina , S. Furukawa , S. De Feyter , and Y. Tobe , “Molecular Pentagonal Tiling: Self‐Assemblies of Pentagonal‐Shaped Macrocycles at Liquid/Solid Interfaces,” CrystEngComm 13 (2011): 5551, 10.1039/c1ce05336a.

[17] S.‐S. Jester , E. Sigmund , and S. Höger , “Nanopatterning by Molecular Polygons,” Journal of the American Chemical Society 133 (2011): 11062–11065, 10.1021/ja203536t. 21678962

[18] D. Cui , M. Ebrahimi , J. M. Macleod , and F. Rosei , “Template‐Driven Dense Packing of Pentagonal Molecules in Monolayer Films,” Nano Letters 18 (2018): 7570–7575, 10.1021/acs.nanolett.8b03126. 30403353

[19] B. E. Hirsch , S. Lee , B. Qiao , et al., “Anion‐induced Dimerization of 5‐fold Symmetric Cyanostars in 3D Crystalline Solids and 2D Self‐assembled Crystals,” Chemical Communications 50 (2014): 9827, 10.1039/C4CC03725A. 25080328

[20] I. Nierengarten , S. Guerra , M. Holler , J.‐F. Nierengarten , and R. Deschenaux , “Building Liquid Crystals From the 5‐fold Symmetrical Pillar[5]Arene Core,” Chemical Communications 48 (2012): 8072, 10.1039/c2cc33746k. 22781925

[21] B. Moulton , J. Lu , and M. J. Zaworotko , “Periodic Tiling of Pentagons: the First Example of a Two‐Dimensional ( )‐net,” Journal of the American Chemical Society 123 (2001): 9224–9225, 10.1021/ja016637f. 11552856

[22] S. Zhang , J. Zhou , Q. Wang , X. Chen , Y. Kawazoe , and P. Jena , “Penta‐Graphene: A New Carbon Allotrope,” Proceedings of the National Academy of Sciences 112 (2015): 2372–2377, 10.1073/pnas.1416591112. PMC4345574 25646451

[23] A. D. Oyedele , S. Yang , L. Liang , et al., “PdSe_2_: Pentagonal Two‐Dimensional Layers With High Air Stability for Electronics,” Journal of the American Chemical Society 139 (2017): 14090–14097, 10.1021/jacs.7b04865. 28873294

[24] W. L. Chow , P. Yu , F. Liu , et al., “High Mobility 2D Palladium Diselenide Field‐Effect Transistors With Tunable Ambipolar Characteristics,” Advanced Materials 29 (2017): 1602969, 10.1002/adma.201602969. 28370566

[25] X. Zhang , G. Su , J. Lu , et al., “Centimeter‐Scale Few‐Layer PdS_2_: Fabrication and Physical Properties,” ACS Applied Materials & Interfaces 13, no. 36 (2021): 43063–43074, 10.1021/acsami.1c11824. 34473488

[26] M. Bykov , E. Bykova , A. V. Ponomareva , et al., “Realization of an Ideal Cairo Tessellation in Nickel Diazenide NiN_2_: High‐Pressure Route to Pentagonal 2D Materials,” ACS Nano 15 (2021): 13539–13546, 10.1021/acsnano.1c04325. 34355559

[27] Y. Shen and Q. Wang , “Pentagon‐based 2D Materials: Classification, Properties and Applications,” Physics Reports 964 (2022): 1–42, 10.1016/j.physrep.2022.03.003.

[28] Y. Shen , C. Zhang , and Q. Wang , “Type‐1 Pentagonal Tiling Realized in 2D Penta‐SrP_2_ Sheet,” The Journal of Physical Chemistry Letters 14 (2023): 8734–8740, 10.1021/acs.jpclett.3c02329. 37737655

[29] R. Thakuria , B. Sarma , and A. Nangia , “Guest Control in the Self‐Assembly of H‐Shaped Host to Cyclopentanoid (5,^3^ ) Net,” Crystal Growth & Design 8 (2008): 1471–1473, 10.1021/cg800089v.

[30] L. Shi , Z. Xiong , H. Wang , H. Cao , and Z. Chen , “Quasicrystal Approximants in Isoreticular Metal‐organic Frameworks via Cairo Pentagonal Tiling,” Chemistry 10 (2024): 2464–2472, 10.1016/j.chempr.2024.03.030.

[31] F. Wen , K. Xu , Y. Feng , and N. Huang , “Two‐Dimensional Covalent Organic Frameworks With Pentagonal Pores,” Journal of the American Chemical Society 146 (2024): 19680–19685, 10.1021/jacs.4c06438. 38979969

[32] P.‐J. Tian , X.‐H. Han , Q.‐Y. Qi , and X. Zhao , “Identification of Two‐dimensional Covalent Organic Frameworks With Mcm Topology and Their Application in Photocatalytic Hydrogen Evolution,” Chemical Science 15 (2024): 9669–9675, 10.1039/D4SC01780C. PMC11206236 38939151

[33] Y. Liu , L. Yuan , W. Chi , et al., “Cairo pentagon Tessellated Covalent Organic Frameworks With Mcm Topology for Near‐Infrared Phototherapy,” Nature Communications 15 (2024): 7150, 10.1038/s41467-024-50761-8. PMC11339432 39168967

[34] G. A. Jeffrey and W. Saenger , Hydrogen Bonding in Biological Structures (Springer, 1994).

[35] C. T. Wolke , J. A. Fournier , L. C. Dzugan , et al., “Spectroscopic Snapshots of the Proton‐Transfer Mechanism in Water,” Science 354 (2016): 1131–1135, 10.1126/science.aaf8425. 27934761

[36] E. G. Atovmyan , S. V. Koshchii , and T. N. Fedotova , “Investigation of Hydrogen Bonds in Alkyl Glycerol Ethers,” Journal of Applied Spectroscopy 48 (1988): 202–205, 10.1007/BF00662002.

[37] J. S. Lomas , “Cooperativity in Alkane‐1,2‐ and 1,3‐Polyols: NMR, QTAIM, and IQA Study of O─H… OH and C─H… OH Bonding Interactions,” Magnetic Resonance in Chemistry 58 (2020): 666–684, 10.1002/mrc.5014. 32201981

[38] J.‐H. Fuhrhop and T. Wang , “Bolaamphiphiles,” Chemical Reviews 104, no. 6 (2004): 2901–2938, 10.1021/cr030602b. 15186184

[39] F. Hentrich , C. Tschierske , and H. Zaschke , “Bolaamphiphilic Polyols, a Novel Class of Amphotropic Liquid Crystals,” Angewandte Chemie International Edition in English 30 (1991): 440–441, 10.1002/anie.199104401.

[40] P. J. Collings and J. W. Goodby , Introduction to Liquid Crystals: Chemistry and Physics (CRC Press, 2019), 10.1201/9781315098340.

[41] J. W. Goodby , J. P. Collings , T. Kato , C. Tschierske , H. F. Gleeson , and P. Raynes , eds., Handbook of Liquid Crystals (Wiley‐VCH, 2014), 10.1002/9783527671403.

[42] C. Tschierske , “Development of Structural Complexity by Liquid‐Crystal Self‐Assembly,” Angewandte Chemie International Edition 52 (2013): 8828–8878, 10.1002/anie.201300872. 23934786

[43] C. Tschierske , “Non‐Conventional Liquid Crystals—The Importance of Micro‐segregation for Self‐Organisation,” Journal of Materials Chemistry 8 (1998): 1485–1508, 10.1039/a800946e.

[44] C. Tschierske , “Micro‐segregation, Molecular Shape and Molecular Topology—Partners for the Design of Liquid Crystalline Materials With Complex Mesophase Morphologies,” Journal of Materials Chemistry 11 (2001): 2647–2671, 10.1039/b102914m.

[45] J. Uchida , B. Soberats , M. Gupta , and T. Kato , “Advanced Functional Liquid Crystals,” Advanced Materials 34 (2022): 2109063, 10.1002/adma.202109063. 35034382

[46] H. K. Bisoyi and Q. Li , “Liquid Crystals: Versatile Self‐Organized Smart Soft Materials,” Chemical Reviews 122 (2022): 4887–4926, 10.1021/acs.chemrev.1c00761. 34941251

[47] T. Wöhrle , I. Wurzbach , J. Kirres , et al., “Discotic Liquid Crystals,” Chemical Reviews 116 (2016): 1139–1241. 10.1021/acs.chemrev.5b00190 26483267

[48] C. Tschierske , “Liquid Crystal Engineering—New Complex Mesophase Structures and Their Relations to Polymer Morphologies, Nanoscale Patterning and Crystal Engineering,” Chemical Society Reviews 36 (2007): 1930, 10.1039/b615517k. 17982518

[49] C. Tschierske , C. Nürnberger , H. Ebert , et al., “Complex Tiling Patterns in Liquid Crystals,” Interface Focus 2 (2012): 669–680, 10.1098/rsfs.2011.0087. PMC3438576 24098852

[50] G. Ungar , C. Tschierske , V. Abetz , et al., “Self‐Assembly at Different Length Scales: Polyphilic Star‐Branched Liquid Crystals and Miktoarm Star Copolymers,” Advanced Functional Materials 21 (2011): 1296–1323, 10.1002/adfm.201002091.

[51] Y. Cao , C. Tschierske , and F. Liu , “Structural Complexity, Network Formation and Spontaneous Chirality in Soft‐Matter Self‐Assembly,” Accounts of Chemical Research 59 (2026): 1983–1996, 10.1021/acs.accounts.6c00103. PMC13276857 42109026

[52] B. Chen , X. Zeng , U. Baumeister , G. Ungar , and C. Tschierske , “Liquid Crystalline Networks Composed of Pentagonal, Square, and Triangular Cylinders,” Science 307 (2005): 96–99, 10.1126/science.1105612. 15637274

[53] X.‐H. Cheng , M. Prehm , M. K. Das , et al., “Calamitic Bolaamphiphiles With (Semi)Perfluorinated Lateral Chains: Polyphilic Block Molecules With New Liquid Crystalline Phase Structures,” Journal of the American Chemical Society 125 (2003): 10977–10996, 10.1021/ja036213g. 12952480

[54] A. Lehmann , M. Prehm , C. Chen , et al., “Transition Between Tangential and co‐axial Liquid Crystalline Honeycombs in the Self‐Assembly of Y‐shaped Bolapolyphiles,” Chemical Communications 54 (2018): 12306–12309, 10.1039/C8CC06281A. 30277230

[55] A. Lehmann , A. Scholte , M. Prehm , et al., “Soft Rectangular Sub‐5 Nm Tiling Patterns by Liquid Crystalline Self‐Assembly of T‐Shaped Bolapolyphiles,” Advanced Functional Materials 28 (2018): 1804162, 10.1002/adfm.201804162.

[56] B. Chen , U. Baumeister , G. Pelzl , et al., “Carbohydrate Rod Conjugates: Ternary Rod−Coil Molecules Forming Complex Liquid Crystal Structures,” Journal of the American Chemical Society 127 (2005): 16578–16591, 10.1021/ja0535357. 16305247

[57] A. G. Cook , U. Baumeister , and C. Tschierske , “Supramolecular Dendrimers: Unusual Mesophases of Ionic Liquid Crystals Derived From Protonation of DAB Dendrimers With Facial Amphiphilic Carboxylic Acids,” Journal of Materials Chemistry 15 (2005): 1708, 10.1039/b415892j.

[58] H. Dao , Y. Ye , L. Kong , et al., “Dithiophene Based X‐shaped Bolaamphiphiles: Liquid Crystals With Single Wall Honeycombs and Geometric Frustration,” Soft Matter 8 (2012): 10921–10931.

[59] X. Cheng , X. Dong , G. Wei , M. Prehm , and C. Tschierske , “Liquid‐Crystalline Triangle Honeycomb Formed by a Dithiophene‐Based X‐Shaped Bolaamphiphile,” Angewandte Chemie International Edition 48 (2009): 8014–8017, 10.1002/anie.200903247. 19757469

[60] X. Cheng , F. Liu , X. Zeng , et al., “Influence of Flexible Spacers on Liquid‐Crystalline Self‐Assembly of T‐Shaped Bolaamphiphiles,” Journal of the American Chemical Society 133 (2011): 7872–7881, 10.1021/ja200822z. 21534565

[61] X. Cheng , X. Dong , R. Huang , et al., “Polygonal Cylinder Phases of 3‐Alkyl‐2,5‐diphenylthiophene‐Based Bolaamphiphiles: Changing Symmetry by Retaining Net Topology,” Chemistry of Materials 20 (2008): 4729–4738, 10.1021/cm800509u.

[62] S. Poppe , C. Chen , Y. Cao , F. Liu , and C. Tschierske , “From Columns to Networks: Search Toward the Elusive Single Gyroid With π ‐Shaped Polyphilic Liquid Crystalline Block Molecules,” Small Science 5 (2025): 2500157, 10.1002/smsc.202500157. PMC12257908 40666048

[63] R. Kieffer , M. Prehm , B. Glettner , et al., “X‐Shaped Polyphilics: Liquid Crystal Honeycombs with Single‐Molecule Walls,” Chemical Communications 44 (2008): 3861–3863, 10.1039/b804945a. 18726015

[64] F. Liu , R. Kieffer , X. Zeng , et al., “Arrays of Giant Octagonal and Square Cylinders by Liquid Crystalline Self‐Assembly of X‐shaped Polyphilic Molecules,” Nature Communications 3 (2012): 1104, 10.1038/ncomms2096. 23033080

[65] B. Glettner , F. Liu , X. Zeng , et al., “Liquid‐Crystalline Kagome,” Angewandte Chemie International Edition 47 (2008): 9063–9066, 10.1002/anie.200802957. 18924222

[66] S. Poppe , A. Lehmann , M. Steimecke , et al., “Reticular Liquid Crystal Design: Controlling Complex Self‐Assembly of p‐Terphenyl Rods by Side‐Chain Engineering and Chirality,” Giant 18 (2024): 100254, 10.1016/j.giant.2024.100254.

[67] M. Poppe , C. Chen , F. Liu , M. Prehm , S. Poppe , and C. Tschierske , “Emergence of Tilt in Square Honeycomb Liquid Crystals,” Soft Matter 13 (2017): 4676–4680, 10.1039/C7SM00776K. 28671196

[68] M. Poppe , C. Chen , S. Poppe , F. Liu , and C. Tschierske , “A Periodic Dodecagonal Supertiling by Self‐Assembly of Star‐Shaped Molecules in the Liquid Crystalline State,” Communications Chemistry 3 (2020): 70, 10.1038/s42004-020-0314-1. PMC9814142 36703439

[69] M. Poppe , C. Chen , F. Liu , S. Poppe , and C. Tschierske , “Emergence of Uniform Tilt and π‐stacking in Triangular Liquid Crystalline Honeycombs,” Chemical Communications 57 (2021): 6526–6529, 10.1039/D1CC02556B. 34105554

[70] S. Poppe , A. Lehmann , A. Scholte , et al., “Zeolite‐Like Liquid Crystals,” Nature Communications 6 (2015): 8637, 10.1038/ncomms9637. PMC4639914 26486751

[71] A. Kreyes , M. Amirkhani , I. Lieberwirth , et al., “The Longest *β*‐Unsubstituted Oligothiophenes and Their Self‐Assembly in Solution,” Chemistry of Materials 22 (2010): 6453–6458, 10.1021/cm1027095.

[72] W. H. de Jeu , K. Rahimi , U. Ziener , et al., “Substituted Septithiophenes With End Groups of Different Size: Packing and Frustration in Bulk and Thin Films,” Langmuir 32 (2016): 1533–1541. 10.1021/acs.langmuir.5b04371 26807677

[73] N. Hu , R. Shao , C. Zhu , et al., “Ferroelectric and Antiferroelectric Odd–even Behavior in a Tricarbosilane‐terminated Liquid Crystal Homologous Series,” Chemical Science 5 (2014): 1869–1874, 10.1039/C3SC53353K.

[74] C. Keith , R. A. Reddy , H. Hahn , H. Lang , and C. Tschierske , “The Carbosilane Unit as a Stable Building Block for Liquid Crystal Design: A New Class of Ferroelectric Switching Banana‐Shaped Mesogens,” Chemical Communications 40 (2004): 1898, 10.1039/b407890j. 15340590

[75] Y. Zhang , U. Baumeister , C. Tschierske , M. J. O'Callaghan , and C. Walker , “Achiral Bent‐Core Molecules With a Series of Linear or Branched Carbosilane Termini: Dark Conglomerate Phases, Supramolecular Chirality and Macroscopic Polar Order,” Chemistry of Materials 22 (2010): 2869–2884, 10.1021/cm100134f.

[76] R. J. Mandle , E. J. Davis , C.‐C. A. Voll , D. J. Lewis , S. J. Cowling , and J. W. Goodby , “Self‐Organisation Through Size‐Exclusion in Soft Materials,” Journal of Materials Chemistry C 3 (2015): 2380–2388, 10.1039/C4TC02991G.

[77] L. Fritsch , R. J. Mandle , S. J. Cowling , and J. W. Goodby , “Carbosilane Terminated Alkoxycyanobiphenyls for Bistable Scattering Mode LCDs,” Journal of Materials Chemistry C 9 (2021): 714–718, 10.1039/D0TC05082B.

[78] C. P. J. Schubert , C. Müller , A. Bogner , F. Giesselmann , and R. P. Lemieux , “Design of Liquid Crystals With ‘de Vries‐Like’ properties: Structural Variants of Carbosilane‐Terminated 5‐Phenylpyrimidine Mesogens,” Soft Matter 13 (2017): 3307–3313, 10.1039/C7SM00355B. 28418044

[79] J. Risse , A. Jakobi , W. Weissflog , D. Lose , and S. Diele , “Influence of Silicon on Phase Behavior of Low‐Molecular Liquid Crystals,” Proceedings of the Spie 3319 (1998): 82, 10.1117/12.301257.

[80] C. Anders , Y. Cao , F. Liu , et al., “Photoswitchable Carbosilane‐Based Polycatenars Forming Nematic, Tilted Smectic, Hexatic, and Cubic Liquid Crystalline Phases,” Journal of Materials Chemistry C (2026): 7626–7640, 10.1039/d6tc00353b.

[81] S. A. Ponomarenko , N. I. Boiko , V. P. Shibaev , et al., “Carbosilane Liquid Crystalline Dendrimers: from Molecular Architecture to Supramolecular Nanostructures,” Macromolecules 33 (2000): 5549–5558, 10.1021/ma0001032.

[82] I. M. Saez , J. W. Goodby , and R. M. Richardson , “A Liquid‐Crystalline Silsesquioxane Dendrimer Exhibiting Chiral Nematic and Columnar Mesophases,” Chemistry—A European Journal 7 (2001): 2758–2764, 10.1002/1521-3765(20010702)7:13<2758::AID-CHEM2758>3.0.CO;2-T. 11486950

[83] H. Hahn , C. Keith , H. Lang , R. Amaranatha Reddy , and C. Tschierske , “First Example of a Third‐Generation Liquid‐Crystalline Carbosilane Dendrimer With Peripheral Bent‐Core Mesogenic Units: Understanding of “Dark Conglomerate Phases”,” Advanced Materials 18 (2006): 2629–2633, 10.1002/adma.200600161.

[84] D. Apreutesei and G. M. Mehl , “Bent‐core Systems Based on [1,3,4]Oxadiazole Core With Shape Biaxiality,” Revue Roumaine de Chimie 53 (2008): 527–533.

[85] Y.‐C. Chiang , H.‐C. Wu , H.‐F. Wen , et al., “Tailoring Carbosilane Side Chains Toward Intrinsically Stretchable Semiconducting Polymers,” Macromolecules 52 (2019): 4396–4404, 10.1021/acs.macromol.9b00589.

[86] R. Kieffer , M. Prehm , K. Pelz , et al., “Siloxanes and Carbosilanes as New Building Blocks for T‐shaped Bolaamphiphilic LC Molecules,” Soft Matter 5 (2009): 1214, 10.1039/b818871h.

[87] C. Anders , T. Tan , V.‐M. Fischer , et al., “Engineering “Meso‐Atom” Bonding: Honeycomb‐Network Transitions in Reticular Liquid Crystals,” Aggregate 6 (2025): e728, 10.1002/agt2.728.

[88] C. Anders , M. Wagner , M. Alaasar , et al., “Highly Branched Bolapolyphilic Liquid Crystals With a Cubic A15 Network at the Triangle‐Square Transition,” Chemical Communications 60 (2024): 1023–1026, 10.1039/D3CC05247H. 38173419

[89] C. Anders , M. Wagner , S. Poppe , and C. Tschierske , “2D and 3D Nets by Self‐Assembly of Carbosilane Functionalized Polyphilic Liquid Crystals,” Zeitschrift für anorganische und allgemeine Chemie, (2026): e70193, 10.1002/zaac.70193.

[90] R. L. I.‐Y. Bao , R. Zhao , and L. Shi , “Progress and Developments in the Turbo Grignard Reagent i‐PrMgCl·LiCl: A Ten‐year Journey,” Chemical Communications 51 (2015): 6884–6900. 10.1039/c4cc10194d 25714498

[91] A. Kreyes , A. Mourran , Z. Hong , et al., “Predictability of Thermal and Electrical Properties of End‐Capped Oligothiophenes by a Simple Bulkiness Parameter,” Chemistry of Materials 25 (2013): 2128–2136, 10.1021/cm400702t.

[92] A. Immirzi and B. Perini , “Prediction of Density in Organic Crystals,” Acta Crystallographica Section A 33 (1977): 216–218, 10.1107/S0567739477000448.

[93] A. I. Kitaigorodski , “ *Molekülkristalle*, Akademieverlag: Berlin, Germany, 1979,”.

[94] C. Anders , S. Poppe , Y. Cao , F. Liu , and C. Tschierske , “A Self‐Assembled Fluid Honeycomb of Nonsymmetric Hexagons Formed by Nine Molecules in the Circumference,” Small 21 (2025): e05094, 10.1002/smll.202505094. PMC12508708 40847882

