# Supplementary material for: Bolapolyphiles With Bulky Carbosilane‐Based Lateral Groups—Reticular Design of Liquid Crystalline Pentagonal Honeycombs
Source: Chemistry. 2026 Aug 19;32(42):e71595. doi: 10.1002/chem.71595 (PMC13755177; doi:10.1002/chem.71595)
Supplement: Supplementary file 1 [file CHEM-32-e71595-s001.pdf]

Supporting Information

Bolapolyphilles with Bulky Carbosilane-based Lateral Groups – Reticular Design of Liquid Crystalline Pentagonal Honeycombs

Christian Anders, Matthias Wagner, Silvio Poppe, Carsten Tschierske

Institute of Chemistry, Martin Luther University Halle-Wittenberg,
Kurt-Mothes-Straße 2, 06120 Halle, Germany

Content

1. Investigations S2
2. Additional Data S3
2.1 Additional POM textures S3
2.2 Additional DSC Data S5
2.3 X-ray Data S6
2.3.1 Additional SAXS Patterns S6
2.3.2 Additional WAXS Patterns S7
2.3.3 Numerical SAXS data S8
3. Synthesis and Analytical Data S10
3.1 General S10
3.2 Synthesis of Intermediates S11
3.2.1 Synthesis of Carbosilanes **H2** and **H3** S11
3.2.2 Synthesis of (4-bromobutyl)dialkylmethylsilanes **Br2** and (4-bromobutyl)trialkylsilanes **Br3** S12
3.2.3 Synthesis of the acetonides **2A and 3A** S13
3.3 Synthesis of Compounds **2** and **3** S15
3.4. NMR Spectra S19
References S27

1. Investigations

Polarizing Optical Microscopy (POM)

Optical textures of all compounds were characterized by polarizing optical microscopy (Olympus BX51-P) with the combination of a FP82-HAT heating stage and controller FP90 from METTLER. Optical investigations were carried out under equilibrium conditions between two glass slides that were used without further treatment. A full wavelength retardation plate was used to determine the sign of birefringence. Texture photographs were taken with a LEICA MC123 HD camera.

DSC measurements

Transition enthalpies were determined as obtained from differential scanning calorimetry (DSC) which were recorded on a DSC-8000 (Perkin Elmer) in sealed 30 μL aluminum pans with heating and cooling rates of 10 K/min under an N_2 stream.

X-ray scattering

X-ray investigations were performed on a cross-table equipped with a Kristallofles 760H (Siemens Bruker, Cu-Kα radiation, λ = 1.54 Å). A small droplet of the sample is placed on a glass plate. The samples were held on an in-house built temperature-controlled heating stage and the diffraction patterns were recorded with a Vantec 500 area detector (Bruker AXS, Karlsruhe); the exposure time was 15 min for WAXS and 30 min for SAXS. The distance between the sample and the detector was 9.0 cm (WAXS) or 26.9 cm (SAXS), and the beam was parallel to the substrate. Silver behenate was used to calibrate the instrument. Data analysis and transformation to 1D plots was performed with GADDS software.

2. Additional Data

2.1. Additional POM textures

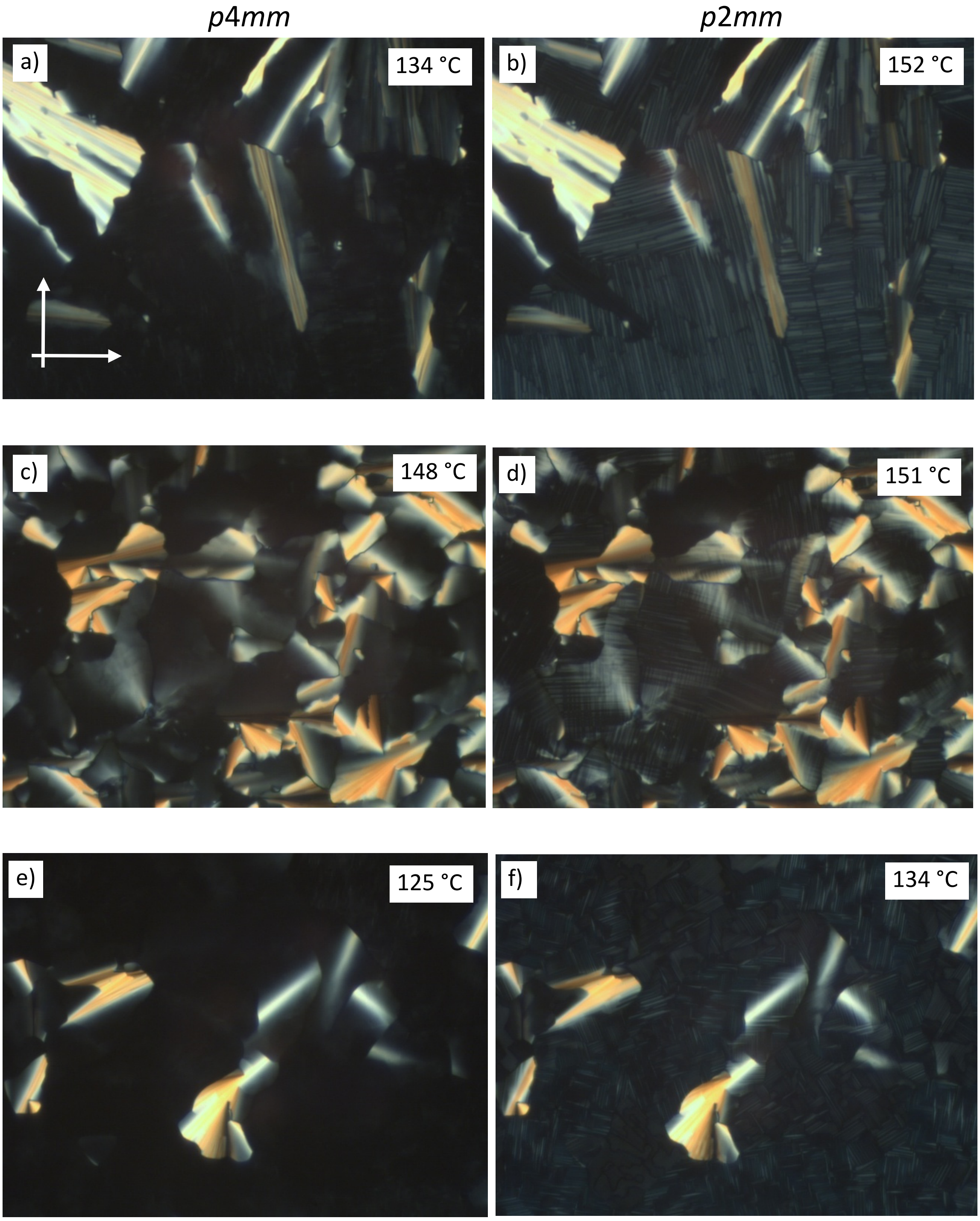

Figure S1. POM textures between crossed polarizers showing the *p*4*mm* (left) to *p*2*mm* (right) transition, indicated by the appearance of a stripe pattern, as observed upon cooling from *p*4*mm* for a-d) compound **2*c*4** and e, f) compound **3*c*4** at the indicated temperatures; direction of polarizer and analyzer for all cases is indicated in a).

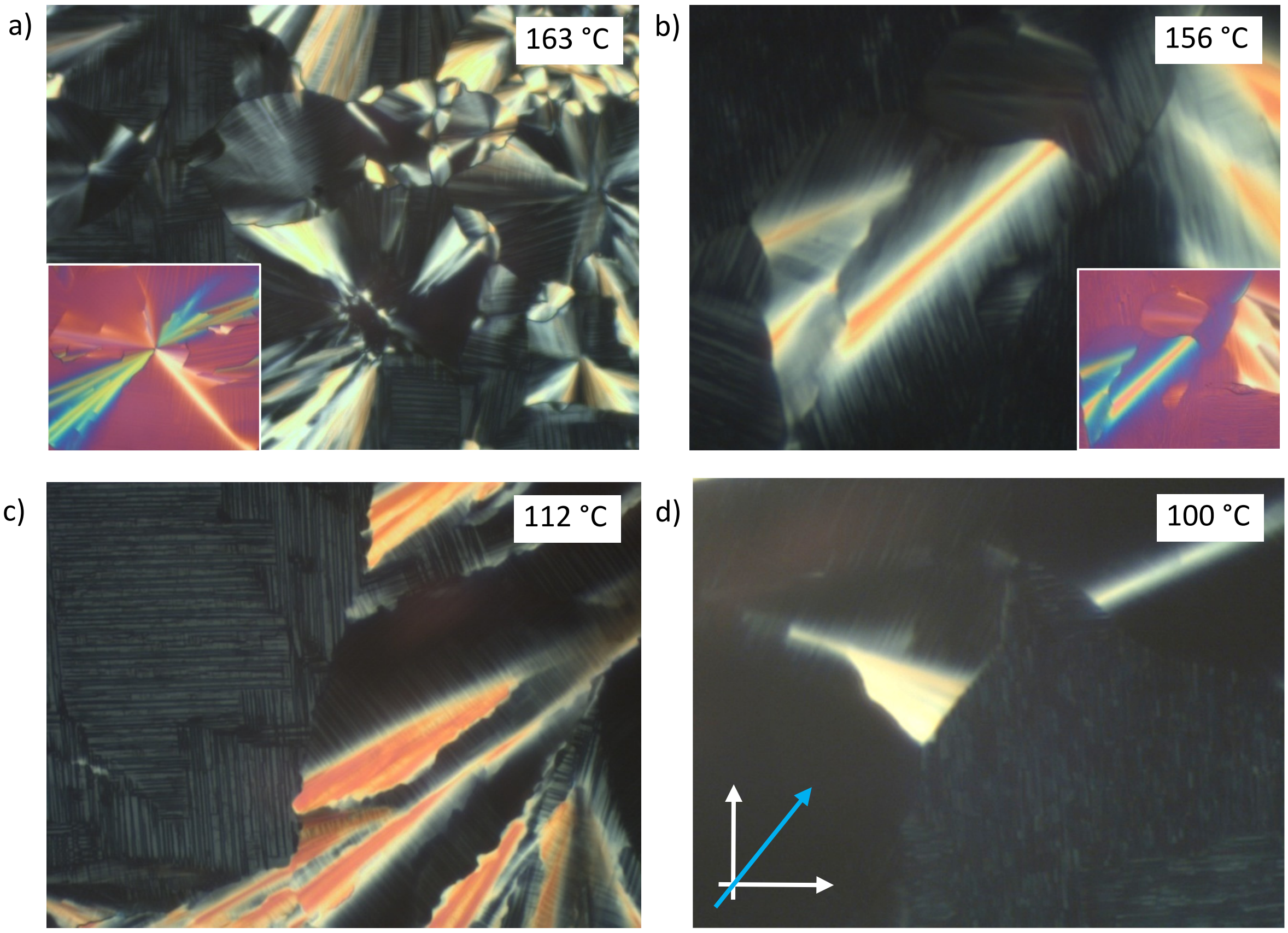

Figure S2. POM textures of the Col_{rec}/*p*2*gg* phases (pentagonal honeycombs) of compounds a) **2*i*6**, b) **2*t*6**, c) **2*c*8** and d) **3*i*5** at the indicated temperatures, the polarizer and indicatrix direction are shown in d) which is valid for all textures. Insets in a,b) show the texture with λ-retarder plate, indication negative birefringence.

2.2. Additional DSC traces

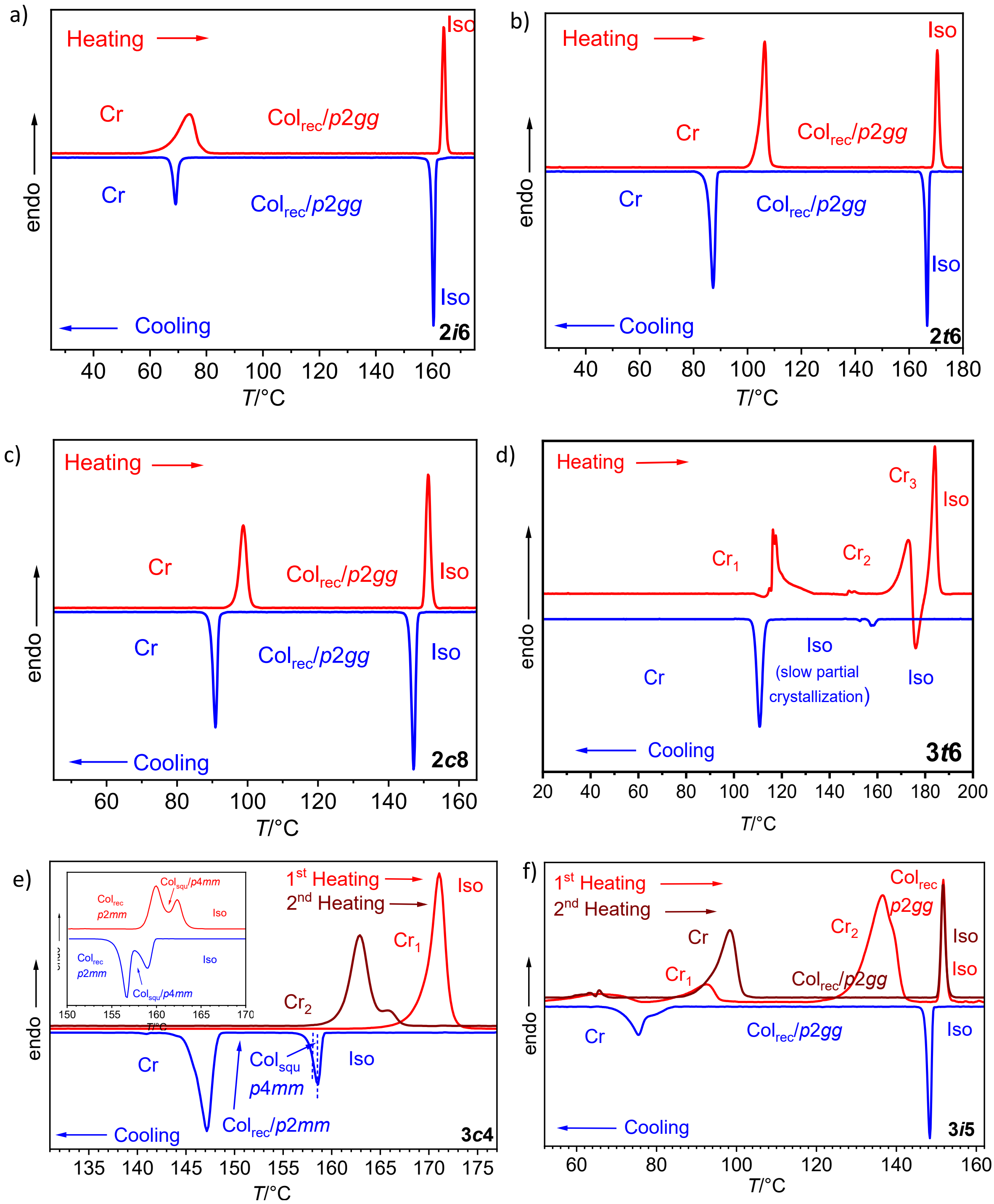

Figure S3. DSC heating and cooling traces (10 Kmin^{-1}) of compounds **2*i*6, 2*t*6**, **2*c*8**, **3*t*6, 3*c*4** and **3*i*5**; in a-d) the first heating and cooling curves are shown, in e, f) the first and second heating and the first cooling scans are shown; the inset in e) shows the heating and cooling curves after cooling to 150 °C and re-heating for the *p*2*mm*-*p*4*mm*-Iso transition range.

2.3. XRD data

2.3.1. Additional SAXS patterns

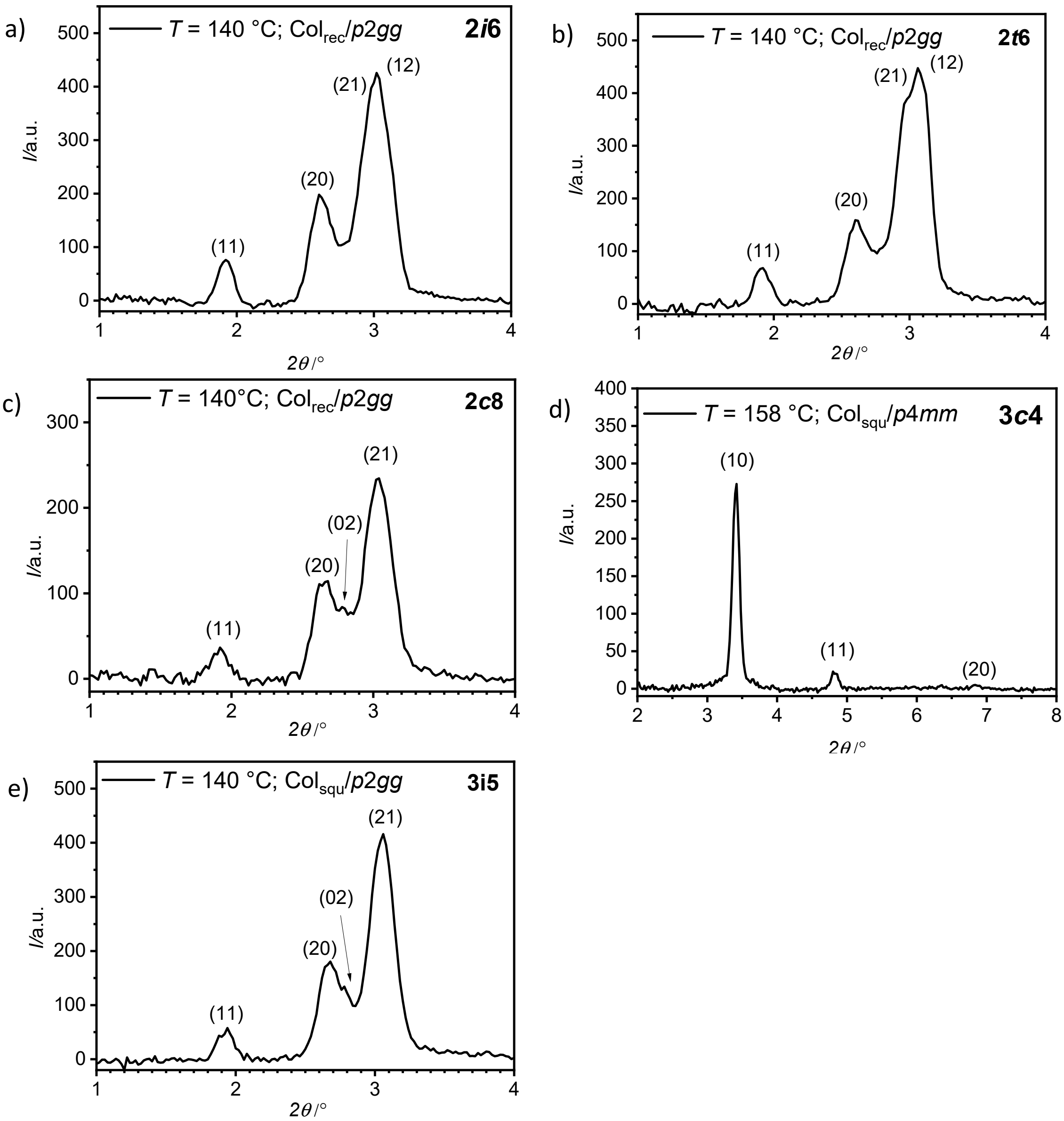

Figure S4. SAXS patterns of compounds **2*i*6**, **2*t*6**, **2*c*8**, **3c4** and **3*i*5** at the indicated temperatures at the indicated temperatures.

2.3.2. Additional WAXS patterns

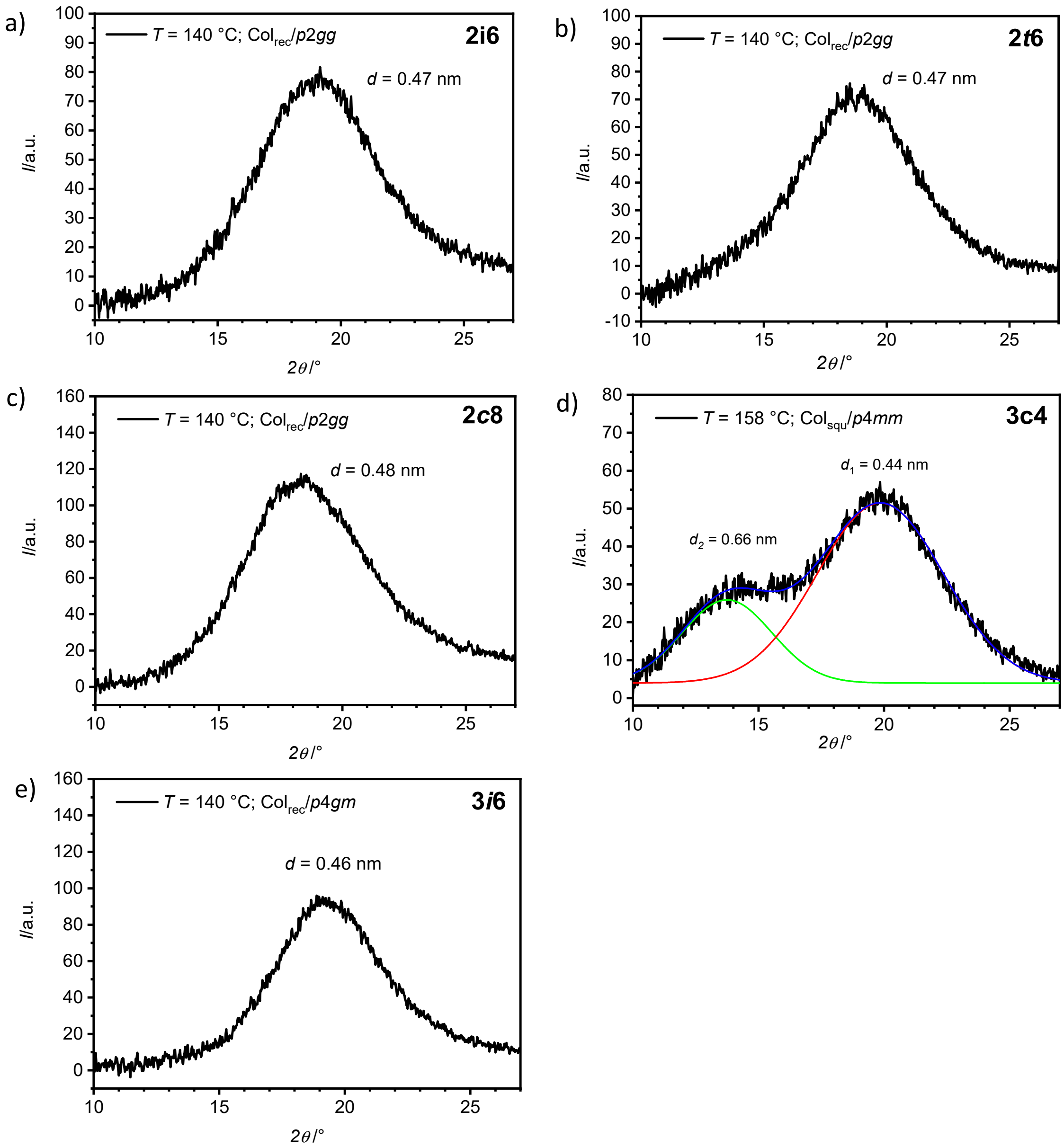

Figure S5. WAXSs of compounds **2*i*6**, **2*t*6**, **2*c*8** **3*c*4** and **3*i*5** at the indicated temperatures at the indicated temperatures.

2.3.3. Numerical SAXS data

Table S1. Experimental and calculated *d*-spacing for the obtained SAXS reflections of the Col_{squ}/*p*4*mm* phase of compound **2*c*4** at *T* = 140 °C.

$2\theta/°$	*d*/nm	d_{calc}/nm	Δ	hk
3.352	2.636	2.636	0.00	10
4.741	1.864	1.864	0.00	11
a = 2.64 nm				

Table S2. Experimental and calculated *d*-spacing for the obtained SAXS reflections of the Col_{rec}/*p*2*gg* phase of compound **2*i*5** at *T* = 140 °C.

$2\theta/°$	*d*/nm	d_{calc}/nm	Δ	hk
1.904	4.639	4.639	0.00	11
2.606	3.390	3.390	0.00	20
2.768	3.191	3.180	0.01	02
2.987	2.958	2.992	0.03	21
3.116	2.835	2.879	0.04	12
a = 6.78 nm, *b* = 6.36 nm				

Table S3. Experimental and calculated *d*-spacing for the obtained SAXS reflections of the Col_{rec}/*p*2*gg* phase of compound **2*i*6** at *T* = 140 °C.

$2\theta/°$	*d*/nm	d_{calc}/nm	Δ	hk
1.915	4.613	4.613	0.00	11
2.620	3.372	3.372	0.00	20
2.989	2.956	2.975	0.02	21
3.100	2.850	2.863	0.01	12
a = 6.74 nm, *b* = 6.32 nm				

Table S4. Experimental and calculated *d*-spacing for the obtained SAXS reflections of the Col_{rec}/*p*2*gg* phase of compound **2*i*5** at *T* = 140 °C.

$2\theta/°$	*d*/nm	d_{calc}/nm	Δ	hk
1.913	4.619	4.619	0.00	11
2.632	3.357	3.357	0.00	20
2.994	2.951	2.972	0.02	21
3.112	2.839	2.887	0.05	12
a = 6.71 nm, *b* = 6.37 nm				

Table S5. Experimental and calculated *d*-spacing for the obtained SAXS reflections of the Col_{rec}/*p*2*gg* phase of compound **2*c*8** at *T* = 140 °C.

$2\theta/°$	*d*/nm	d_{calc}/nm	Δ	hk
1.915	4.613	4.613	0.00	11
2.653	3.330	3.330	0.00	20
2.784	3.174	3.198	0.02	02
3.035	2.911	2.954	0.04	21
a = 6.66 nm, *b* = 6.40 nm				

Table S6. Experimental and calculated *d*-spacing for the obtained SAXS reflections of the Col_{squ}/*p4mm* phase of compound **3*c*4** at *T* = 158 °C.

2θ/°	*d/nm*	d_{calc}/nm	Δ	hk
3.411	2.590	2.590	0.00	10
4.824	1.832	1.831	0.00	11
6.843	1.292	1.295	0.00	20
a = 2.59 nm				

Table S7. Experimental and calculated *d*-spacing for the obtained SAXS reflections of the Col_{squ}/*p2gg* phase of compound **3*i*5** at *T* = 140 °C.

2θ/°	*d/nm*	d_{calc}/nm	Δ	hk
1.927	4.584	4.584	0.00	11
2.697	3.276	3.276	0.00	20
2.789	3.168	3.208	0.04	02
3.048	2.899	2.918	0.02	21
a = 6.42 nm, *b* = 6.55 nm				

Table S8. Structural data for the Col_{squ}/*p4mm* and Col_{rec}/*p2gg* phases of compounds **2** and **3**.

Comp.	*T* / °C (phase)	V_{mol} /nm³	V'_R /nm³	L'_R/nm	*a,b* /nm	A_{cell} / nm²	V_{cell} / nm³	n_{cell}	n_{wall}
**2c4**	140 (*p*4*mm*)	0.86	0.35	1.43	2.64	6.97	3.21	3.3	1.7
**2i5**	140 (*p*2*gg*)	0.94	0.43	1.69	6.78<br>6.36	43.12	19.84	18.8	1.9
**2i6**	140 (*p*2*gg*)	0.99	0.48	1.84	6.74<br>6.32	42.60	19.59	17.7	1.8
**2t6**	140 (*p*2*gg*)	0.99	0.48	1.69	6.71<br>6.37	42.74	19.66	17.7	1.8
**2c8**	140 (*p*2*gg*)	1.06	0.55	1.75	6.66<br>6.40	42.62	19.61	16.5	1.7
**3c4**	158 (*p*4*mm*)	0.92	0.41	1.43	2.59	6.71	3.09	3.0	1.5
**3i5**	140 (*p*2*gg*)	1.04	0.53	1.69	6.42<br>6.55	42.05	19.34	16.6	1.7

[a] V_{mol} was calculated with the crystal volume increments of Immirzi and Perini;[S1] V'_R is the volume of the side chains as calculate with crystal volume increments and L'_R is the maximum length of the side chains as measured with space filling CPK molecular models; $A_{cell} = a^2$ for Col_{squ} and $a*b$ for Col_{rec}; $V_{cell} = a^2 \cdot h$ for Col_{squ}, $V_{cell} = a \cdot b \cdot h$ for Col_{rec} (h = 0.46 nm; this value is an average value for LC phases of compounds with carbosilane chains); n_{cell} was determined according to $n_{cell} = 0.893 \cdot (V_{cell}/V_{mol})$, where 0.893 is the correction factor for the packing density in the LC state. $n_{wall} = n_{cell}/2$ for Col_{squ}/*p4mm*, and $n_{wall} = n_{cell}/10$ for Col_{rec}/*p2gg*.

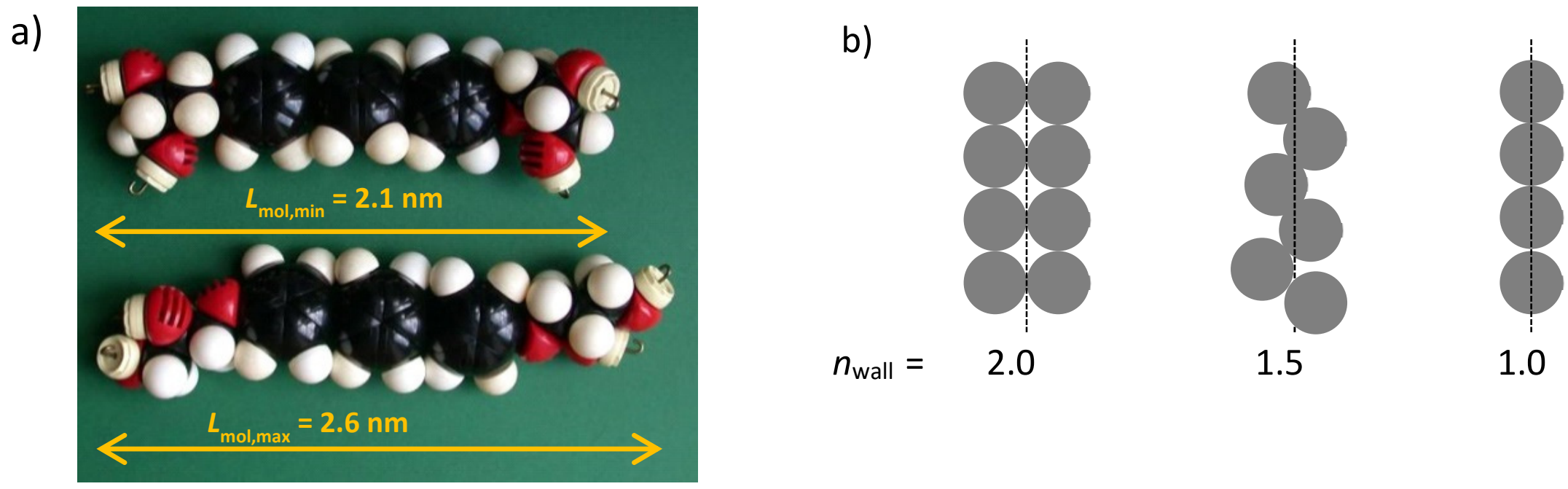

Figure S6. a) Space-filling CPK models showing the length of the bolaamphiphilc core (L_{min} = 2.1 nm and *Lmax* = 2.6 nm) and b) cross-sections through honeycomb walls with different values of n_{wall}.[S2]

3. Synthesis and analytical data

3.1. General

Synthesis of the compounds was performed according to Scheme 1 using the procedures described below. Unless otherwise noted, all starting materials were purchased from commercial sources and were used without further purification. 4,4''-Bis[(2,2-dimethyl-1,3-dioxolane-4-yl)methoxy]-*p*-terphenyl-2'-ol (**1**) was synthesized as reported in ref. [S3]

Dichloromethylsilane, (2-bromoethyl)cyclohexane, 1-bromo-3,3-dimethylbutane, 1-bromo-4-methylpentane, isopentyl magnesium bromide 2M in Et_2O, bromocyclobutane, 4-bromo-1-butene, manganese tunings, and PPTS were obtained from Sigma-Aldrich. 1,2-Dibromoethane was received from ABCR. All compounds were used as received. All compounds were synthesized from racemic rac-1,2-*O*-isopropylidene glycerol, and therefore, **Si*n*** represent racemic mixtures of diastereomers.

The solvents used were purified and dried according to standard procedures.[S4] For column chromatography, silica gel 60 (particle size 0.04–0.063 mm) from MERK was used, as well as glass frits with the dimensions *l* = 7 cm, Ø = 6.5 cm, column height h = *5* cm, *l* = 9 cm, Ø = 10 cm, column height *h* = 7 cm, and columns with the dimensions: *l* = 64 cm, Ø = 3 cm, column height *h* = 60 cm. Silica gel-coated aluminum foils (Silica Gel 60 F254, MERCK) were used for analytical thin-layer chromatography. The substances were detected using UV light at wavelengths of 254 nm or 366 nm, or by spraying with cerium molybdate phosphoric acid. All yields reported refer to the amounts of substance obtained after distillation or recrystallization and are not optimized.

The identity of the compounds was confirmed by ^{1}H, ^{13}C, (and ^{29}Si) NMR spectroscopy on Agilent Technologies 400 MHz VNMRS and Agilent Technologies 500 MHz DDS instruments. Unless otherwise stated, all NMR spectroscopic measurements were performed at a temperature of 27 °C. Tetramethylsilane (TMS) served as the standard.

Mass HR-MS was performed using a BRUKER HR-ESI-TOF instrument in positive mode. For this purpose, the samples were dissolved in THF (1 mg/mL) and mixed with LiCl (0.1 mg/mL).

3.2. Synthesis of intermediates

3.2.1 Synthesis of carbosilanes H2 and H3 [S5]

All procedures are carried out under an Ar gas atmosphere. For the preparation of the Grignard reagent, magnesium (1.5 eq per Cl substituent) is placed in a two-neck flask in dry THF (4 mL/mmol). To generate a reactive surface on the magnesium, 1,2-dibromoethane (1.0 mL) is slowly added dropwise to the reaction mixture. The corresponding bromoalkane (1.25 eq per Cl substituent) is then slowly added dropwise. After the reaction has subsided, the reaction mixture is cooled to 0 °C. To this solution or a commercially available Grignard solution, dry LiCl (3 mol%) was added. For isopentyl magnesium bromide solution in Et_2O was used without added LiCl. To this solution, dichloromethylsilane (1.0 eq) was added dropwise with stirring, and slowly warmed up to room temperature. After 12 hours, the reaction is quenched with water (20 mL). The phases are separated, the aqueous phase is extracted with diethyl ether (3 × 50 mL), and the combined organic extracts are washed with water (50 mL) and brine (50 mL). The organic phase is dried over sodium sulfate. After the solvent has been distilled off under reduced pressure (40 °C, 850 mbar), the product is purified by column chromatography. (Eluent: *n*-hexane)

Dicyclobutyl(methyl)silane H2c4:[S6] Synthesized from bromocyclobutane (2.5 aq., 3.4 mL, 35.6 mmol), dichloromethylsilane (1.5 mL, 14.2 mmol) purification by column chromatography (eluent: *n*-hexane); colorless liquid, $C_9H_{18}Si$ M = 154.32 g/mol, yield: 0.7 g; 4.5 mmol (32 %), **^{1}H-NMR** (400 MHz, $CDCl_3$): δ / ppm = 3.86 – 3.65 (m, 1H, Si-*H*), 2.22 – 2.06 (m, 8H, -C*H₂*-), 2.06 – 1.81 (m, 6H, -C*H₂*-, Si-C*H*-), 0.04 (d, $^3J_{H,H}$ = 3.7 Hz, 3H, Si-C*H₃*). **^{29}Si-NMR** (80 MHz, $CDCl_3$): δ / ppm = -5.96 (s, *Si*).

Methylbis(3-methylbutyl)silane H2i5:[S7] Synthesized from isopropylmagnesium bromide 2.0M in Et_2O, dichloromethylsilane (21.7 mL, 43.5 mmol) purification by column chromatography (eluent: *n*-hexane); colorless liquid, $C_{11}H_{26}Si$ M = 186.41 g/mol, yield: 2.7 g; 14.5 mmol (83 %), **^{1}H-NMR** (500 MHz, $CDCl_3$): δ / ppm = 3.82 – 3.71 (m, 1H, Si-*H*), 1.58 – 1.42 (m, 2H, -C*H*-), 1.32 – 1.15 (m, 4H, -CH_2-), 0.94 – 0.85 (m, 12H, -CH_3), 0.66 – 0.49 (m, 4H, Si-CH_2-), 0.05 (d, $^3J_{H,H}$ = 3.7 Hz, 3H, Si-CH_3).**^{29}Si-NMR** (100 MHz, $CDCl_3$): δ / ppm = -8.59 (s, *Si*).

Methylbis(4-methylpentyl)silane) H2i6: Synthesized from 1-bromo-4-methylpentane (2.5 aq., 4.3 mL, 29.4 mmol), dichloromethylsilane (1.2 mL, 11.8 mmol) purification by column chromatography (eluent: *n*-hexane); colorless liquid, $C_{13}H_{30}Si$ M = 214.46 g/mol, yield: 0.6 g; 2.8 mmol (24 %), **^{1}H-NMR** (400 MHz, $CDCl_3$): δ / ppm = 3.83 – 3.70 (m, 1H, Si-*H*), 1.60 – 1.45 (m, 2H, -C*H*-),1.40 – 1.30 (m, 4H, -CH_2-), 1.30 – 1.11 (m, 4H, -CH_2-), 0.94 – 0.85 (m, 12H, -CH_3),0.65 – 0.48 (m, 4H, Si-CH_2-), 0.04 (d, $^3J_{H,H}$ = 3.7 Hz, 3H, Si-CH_3). **^{29}Si-NMR** (100 MHz, $CDCl_3$): δ / ppm = -9.91 (s, *Si*).

Methylbis(3,3-dimethylbutyl)silane H2t6: Synthesized from 1-bromo-3,3-dimethylsilane (2.5 aq., 4.1 mL, 28.8 mmol), dichloromethylsilane (1.2 mL, 11.5 mmol) purification by column chromatography (eluent: *n*-hexane); colorless liquid, $C_{13}H_{30}Si$ M = 214.46 g/mol, yield: 1.0 g; 4.7 mmol (41 %), **^{1}H-NMR** (400 MHz, $CDCl_3$): δ / ppm = 3.79 – 3.69 (m, 1H, Si-*H*), 1.24 – 1.13 (m, 4H, -CH_2-), 0.86 (s, 18H, -CH_3), 0.61 – 0.45 (m, 4H, Si-CH_2-), 0.04 (d, $^3J_{H,H}$ = 3.7 Hz, 3H, Si-CH_3). **^{29}Si-NMR** (80 MHz, $CDCl_3$): δ / ppm = -6.68 (s, *Si*).

Methylbis(2-cyclohexylethyl)silane H2c8: Synthesized from (2-Bromoethyl)cyclohexane (2.6 aq., 4.1 mL, 25.9 mmol), dichloromethylsilane (1.3 mL, 10.0 mmol) purification by column chromatography (eluent: *n*-hexane); colorless liquid, $C_{17}H_{34}Si$ M = 266.54 g/mol, yield: 1.1 g; 4.3 mmol (43 %), **^{1}H-NMR** (400 MHz, $CDCl_3$): δ / ppm = 3.80 – 3.68 (m, 1H, Si-*H*), 1.83 – 1.57 (m, 12H, -CH_2-),1.34 – 1.05 (m, 14H, -CH_2-, -C*H*-), 0.70 – 0.46 (m, 4H, Si-CH_2-), 0.03 (d, $^3J_{H,H}$ = 3.7 Hz, 3H,Si-CH_3). **^{29}Si-NMR** (80 MHz, $CDCl_3$): δ / ppm = -8.52 (s, *Si*).

Tricyclobutylsilane H3c4: Synthesized from bromocyclobutane (4.0 aq., 7.0 mL, 70.4 mmol), trichlorosilane (1.8 mL, 17.6 mmol) purification by column chromatography (eluent: *n*-hexane); colorless liquid, $C_{12}H_{22}Si$ M = 194.39 g/mol, yield: 2.8 g; 14.1 mmol (80 %), **^{1}H-NMR** (400 MHz, $CDCl_3$): δ / ppm = 3.78 – 3.70 (m, 1H, Si-*H*), 2.23 – 2.06 (m, 9H, -CH_2-),2.06 – 1.88 (m, 12H, -CH_2-, Si-C*H*-). **^{29}Si-NMR** (80 MHz, $CDCl_3$): δ / ppm = 1.08 (s, *Si*).

Tris(3-methylbutyl)silane H3i5:[S5] Synthesized from 1-bromo-3-methylbutane (3.5 aq., 25.8 mL, 51.7 mmol), trichlorosilane (25.84 mL, 51.7 mmol) purification by column chromatography (eluent: *n*-hexane); colorless liquid, $C_{15}H_{34}Si$ M = 242.52 g/mol, yield: 3.1 g; 13.0 mmol (88 %), **^{1}H-NMR** (400 MHz, $CDCl_3$): δ / ppm = 3.70 – 3.62 (m, 1H, Si-*H*), 1.59 – 1.37 (m, 3H, -C*H*-), 1.31 – 1.15 (m, 6H, -CH_2-), 0.88 (d, $^{3}J_{H,H}$ = 6.6 Hz, 18H, -CH_3), 0.63 – 0.52 (m, 6H, Si-CH_2-). **^{29}Si-NMR** (80 MHz, $CDCl_3$): δ / ppm = -4.60 (s, *Si*).

Tris(3,3-dimethylbutyl)silane H3t6:[S5] Synthesized from 1-Bromo-3,3-dimethylbutane (3.5 aq., 8.7 mL, 57.6 mmol), trichlorosilane (1.5 mL, 14.7 mmol) purification by column chromatography (eluent: *n*-hexane); colorless liquid, $C_{18}H_{40}Si$ M = 284.60 g/mol, yield: 3.1 g; 10.80 mmol (75 %), **^{1}H-NMR** (400 MHz, $CDCl_3$): δ / ppm = 3.69 – 3.60 (m, 1H, Si-*H*), 1.29 – 1.12 (m, 6H, -CH_2-), 0.87 (s, 27H, -CH_3), 0.60 – 0.48 (m, 6H, Si-CH_2-). **^{29}Si-NMR** (80 MHz, $CDCl_3$): δ / ppm = -1.95 (s, *Si*).

3.2.2 Synthesis of (4-bromobutyl)dialkylmethylsilanes Si2 and of 4-bromobutyltrialkylsilanes Br3 [S8]

In a two-neck flask under an inert gas atmosphere, 4-bromobut-1-ene (1.5 eq) is dissolved in diethyl ether (abs.) (9 mL/mmol), and Karstedt catalyst (3 mol%) is slowly added dropwise. The solution is stirred for 10 minutes at room temperature. Subsequently, the appropriate trialkylsilane **H2** or **H3** (1.0 eq) is added dropwise. The reaction mixture is stirred for 6 hours at 60 °C. The solvent was then distilled off under reduced pressure (40 °C, 850 mbar) using a rotary evaporator. The crude product was purified by column chromatography (eluent: *n*-hexane).

(4-Bromobutyl)dicyclobutylmethylsilane Br2c4: Synthesized from **dicyclobutyl(methyl)silane H2c4** (0.7 g, 4.5 mmol), 4-bromobut-1-ene (0.9 g, 0.7 mL, 6.8 mmol), Karstedt-cat. (0.6 mL) in anhydrous Et_2O; purification by column chromatography (eluent: *n*-hexane), colorless liquid $C_{13}H_{25}SiBr$ M = 289.33 g/mol, yield: 0.6 g, 1.9 mmol, (43 %), **^{1}H-NMR** (500 MHz, $CDCl_3$): δ / ppm = 3.41 (t, $^{3}J_{H,H}$ = 6.8 Hz, 2H, Br-CH_2-), 2.22 – 2.10 (m, 2H, -CH_2-) 2.10 – 1.99 (m, 4H, -CH_2-), 1.99 – 1.88 (m, 8H, -CH_2-, Si-C*H*-), 1.88 – 1.82 (m, 2H, -CH_2-), 1.48 – 1.37 (m, 2H, -CH_2-), 0.56 – 0.46 (m, 2H, Si-CH_2-), -0.01 (s, 3H, Si-CH_3). **^{29}Si-NMR** (100 MHz, $CDCl_3$): δ / ppm = 0.26 (s, *Si*).

(4-Bromobutyl)bis(3-methylbutyl)methylsilane Br2i5: Synthesized from **methylbis(3-methylbutyl)silane H2i5** (2.7 g, 14.5 mmol), 4-bromobut-1-ene (3.1 g, 2.3 mL, 21.8 mmol), Karstedt-cat. (0.2 mL) in anhydrous Et_2O; purification by column chromatography (eluent: *n*-hexane), colorless liquid $C_{15}H_{33}SiBr$ M = 321.41 g/mol, yield: 3.4 g, 10.5 mmol, (73 %), **^{1}H-NMR** (400 MHz, $CDCl_3$): δ / ppm = 3.42 (t, $^{3}J_{H,H}$ = 6.8 Hz, 2H, Br-CH_2-), 1.94 – 1.82(m, 2H, -CH_2-), 1.51 – 1.37 (m, 4H, -CH_2-, -C*H*-), 1.23 – 1.09 (m, 4H, -CH_2-), 0.95 – 0.81(m, 12H, -CH_3), 0.58 – 0.40 (m, 6H, Si-CH_2-), -0.06 (s, 3H, Si-CH_3). **^{29}Si-NMR** (80 MHz, $CDCl_3$): δ / ppm = 3.76 (s, *Si*).

(4-Bromobutyl)bis(4-methylpentyl)methylsilane Br2i6: Synthesized from **methylbis(4-methylpentyl)silane) H2i6** (0.6 g, 2.8 mmol), 4-bromobut-1-ene (0.7 g, 0.5 mL, 4.3 mmol), Karstedt-cat. (0.1 mL) in anhydrous Et_2O; purification by column chromatography (eluent: *n*-hexane), colorless liquid $C_{17}H_{37}SiBr$ M = 349.47 g/mol, yield: 0.5 g, 1.6 mmol, (55 %), **^{1}H-NMR** (400 MHz, $CDCl_3$): δ / ppm =3.42 (t, $^{3}J_{H,H}$ = 6.8 Hz, 2H, Br-CH_2-), 1.96 – 1.80 (m, 2H, -CH_2-), 1.64 – 1.48 (m, 2H, -C*H*-), 1.38 – 1.24

(m, 10H, -C*H$_2$*-), 0.92 – 0.79 (m, 12H, -C*H$_3$*), 0.58 – 0.43 (m, 6H, Si-C*H$_2$*-), -0.05 (s, 3H, Si-C*H$_3$*). **^{29}Si-NMR** (X00 MHz, $CDCl_3$): δ / ppm = 3.00 (s, *Si*).

(4-Bromobutyl)bis(3,3-dimethylbutyl)methylsilane Br2t6: Synthesized from **methylbis(3,3-dimethylbutyl)silane H2t6** (1.0 g, 4.6 mmol), 4-bromobut-1-ene (0.9 g, 0.7 mL, 6.9 mmol), Karstedt-cat. (0.1 mL) in anhydrous Et_2O; purification by column chromatography (eluent: *n*-hexane), colorless liquid $C_{17}H_{37}SiBr$ M = 349.47 g/mol, yield: 0.8 g, 2.4 mmol, (51 %), **^{1}H-NMR** (400 MHz, $CDCl_3$): δ / ppm = 3.43 (t, $^3J_{H,H}$ = 6.8 Hz, 2H, Br-C*H$_2$*-), 1.95 – 1.82 (m, 2H, -C*H$_2$*-), 1.49 – 1.39 (m, 2H, -C*H$_2$*-), 1.24 – 1.08 (m, 4H, -C*H$_2$*-), 0.86 (s, 18H, -C*H$_3$*), 0.47 – 0.37 (m, 6H, Si-C*H$_2$*-), -0.06 (s, 3H, Si-C*H$_3$*). **^{9}Si-NMR** (80 MHz, $CDCl_3$): δ / ppm = 4.72 (s, *Si*).

(4-Bromobutyl)bis(2-cyclohexylethyl)methylsilane Br2c8: Synthesized from **methylbis(2-cyclohexylethyl)silane H2c8** (1.1 g, 4.3 mmol), 4-bromobut-1-ene (0.9 g, 0.7 mL, 6.4 mmol), Karstedt-cat. (0.1 mL) in anhydrous Et_2O; purification by column chromatography (eluent: *n*-hexane), colorless liquid $C_{21}H_{41}SiBr$ M = 401.54 g/mol, yield: 0.4 g, 1.0 mmol, (23 %), **^{1}H-NMR** (400 MHz, $CDCl_3$): δ / ppm = 3.42 (t, $^3J_{H,H}$ = 6.8 Hz, 2H, Br-C*H$_2$*-), 1.95 – 1.80 (m, 2H, -C*H$_2$*-), 1.80 – 1.57 (m, 10H, -C*H$_2$*-), 1.48 – 1.37 (m, 2H, -C*H$_2$*-), 1.36 – 1.00 (m, 12H, -C*H$_2$*-, -C*H*-), 0.94 – 0.74 (m, 4H, -C*H$_2$*-), 0.56 – 0.43 (m, 6H, Si-C*H$_2$*-), -0.08 (s, 3H, Si-C*H$_3$*). **^{29}Si-NMR** (80 MHz, $CDCl_3$): δ / ppm = 3.83 (s, *Si*).

(4-Bromobutyl)tricyclobutylsilane Br3c4: Synthesized from **tricyclobutylsilane H3c4** (1.5 g, 7.7 mmol), 4-bromobut-1-ene (1.6 g, 1.2 mL, 11.6 mmol), Karstedt-cat. (0.1 mL) in anhydrous Et_2O; purification by column chromatography (eluent: *n*-hexane), colorless liquid $C_{16}H_{29}SiBr$ M = 329.39 g/mol, yield: 0.7 g, 2.1 mmol, (28 %), **^{1}H-NMR** (400 MHz, $CDCl_3$): δ / ppm = 3.43 (t, $^3J_{H,H}$ = 6.8 Hz, 2H, Br-C*H$_2$*-), 2.25 – 2.12 (m, 3H, Si-C*H*-), 2.11 – 1.91 (m, 18H, -C*H$_2$*-), 1.90 – 1.82 (m, 2H, -C*H$_2$*-), 1.49 – 1.39 (m, 2H, -C*H$_2$*-), 0.65 – 0.55 (m, 2H, Si-C*H$_2$*-). **^{29}Si-NMR** (80 MHz, $CDCl_3$): δ / ppm = -3.73 (s, *Si*).

(4-Bromobutyl)tris(3-methylbutyl)silane Br3i5: Synthesized from **tris(3-methylbutyl)silane H3i5** (3.1 g, 13.0 mmol), 4-bromobut-1-ene (2.7 g, 2.0 mL, 19.4 mmol), Karstedt-cat. (0.2 mL) in anhydrous Et_2O; purification by column chromatography (eluent: *n*-hexane), colorless liquid $C_{19}H_{41}SiBr$ M = 377.52 g/mol, yield: 3.4 g, 9.0 mmol, (70 %), **^{1}H-NMR** (400 MHz, $CDCl_3$): δ / ppm = 3.43 (t, $^3J_{H,H}$ = 6.8 Hz, 2H, Br-C*H$_2$*-), 1.90 – 1.82 (m, 2H, -C*H$_2$*-), 1.50 – 1.36 (m, 3H, -C*H*-), 1.25 – 1.09 (m, 8H, -C*H$_2$*-), 0.92 – 0.79 (m, 18H, -C*H$_3$*), 0.54 – 0.43 (m, 8H, Si-C*H$_2$*-). **^{29}Si-NMR** (80 MHz, $CDCl_3$): δ / ppm = 5.60 (s, *Si*).

(4-Bromobutyl)tris(3,3-dimethylbutyl)silane Br3t6: Synthesized from **tris(3,3-dimethylbutyl)silane H3t6** (3.0 g, 10.6 mmol), 4-bromobut-1-ene (2.2 g, 1.7 mL, 16.0 mmol), Karstedt-cat. (0.2 mL) in anhydrous Et_2O; purification by column chromatography (eluent: *n*-hexane), colorless liquid $C_{22}H_{47}SiBr$ M = 419.60 g/mol, yield: 0.9 g, 2.1 mmol, (20 %), **^{1}H-NMR** (400 MHz, $CDCl_3$): δ / ppm = 3.44 (t, $^3J_{H,H}$ = 6.8 Hz, 2H, Br-C*H$_2$*-), 1.92 – 1.83 (m, 2H, -C*H$_2$*-), 1.50 – 1.40 (m, 2H, -C*H$_2$*-), 1.23 – 1.09 (m, 6H, -C*H$_2$*-), 0.86 (s, 27H, -C*H$_3$*), 0.57 – 0.38 (m, 8H, Si-C*H$_2$*-). **^{29}Si-NMR** (80 MHz, $CDCl_3$): δ / ppm = 5.60 (s, *Si*).

3.2.3 Synthesis of the acetonides 2A and 3A [S9]

In a single-necked round-bottom flask fitted with a reflux condenser, the corresponding phenol (1.0 eq), (4-bromobutyl)di-alkylmethylsilanes **Br2** or of (4-bromobutyl)trialkylsilanes **Bri3** (1.1 eq.), K_2CO_3 (2 eq.), and Bu_4NI (tip of a spatula) is added to DMF (abs.) (51 ml/mmol phenol) and stirred for 12 hours at 120 °C. The reaction mixture is cooled to room temperature, and 20 mL of water is added. The reaction mixture was extracted with diethyl ether (3 × 50 mL). The combined organic phases were washed with saturated lithium chloride solution (50 mL), distilled water (50 mL), and saturated sodium chloride solution (50 mL), and then dried over sodium sulfate. The solvent is distilled off on a rotary evaporator at 40 °C and 850 mbar. The product is purified by column chromatography.

2'-[4-(Dicyclobutylmethylsilyl)-*n*-butlyoxy]-4,4''-bis[(2,2-dimethyl-1,3-dioxolane-4-yl)methoxy]-*p*-terphenyl (2c4A): Synthesized from **1** (400 mg, 0.8 mmol) and **Br2c4** (341 mg, 1.2 mmol) with K_2CO_3 (218 mg, 1.6 mmol), TBAI (0.2 g, 0.05 mmol) in DMF (51 mL); purified by column chromatography (eluent: $CHCl_3$); colorless liquid, $C_{43}H_{58}O_7Si$ M = 715.00 g/mol, yield: 550 mg, 0.8 mmol, (98 %), **^{1}H-NMR** (400 MHz, $CDCl_3$): δ / ppm = 7.56 (d, $^3J_{H,H}$ = 8.8 Hz, 2H, Aryl-*H*), 7.53 (d, $^3J_{H,H}$ = 8.8 Hz, 2H, Aryl-*H*), 7.35 (d, $^3J_{H,H}$ = 7.9 Hz, 1H, Aryl-*H*), 7.18 (dd, $^3J_{H,H}$ = 7.9 Hz, $^4J_{H,H}$ = 1.4 Hz, 1H, Aryl-*H*), 7.13 (d, $^4J_{H,H}$ = 1.7 Hz, 1H, Aryl-*H*), 7.00 (d, $^3J_{H,H}$ = 8.8 Hz, 2H, Aryl-*H*), 6.93 (d, $^3J_{H,H}$ = 8.7 Hz, 2H, Aryl-*H*), 4.57 – 4.46 (m, 2H, -OC*H*-), 4.25 – 4.16 (m, 2H, -OC*H*$_2$-), 4.16 – 4.08 (m, 2H, -OC*H*$_2$-), 4.06 – 3.90 (m, 6H, -OC*H*$_2$-), 2.20 – 2.08 (m, 2H, -C*H*$_2$-), 2.09 – 1.98 (m, 4H, -C*H*$_2$-), 1.98 – 1.81 (m, 8H, -C*H*$_2$-, -C*H*-), 1.81 – 1.71 (m, 2H, -C*H*$_2$-), 1.49 (s, 6H, -C*H*$_3$), 1.46 – 1.35 (m, 8H, -C*H*$_3$, -C*H*$_2$-), 0.57 – 0.47 (m, 2H, Si-C*H*$_2$-), -0.02 (s, 3H, Si-C*H*$_3$). **^{29}Si-NMR** (80 MHz, $CDCl_3$): δ [ppm] = 0.22 (s, *Si*).

2'-{4-[Bis(3-methylbutyl)methylsilyl]-*n*-butlyoxy}-4,4''-bis[(2,2-dimethyl-1,3-dioxolane-4-yl)methoxy]-*p*-terphenyl (2i5A): Synthesized from **1** (400 mg, 0.8 mmol) and **Br2i5** (305 mg, 1.0 mmol) with K_2CO_3 (218 mg, 1.6 mmol), TBAI (0.2 g, 0.05 mmol) in DMF (51 mL); purified by column chromatography (eluent: $CHCl_3$); colorless liquid, $C_{45}H_{66}O_7Si$ M = 747.09 g/mol, yield: 530 mg, 0.7 mmol, (90 %), **^{1}H-NMR** (400 MHz, $CDCl_3$): δ / ppm = 7.56 (d, $^3J_{H,H}$ = 8.7 Hz, 2H, Aryl-*H*), 7.53 (d, $^3J_{H,H}$ = 8.8 Hz, 2H, Aryl-*H*), 7.36 (d, $^3J_{H,H}$ = 7.9 Hz, 1H, Aryl-*H*), 7.18 (dd, $^3J_{H,H}$ = 7.9 Hz, $^4J_{H,H}$ = 1.6 Hz, 1H, Aryl-*H*), 7.13 (d, $^4J_{H,H}$ = 1.7 Hz, 1H, Aryl-*H*), 7.00 (d, $^3J_{H,H}$ = 8.8 Hz, 2H, Aryl-*H*), 6.93 (d, $^3J_{H,H}$ = 8.8 Hz, 2H, Aryl-*H*), 4.57 – 4.46 (m, 2H, -OC*H*-), 4.23 – 4.17 (m, 2H, -OC*H*$_2$-), 4.16 – 4.08 (m, 2H, -OC*H*$_2$-), 4.07 – 3.90 (m, 6H, -OC*H*$_2$-), 1.84 – 1.71 (m, 2H, -C*H*$_2$-), 1.53 – 1.47 (m, 6H, -C*H*$_3$), 1.46 – 1.36 (m, 10H, -C*H*$_3$, -C*H*-, -C*H*$_2$-), 1.21 – 1.08 (m, 4H, -C*H*$_2$-), 0.92 – 0.82 (m, 12H, -C*H*$_3$), 0.58 – 0.41 (m, 6H, Si-C*H*$_2$-), -0.07 (s, 3H, Si-C*H*$_3$). **^{29}Si-NMR** (80 MHz, $CDCl_3$): δ [ppm] = 3.67 (s, *Si*).

2'-{4-[Bis(4-methylpentyl)methylsilyl]-*n*-butlyoxy}-4,4''-bis[(2,2-dimethyl-1,3-dioxolane-4-yl)methoxy]-*p*-terphenyl (2i6A): Synthesized from **1** (400 mg, 0.8 mmol) and **Br2i6** (414 mg, 1.2 mmol) with K_2CO_3 (218 mg, 1.6 mmol), TBAI (0.2 g, 0.05 mmol) in DMF (51 mL); purified by column chromatography (eluent: $CHCl_3$); colorless liquid, $C_{47}H_{70}O_7Si$ M = 775.14 g/mol, yield: 510 mg, 0.7 mmol, (84 %), **^{1}H-NMR** (400 MHz, $CDCl_3$): δ / ppm = 7.56 (d, $^3J_{H,H}$ = 8.7 Hz, 2H, Aryl-*H*), 7.53 (d, $^3J_{H,H}$ = 8.8 Hz, 2H, Aryl-*H*), 7.35 (d, $^3J_{H,H}$ = 7.8 Hz, 1H, Aryl-*H*), 7.18 (dd, $^3J_{H,H}$ = 7.9 Hz, $^4J_{H,H}$ = 1.7 Hz, 1H, Aryl-*H*), 7.13 (d, $^4J_{H,H}$ = 1.7 Hz, 1H, Aryl-*H*), 7.01 (d, $^3J_{H,H}$ = 8.7 Hz, 2H, Aryl-*H*), 6.96 (d, $^3J_{H,H}$ = 8.8 Hz, 2H, Aryl-*H*), 4.57 – 4.47 (m, 2H, -OC*H*-), 4.23 – 4.17 (m, 2H, -OC*H*$_2$-), 4.16 – 4.09 (m, 2H, -OC*H*$_2$-), 4.07 – 3.90 (m, 6H, -OC*H*$_2$-), 1.86 – 1.71 (m, 2H, -C*H*$_2$-), 1.64 – 1.51 (m, 2H, -C*H*-), 1.49 (s, 6H, -C*H*$_3$), 1.47 – 1.38 (m, 8H, -C*H*$_3$, -C*H*$_2$-), 1.38 – 1.23 (m, 4H, -C*H*$_2$-), 1.23 – 1.14 (m, 4H, -C*H*$_2$-), 0.96 – 0.78 (m, 12H, -C*H*$_3$), 0.57 – 0.38 (m, 6H, Si-C*H*$_2$-), -0.06 (s, 3H, Si-C*H*$_3$). **^{29}Si-NMR** (80 MHz, $CDCl_3$): δ [ppm] = 2.39 (s, *Si*).

2'-{4-[Bis(3,3-dimethylbutyl)methylsilyl]-*n*-butlyoxy}-4,4''-bis[(2,2-dimethyl-1,3-dioxolane-4-yl)methoxy]-*p*-terphenyl (2t6A): Synthesized from **1** (400 mg, 0.8 mmol) and **Br2t6** (414 mg, 1.2 mmol) with K_2CO_3 (218 mg, 1.6 mmol), TBAI (0.2 g, 0.05 mmol) in DMF (51 mL); purified by column chromatography (eluent: $CHCl_3$); colorless liquid, $C_{47}H_{70}O_7Si$ M = 775.14 g/mol, yield: 590 mg, 0.8 mmol, (96 %), **^{1}H-NMR** (400 MHz, $CDCl_3$): δ / ppm = 7.56 (d, $^3J_{H,H}$ = 8.7 Hz, 2H, Aryl-*H*), 7.53 (d, $^3J_{H,H}$ = 8.7 Hz, 2H, Aryl-*H*), 7.35 (d, $^3J_{H,H}$ = 7.8 Hz, 1H, Aryl-*H*), 7.18 (dd, $^3J_{H,H}$ = 7.8 Hz, $^4J_{H,H}$ = 1.6 Hz, 1H, Aryl-*H*), 7.13 (d, $^4J_{H,H}$ = 1.7 Hz, 1H, Aryl-*H*), 7.00 (d, $^3J_{H,H}$ = 8.8 Hz, 2H, Aryl-*H*), 6.96 (d, $^3J_{H,H}$ = 8.8 Hz, 2H, Aryl-*H*), 4.57 – 4.47 (m, 2H, -OC*H*-), 4.24 – 4.17 (m, 2H, -OC*H*$_2$-), 4.16 – 4.09 (m, 2H, -OC*H*$_2$-), 4.08 – 3.90 (m, 6H, -OC*H*$_2$-), 1.85 – 1.73 (m, 2H, -C*H*$_2$-), 1.49 (s, 6H, -C*H*$_3$), 1.43 (s, 6H, -C*H*$_3$), 1.22 – 1.09 (m, 6H, -C*H*$_2$-), 0.85 (s, 18H, -C*H*$_3$), 0.49 – 0.37 (m, 6H, Si-C*H*$_2$-), -0.07 (s, 3H, Si-C*H*$_3$). **^{29}Si-NMR** (80 MHz, $CDCl_3$): δ [ppm] = 8.51 (s, *Si*).

2'-{4-[Bis(2-cyclohexylethyl)methylsilyl]-*n*-butlyoxy}-4,4''-bis[(2,2-dimethyl-1,3-dioxolane-4-yl)methoxy]-*p*-terphenyl (2c8A): Synthesized from **1** (400 mg, 0.8 mmol) and **Br2c8** (328 mg, 0.8

mmol) with K_2CO_3 (218 mg, 1.6 mmol), TBAI (0.2 g, 0.05 mmol) in DMF (51 mL); purified by column chromatography (eluent: $CHCl_3$); colorless liquid, $C_{51}H_{74}O_7Si$ M = 827.22 g/mol, yield: 560 mg, 0.7 mmol, (86 %), **^{1}H-NMR** (400 MHz, $CDCl_3$): δ / ppm = 7.56 (d, $^3J_{H,H}$ = 8.8 Hz, 2H, Aryl-*H*), 7.53 (d, $^3J_{H,H}$ = 8.7 Hz, 2H, Aryl-*H*), 7.35 (d, $^3J_{H,H}$ = 7.8 Hz, 1H, Aryl-*H*), 7.18 (dd, $^3J_{H,H}$ = 7.8 Hz, $^4J_{H,H}$ = 1.4 Hz, 1H, Aryl-*H*), 7.13 (d, $^4J_{H,H}$ = 1.7 Hz, 1H, Aryl-*H*), 7.00 (d, $^3J_{H,H}$ = 8.7 Hz, 2H, Aryl-*H*), 6.96 (d, $^3J_{H,H}$ = 8.8 Hz, 2H, Aryl-*H*), 4.57 – 4.46 (m, 2H, -OC*H*-), 4.24 – 4.17 (m, 2H, -OCH_2-), 4.15 – 4.09 (m, 2H, -OCH_2-), 4.08 – 3.91 (m, 6H, -OCH_2-), 1.90 – 1.56 (m, 11H, -CH_2-), 1.49 (s, 6H, -CH_3), 1.46 – 1.38 (m, 8H, -CH_3, -C*H*-), 1.30 – 0.97 (m, 11H, -CH_2-), 0.91 – 0.67 (m, 6H, -CH_2-), 0.56 – 0.43 (m, 6H, Si-CH_2-), -0.08 (s, 3H, Si-CH_3). **^{29}Si-NMR** (80 MHz, $CDCl_3$): δ [ppm] = 3.75 (s, *Si*).

2'-[4-(Tricyclobutylsilyl)-*n*-butlyoxy]-4,4''-bis[(2,2-dimethyl-1,3-dioxolane-4-yl)methoxy]-*p*-terphenyl (3c4A): Synthesized from **1** (400 mg, 0.8 mmol) and **Br3c4** (390 mg, 1.2 mmol) with K_2CO_3 (218 mg, 1.6 mmol), TBAI (0.2 g, 0.05 mmol) in DMF (51 mL); purified by column chromatography (eluent: $CHCl_3$); colorless liquid, $C_{46}H_{62}O_7Si$ M = 755.07 g/mol, yield: 530 mg, 0.7 mmol, (89 %), **^{1}H-NMR** (400 MHz, $CDCl_3$): δ / ppm = 7.60 – 7.49 (m, 4H, Aryl-*H*), 7.36 (d, $^3J_{H,H}$ = 7.8 Hz, 1H, Aryl-*H*), 7.18 (dd, $^3J_{H,H}$ = 7.8 Hz, $^4J_{H,H}$ = 1.7 Hz, 1H, Aryl-*H*), 7.13 (d, $^4J_{H,H}$ = 1.7 Hz 1H, Aryl-*H*), 7.00 (d, $^3J_{H,H}$ = 8.8 Hz, 2H, Aryl-*H*), 6.97 (d, $^3J_{H,H}$ = 8.7 Hz, 2H, Aryl-*H*), 4.58 – 4.43 (m, 2H, -OC*H*-), 4.24 – 4.17 (m, 2H, -OCH_2-), 4.16 – 4.08 (m, 2H, -OCH_2-), 4.08 – 3.97 (m, 4H, -OCH_2-), 3.97 – 3.90 (m, 2H, -OCH_2-), 2.22 – 2.10 (m, 3H, Si-C*H*-), 2.07 – 1.86 (m, 18H, -CH_2-), 1.84 – 1.72 (m, 2H, -CH_2-), 1.49 (s, 6H, -CH_3), 1.47 – 1.37 (m, 8H, -CH_3, -CH_2-), 0.68 – 0.55 (m, 2H, Si-CH_2-). **^{29}Si-NMR** (80 MHz, $CDCl_3$): δ [ppm] = -3.75 (s, *Si*).

2'-{4-[Tris(3-methylbutyl)silyl]-*n*-butlyoxy}-4,4''-bis[(2,2-dimethyl-1,3-dioxolane-4-yl)methoxy]-*p*-terphenyl (3i5A): Synthesized from **1** (400 mg, 0.8 mmol) and **Br3i5** (533 mg, 1.6 mmol) with K_2CO_3 (218 mg, 1.6 mmol), TBAI (0.2 g, 0.05 mmol) in DMF (51 mL); purified by column chromatography (eluent: $CHCl_3$); colorless liquid, $C_{49}H_{74}O_7Si$ M = 803.19 g/mol, yield: 610 mg, 0.8 mmol, (98 %), **^{1}H-NMR** (400 MHz, $CDCl_3$): δ / ppm = 7.60 – 7.50 (m, 4H, Aryl-*H*), 7.36 (d, $^3J_{H,H}$ = 7.9 Hz, 1H, Aryl-*H*), 7.18 (dd, $^3J_{H,H}$ = 7.8 Hz, $^4J_{H,H}$ = 1.7 Hz, 1H, Aryl-*H*), 7.14 (d, $^4J_{H,H}$ = 1.7 Hz, 1H, Aryl-*H*), 7.00 (d, $^3J_{H,H}$ = 8.8 Hz, 2H, Aryl-*H*), 6.96 (d, $^3J_{H,H}$ = 8.8 Hz, 2H, Aryl-*H*), 4.55 – 4.46 (m, 2H, -OC*H*-), 4.23 – 4.17 (m, 2H, -OCH_2-), 4.16 – 4.08 (m, 2H, -OCH_2-), 4.07 – 3.90 (m, 6H, -OCH_2-), 1.85 – 1.73 (m, 2H, -CH_2-), 1.49 (s, 6H, -CH_3), 1.46 – 1.37 (m, 9H, -CH_3, -C*H*-), 1.24 – 1.07 (m, 8H, -CH_2-), 0.97 – 0.77 (m, 18H, -CH_3), 0.64 – 0.43 (m, 8H, Si-CH_2-). **^{29}Si-NMR** (80 MHz, $CDCl_3$): δ [ppm] = 4.11 (s, *Si*).

2'-{4-[Tris(3,3-dimethylbutyl)silyl]-*n*-butlyoxy}-4,4''-bis[(2,2-dimethyl-1,3-dioxolane-4-yl)methoxy]-*p*-terphenyl (3t6A): Synthesized from **1** (400 mg, 0.8 mmol) and **Br3t6** (700 mg, 1.7 mmol) with K_2CO_3 (218 mg, 1.6 mmol), TBAI (0.2 g, 0.05 mmol) in DMF (51 mL); purified by column chromatography (eluent: $CHCl_3$); colorless liquid, $C_{52}H_{80}O_7Si$ M = 845.27 g/mol, yield: 590 mg, 0.7 mmol, (89 %), **^{1}H-NMR** (400 MHz, $CDCl_3$): δ / ppm = 7.61 – 7.47 (m, 4H, Aryl-*H*), 7.35 (d, $^3J_{H,H}$ = 7.8 Hz, 1H, Aryl-*H*), 7.18 (dd, $^3J_{H,H}$ = 7.9 Hz, $^4J_{H,H}$ = 1.6 Hz, 1H, Aryl-*H*), 7.14 (d, $^4J_{H,H}$ = 1.7 Hz, 1H, Aryl-*H*), 7.00 (d, $^3J_{H,H}$ = 8.7 Hz, 2H, Aryl-*H*), 6.97 (d, $^3J_{H,H}$ = 8.7 Hz, 2H, Aryl-*H*), 4.57 – 4.46 (m, 2H, -OC*H*-), 4.24 – 4.16 (m, 2H, -OCH_2-), 4.16 – 4.08 (m, 2H, -OCH_2-), 4.08 – 3.89 (m, 6H, -OCH_2-), 1.87 – 1.74(m, 2H, -CH_2-), 1.49 (s, 6H, -CH_3), 1.43 (s, 6H, -CH_3), 1.18 – 1.06 (m, 8H, -CH_2-), 0.85 (s, 27H, -CH_3), 0.49 – 0.38 (m, 8H, Si-CH_2-). **^{29}Si-NMR** (80 MHz, $CDCl_3$): δ [ppm] = 5.45 (s, *Si*).

3.3. Synthesis of compounds 2 and 3 [S10]

The acetonide **2A** or **3A** (1.0 eq) and PPTS (2.0 eq) are dissolved in a mixture of tetrahydrofuran (37 mL/mmol acetonide) and methanol (37 mL/mmol acetonide) and stirred for 24 hours at 50 °C. The progress of the reaction is monitored by thin-layer chromatography. After the reaction is complete, the solvent is distilled off under reduced pressure (40 °C, 400 mbar) using a rotary evaporator. The

residue is purified by column chromatography (eluent: $CHCl_3$ / MeOH, 9:1) and then recrystallized from methanol.

2'-[4-(Dicyclobutylmethylsilyl)-*n*-but-1-yloxy]-4,4´´-bis(2,3-dihydroxypropyloxy)-*p*-terphenyl 2c4: Synthesized from **2c4A** (550 mg, 0.8 mmol), PPTS (387 mg, 1.6 mmol) in MeOH (25 mL) and THF (25 mL), yield: 303 mg, 0.5 mmol, (62 %), colorless solid; **^{1}H-NMR** (500 MHz, Pyridine-d_5): δ / ppm = 7.77 (dd, $^3J_{H,H}$ = 8.7 Hz, $^4J_{H,H}$ = 3.0 Hz, 4H, Aryl-H), 7.53 (d, $^3J_{H,H}$ = 7.7 Hz, 1H, Aryl-*H*), 7.43 (d, $^4J_{H,H}$ = 1.7 Hz, 1H, Aryl-*H*), 7.38 (dd, $^3J_{H,H}$ = 7.9 Hz, $^4J_{H,H}$ = 1.7 Hz, 1H, Aryl-*H*), 7.27 – 7.21 (m, 4H, Aryl-*H*), 6.03 (broad, 4H, -O*H*), 4.65 – 4.54 (m, 4H, -OC*H$_2$*-), 4.52 – 4.45 (m, 2H, -OC*H*-), 4.30 – 4.19 (m, 4H, -OC*H$_2$*-), 4.09 (t, $^3J_{H,H}$ = 6.2 Hz, 2H, -OC*H$_2$*-), 2.18 – 2.08 (m, 2H, -C*H$_2$*-), 2.07 – 1.98 (m, 4H, -C*H$_2$*-), 1.97 – 1.81 (m, 8H, -C*H$_2$*-), 1.81 – 1.74 (m, 2H, -C*H$_2$*-), 1.57 – 1.45 (m, 2H, Si-C*H*-), 0.59 – 0.50 (m, 2H, Si-C*H$_2$*-), 0.02 (s, 3H, Si-C*H$_3$*). **^{13}C-NMR** (126 MHz, Pyridine-d_5): δ / ppm = 160.87, 160.20, 158.21 (*Ar*-O), 142.56, 135.02, 132.62 (*Ar$_{quart}$*), 132.57, 132.38 (*Ar*-H), 130.52 (*Ar$_{quart}$*), 129.75, 120.77, 116.79, 115.89, 112.64 (*Aryl*-H), 72.78 (-O*C*H-), 72.33, 72.22, 69.55, 65.69, 65.62 (-O*C*H$_2$-), 35.00, 25.37, 24.99, 22.41 (-*C*H$_2$-), 22.00 (Si-*C*H-), 12.76 (Si-*C*H$_2$-), -7.68 (Si-*C*H$_3$). **^{29}Si-NMR** (100 MHz, Pyridine-d_5): δ / ppm = 1.67 (s, *Si*). **HR-MS** (m/z): [M]+Li$^+$ calcd. For $C_{37}H_{50}O_7SiLi$, 641.3481, found: 641.3514.

2'-{4-[Bis(3-methylbutyl)methylsilyl]-*n*-but-1-yloxy}-4,4´´-bis(2,3-dihydroxypropyloxy)-*p*-terphenyl 2i5: Synthesized from **2i5A** (530 mg, 0.7 mmol), PPTS (357 mg, 1.4 mmol) in MeOH (25 mL) and THF (25 mL), yield: 283 mg, 0.4 mmol, (59 %), colorless solid; **^{1}H-NMR** (400 MHz, Pyridine-d_5): δ / ppm = 7.84 – 7.73 (m, 4H, Aryl-*H*), 7.53 (d, $^3J_{H,H}$ = 7.8 Hz, 1H, Aryl-*H*), 7.44 (d, $^4J_{H,H}$ = 1.7 Hz, 1H, Aryl-*H*), 7.39 (dd, $^3J_{H,H}$ = 7.9 Hz, $^4J_{H,H}$ = 1.4 Hz, 1H, Aryl-*H*), 7.28 – 7.21 (m, 4H, Aryl-*H*), 6.38 (broad, 4H, -O*H*), 4.65 – 4.52 (m, 4H, -OC*H$_2$*-), 4.52 – 4.43 (m, 2H, -OC*H*-), 4.29 – 4.19 (m, 4H, -OC*H$_2$*-), 4.11 (t, $^3J_{H,H}$ = 6.2 Hz, 2H, -OC*H$_2$*-), 1.90 – 1.77 (m, 2H, -C*H$_2$*-), 1.64 – 1.52 (m, 2H, -C*H*-), 1.52 – 1.41 (m, 2H, -C*H$_2$*-), 1.28 – 1.18 (m, 4H, -C*H$_2$*-), 0.96 – 0.84 (m, 12H, -C*H$_3$*), 0.64 – 0.49 (m, 6H, Si-C*H$_2$*-), 0.04 (s, 3H, Si-C*H$_3$*). **^{13}C-NMR** (101 MHz, Pyridine-d_5): δ / ppm = 160.89, 160.21, 158.23 (*Ar*-O), 142.57, 135.04, 132.64 (*Ar$_{quart}$*), 132.60, 132.40 (*Ar*-H), 130.51 (*Ar$_{quart}$*), 129.77, 120.80, 116.81, 115.93, 112.66 (*Aryl*-H), 72.79 (-O*C*H-), 72.34, 72.21, 69.57, 65.70, 65.64 (-O*C*H$_2$-), 34.87, 34.52 (-*C*H$_2$-), 32.58 (-*C*H-), 23.62 (-*C*H$_3$), 22.23 (-*C*H$_2$-), 14.93, 12.53 (Si-*C*H$_2$-), -3.87 (-*C*H$_3$). **^{29}Si-NMR** (80 MHz, Pyridine-d_5): δ / ppm = 5.13 (s, *Si*). **HR-MS** (m/z): [M]+Li$^+$ calcd. For $C_{39}H_{58}O_7SiLi$, 673.4107, found: 673.4114.

2'-{4-[Bis(4-methylpentyl)methylsilyl]-*n*-but-1-yloxy}-4,4´´-bis(2,3-dihydroxypropyloxy)-*p*-terphenyl Si6: Synthesized from **Si6A** (510 mg, 0.7 mmol), PPTS (332 mg, 1.4 mmol) in MeOH (25 mL) and THF (25 mL), yield: 191 mg, 0.3 mmol, (42 %), colorless solid; **^{1}H-NMR** (400 MHz, Pyridine-d_5): δ / ppm = 7.81 – 7.74 (m, 4H, Aryl-*H*), 7.53 (d, $^3J_{H,H}$ = 7.9 Hz, 1H, Aryl-*H*), 7.44 (d, $^4J_{H,H}$ = 1.7 Hz, 1H, Aryl-*H*), 7.39 (dd, $^3J_{H,H}$ = 7.9 Hz, $^4J_{H,H}$ = 1.6 Hz, 1H, Aryl-*H*), 7.24 (dd, $^3J_{H,H}$ = 8.7 Hz, $^4J_{H,H}$ = 3.6 Hz, 1H, Aryl-*H*), 6.26 (broad, 4H, -O*H*), 4.65 – 4.52 (m, 4H, -OC*H$_2$*-), 4.52 – 4.44 (m, 2H, -OC*H*-), 4.30 – 4.19 (m, 4H, -OC*H$_2$*-), 4.12 (t, $^3J_{H,H}$ = 6.3 Hz, 2H, -OC*H$_2$*-), 1.91 – 1.77 (m, 2H, -C*H$_2$*-), 1.65 – 1.47 (m, 4H, -C*H*-, -C*H$_2$*-), 1.44 – 1.31 (m, 4H, -C*H$_2$*-), 1.29 – 1.18 (m, 4H, -C*H$_2$*-), 0.97 – 0.80 (m, 12H, -C*H$_3$*), 0.66 – 0.50 (m, 6H, Si-C*H$_2$*-), 0.06 (s, 3H, Si-C*H$_3$*). **^{13}C-NMR** (101 MHz, Pyridine-d_5): δ / ppm = 159.38, 158.70, 156.72 (*Ar*-O), 141.07, 133.53, 131.14 (*Ar$_{quart}$*), 131.09 (*Ar*-H), 130.89 (*Ar$_{quart}$*), 129.02, 128.26, 119.30, 115.30, 114.42, 111.16 (*Aryl*-H), 71.28 (-O*C*H-), 70.83, 70.70, 68.05, 64.19, 64.13 (-O*C*H$_2$-), 43.19, 33.33 (-*C*H$_2$-), 27.57 (-*C*H-), 22.52 (-*C*H$_3$), 21.73, 20.74 (-*C*H$_2$-), 13.94, 13.61 (Si-*C*H$_2$-), -5.16 (Si-*C*H$_3$). **^{29}Si-NMR** (80 MHz, Pyridine-d_5): δ / ppm = 2.88 (s, *Si*). **HR-MS** (m/z): [M]+Li$^+$ calcd. For $C_{41}H_{62}O_7SiLi$, 701.4420, found: 701.4460.

2'-{4-[Bis(3,3-dimethylbutyl)methylsilyl]-*n*-but-1-yloxy}-4,4´´-bis(2,3-dihydroxypropyloxy)-*p*-terphenyl 2t6: Synthesized from **2t6A** (590 mg, 0.8 mmol), PPTS (382 mg, 1.6 mmol) in MeOH (25 mL) and THF (25 mL), yield: 320 mg, 0.5 mmol, (61 %), colorless solid; **^{1}H-NMR** (400 MHz, Pyridine-d_5): δ / ppm = 7.83 – 7.72 (m, 4H, Aryl-*H*), 7.53 (d, $^3J_{H,H}$ = 7.9 Hz, 1H, Aryl-*H*), 7.43 (d, $^4J_{H,H}$ = 1.7 Hz, 1H, Aryl-*H*), 7.38 (dd, $^3J_{H,H}$ = 7.9 Hz, $^4J_{H,H}$ = 1.6 Hz, 1H, Aryl-*H*), 7.28 – 7.21 (m, 4H, Aryl-*H*), 5.92 (broad, 4H, -O*H*), 4.65 – 4.53 (m, 4H, -OC*H$_2$*-), 4.52 – 4.43 (m, 2H, -OC*H*-), 4.31 – 4.18 (m, 4H, -OC*H$_2$*-), 4.12 (t, $^3J_{H,H}$ = 6.3 Hz, 2H, -OC*H$_2$*-), 1.94 – 1.77 (m, 2H, -C*H$_2$*-), 1.65 – 1.49 (m, 2H, -C*H$_2$*-), 1.33 – 1.21 (m, 4H, -C*H$_2$*-), 0.89 (s, 18H, -C*H$_3$*), 0.67 – 0.58 (m, 2H, Si-C*H$_2$*-), 0.58 – 0.49 (m, 4H, Si-C*H$_2$*-), 0.06 (s, 3H, Si-C*H$_3$*). **^{13}C-NMR** (101 MHz, Pyridine-d_5): δ / ppm = 159.38, 158.70, 156.72 (*Ar*-O), 141.06, 133.53, 131.13 (*Ar$_{quart}$*),

131.09 (*Ar*-H), 130.88 (*Ar*$_{quart}$), 129.00, 128.26, 119.30, 115.30, 114.42, 111.16 (*Ar*-H), 71.28 (-O*C*H-), 70.84, 70.70, 68.09, 64.20, 64.13 (-O*C*H$_2$-), 37.92, 33.36, 30.98 (-*C*H$_2$-), 28.73 (-*C*H$_3$), 20.71 (-*C*H$_2$-), 13.22, 7.52 (Si-*C*H$_2$-), -5.55 (Si-*C*H$_3$). **^{29}Si-NMR** (80 MHz, Pyridine-d$_5$): δ / ppm = 4.63 (s, *Si*). **HR-MS** (m/z): [M]+Li$^+$ calcd. For $C_{41}H_{62}O_7SiLi$, 701.4420, found: 701.4462.

2'-{4-[Bis(2-cyclohexylethyl)methylsilyl]-*n*-but-1-yloxy}-4,4´´-bis(2,3-dihydroxypropyloxy)-*p*-terphenyl 2c8: Synthesized from **2c8A**(560 mg, 0.7 mmol), PPTS (340 mg, 1.4 mmol) in MeOH (25 mL) and THF (25 mL), yield: 350 mg, 0.5 mmol, (69 %), colorless solid; **^{1}H-NMR** (400 MHz, Pyridine-d$_5$): δ / ppm = 7.85 – 7.69 (m, 4H, Aryl-*H*), 7.53 (d, $^3J_{H,H}$ = 7.8 Hz, 1H, Aryl-*H*), 7.45 (d, $^4J_{H,H}$ = 1.7 Hz, 1H, Aryl-*H*), 7.39 (dd, $^3J_{H,H}$ = 7.9 Hz, $^4J_{H,H}$ = 1.7 Hz, 1H, Aryl-*H*), 7.28 – 7.21 (m, 4H, Aryl-*H*), 6.25 (broad, 4H, -O*H*), 4.66 – 4.53 (m, 4H, -OC*H$_2$*-), 4.52 – 4.44 (m, 2H, -OC*H*-), 4.30 – 4.18 (m, 4H, -OC*H$_2$*-), 4.14 (t, $^3J_{H,H}$ = 6.3 Hz, 2H, -OC*H$_2$*-), 1.93 – 1.81 (m, 2H, -C*H$_2$*-), 1.81 – 1.48 (m, 13H, -C*H*-, -C*H$_2$*-), 1.34 – 1.03 (m, 11H, -C*H$_2$*-, -C*H*-), 0.93 – 0.79 (m, 4H, -C*H$_2$*-), 0.65 – 0.51 (m, 6H, Si-C*H$_2$*-), 0.06 (s, 3H, Si-C*H$_3$*).**^{13}C-NMR** (101 MHz, Pyridine-d$_5$): δ / ppm = 159.39, 158.71, 156.75 (*Ar*-O), 141.08, 133.55, 131.16 (*Ar*$_{quart}$), 131.11 (*Ar*-H), 130.90 (*Ar*$_{quart}$), 129.02, 128.27, 119.32, 115.31, 114.43, 111.18 (*Ar*-H), 71.29 (-O*C*H-), 70.82, 70.69, 68.06, 64.20, 64.13 (-O*C*H$_2$-), 40.84 (-*C*H-), 33.34, 33.03, 31.52, 26.83, 26.50, 20.74 (-*C*H$_2$-), 13.40, 10.56 (Si-*C*H$_2$-), -5.32 (Si-*C*H$_3$). **^{29}Si-NMR** (80 MHz, Pyridine-d$_5$): δ / ppm = 3.72 (s, *Si*). **HR-MS** (m/z): [M]+Li$^+$ calcd. For $C_{45}H_{66}O_7SiLi$, 753.4733, found: 753.4722.

2'-[4-(Tricyclobutylsilyl)-*n*-but-1-yloxy]-4,4´´-bis(2,3-dihydroxypropyloxy)-*p*-terphenyl 3c4: Synthesized from **3c4A** (530 mg, 0.7 mmol), PPTS (352 mg, 1.4 mmol) in MeOH (25 mL) and THF (25 mL), yield: 200 mg, 0.3 mmol, (43 %), colorless solid; **^{1}H-NMR** (400 MHz, Pyridine-d$_5$): δ / ppm = 7.84 – 7.70 (m, 4H*, Aryl-H*), 7.53 (d, $^3J_{H,H}$ = 7.9 Hz, 1H*, Aryl-H*), 7.44 (d, $^4J_{H,H}$ = 1.7 Hz, 1H*, Aryl-H*), 7.38 (dd, $^3J_{H,H}$ = 7.8 Hz, $^4J_{H,H}$ = 1.7 Hz, 1H*, Aryl-H*), 7.29 – 7.21 (m, 4H*, Aryl-H*), 6.11 (s, 4H*, -OH*), 4.68 – 4.53 (m, 4H*, -OCH$_2$*-), 4.53 – 4.44 (m, 2H*, -OCH*-), 4.31 – 4.19 (m, 4H*, -OCH$_2$*-), 4.12 (t, $^3J_{H,H}$ = 6.2 Hz, 2H*, -OCH$_2$*-), 2.30 – 2.12 (m, 3H, Si-C*H*-), 2.12 – 1.88 (m, 18H*, -CH$_2$*-), 1.88 – 1.73 (m, 2H*, -CH$_2$*-), 1.65 – 1.46 (m, 2H*, -CH$_2$*-), 0.74 – 0.59 (m, 2H*, Si-CH$_2$*-). **^{13}C-NMR** (101 MHz, Pyridine-d$_5$): δ / ppm = 159.39, 158.73, 156.74 (*Ar*-O), 141.07, 133.54, 131.15 (*Ar*$_{quart}$), 131.10 (*Ar*-H), 130.89 (*Ar*$_{quart}$), 129.01, 128.27, 119.29, 115.31, 114.40, 111.15 (*Ar*-H), 71.31 *(*-O*C*H-*)*, 70.84, 70.74, 68.09, 64.22, 64.14 *(*-O*C*H$_2$-*)*, 33.80, 24.50, 24.09, 21.07 *(*-*C*H$_2$-*)*, 19.56 *(*Si-*C*H-), 8.99 *(*Si-*C*H$_2$-*)*. **^{29}Si-NMR** (80 MHz, Pyridine-d$_5$): δ / ppm = -3.66 (s, *Si*). **HR-MS** (m/z): [M]+Li$^+$ calcd. For $C_{40}H_{54}O_7SiLi$, 681.3794, found: 681.3793.

2'-{4-[Tris(3-methylbutyl)silyl]-*n*-but-1-yloxy}-4,4´´-bis(2,3-dihydroxypropyloxy)-*p*-terphenyl 3i5: Synthesized from **3i5A** (620 mg, 0.8 mmol), PPTS (388 mg, 1.6 mmol) in MeOH (25 mL) and THF (25 mL), yield: 389 mg, 0.5 mmol, (70 %), colorless solid; **^{1}H-NMR** (400 MHz, Pyridine-d$_5$): δ / ppm = 7.75 (dd, $^3J_{H,H}$ = 8.7 Hz, $^4J_{H,H}$ = 4.4 Hz, 4H, Aryl-*H*), 7.51 (d, $^3J_{H,H}$ = 7.9 Hz, 1H, Aryl-*H*), 7.41 (d, $^4J_{H,H}$ = 1.7 Hz, 1H, Aryl-*H*), 7.36 (dd, $^3J_{H,H}$ = 8.0 Hz, $^4J_{H,H}$ = 1.5 Hz, 1H, Aryl-*H*), 7.28 – 7.17 (m, 4H, Aryl-*H*), 6.48 (s, 4H, -O*H*), 4.67 – 4.49 (m, 4H, -OC*H$_2$*-), 4.48 – 4.38 (m, 2H, -OC*H*-), 4.31 – 4.16 (m, 4H, -OC*H$_2$*-), 4.11 (t, $^3J_{H,H}$ = 6.3 Hz, 2H, -OC*H$_2$*-), 1.93 – 1.75 (m, 2H, -C*H$_2$*-), 1.63 – 1.54 (m, 2H, -C*H$_2$*-), 1.53 – 1.41 (m, 3H, -C*H*-), 1.38 – 1.19 (m, 6H, -C*H$_2$*-), 0.97 – 0.82 (m, 18H, -C*H$_3$*), 0.67 – 0.50 (m, 8H, Si-C*H$_2$*-). **^{13}C-NMR** (101 MHz, Pyridine-d5) δ / ppm = 159.37, 158.69, 156.71 (*Ar*-O), 141.05, 133.52, 131.13 (*Ar*$_{quart}$), 131.09 (*Ar*-H), 130.86 (*Ar*$_{quart}$), 128.95, 128.24, 119.29, 115.28, 114.40, 111.14 (*Ar*-H), 71.27 (-O*C*H-), 70.82, 70.68, 68.05, 64.19, 64.11 (-O*C*H$_2$-), 33.48, 33.03 (-*C*H$_2$-), 31.15 (-*C*H-), 22.09 (-*C*H$_3$), 20.74 (-*C*H$_2$-), 12.10, 9.55 (Si-*C*H$_2$-). **^{29}Si-NMR** (100 MHz, Pyridine-d$_5$): δ / ppm = 4.17 (s, *Si*). **HR-MS** (m/z): [M]+Li$^+$ calcd. For $C_{43}H_{66}O_7SiLi$, 729.4733, found: 729.4743.

2'-{4-[Tris(3,3-dimethylbutyl)silyl]-*n*-but-1-yloxy}-4,4´´-bis(2,3-dihydroxypropyloxy)-*p*-terphenyl 3t6: Synthesized from **3t6A** (590 mg, 0.7 mmol), PPTS (345 mg, 1.4 mmol) in MeOH (25 mL) and THF (25 mL), yield: 252 mg, 0.3 mmol, (47 %), colorless solid; **^{1}H-NMR** (400 MHz, Pyridine-d$_5$): δ / ppm = 7.85 – 7.70 (m, 4H, Aryl-*H*), 7.53 (d, $^3J_{H,H}$ = 7.9 Hz, 1H, Aryl-*H*), 7.44 (d, $^4J_{H,H}$ = 1.7 Hz, 1H, Aryl-*H*), 7.38 (dd, $^3J_{H,H}$ = 7.8 Hz, $^4J_{H,H}$ = 1.7 Hz, 1H, Aryl-*H*), 7.29 – 7.20 (m, 4H, Aryl-*H*), 6.87 (s, 2H, -O*H*), 6.48 (s, 2H, -O*H*), 4.64 – 4.52 (m, 4H, -OC*H*-), 4.52 – 4.43 (m, 2H, -OC*H*-), 4.31 – 4.19 (m, 4H, -OC*H$_2$*-), 4.15 (t, $^3J_{H,H}$ = 6.4 Hz, 2H, -OC*H$_2$*-), 2.00 – 1.80 (m, 2H, -C*H$_2$*-), 1.74 – 1.56 (m, 2H, -C*H$_2$*-), 1.38 – 1.27 (m, 6H, -C*H$_2$*-), 0.92 (s, 27H, -C*H$_3$*), 0.74 – 0.67 (m, 2H, Si-C*H$_2$*-), 0.66 – 0.41 (m, 6H, Si-C*H$_2$*-). **^{13}C-NMR** (126 MHz,

Pyridine-d5) δ / ppm = 159.39, 158.71, 156.73 (*Ar*-O), 141.07, 133.55, 131.15 (Ar_{quart}), 131.12 (*Ar*-H), 130.87 (Ar_{quart}), 128.96, 128.27, 119.33, 115.30, 114.43, 111.19 (*Ar*-H), 71.29 (-O*C*H-), 70.85, 70.70, 68.13, 64.22, 64.14 (-OCH_2-), 37.95, 33.51, 31.07 (-CH_2-), 28.75 (-CH_3), 20.74 (-CH_2-), 11.86, 5.97 (Si-CH_2-). **^{29}Si-NMR** (100 MHz, Pyridine-d_5): δ / ppm = 5.66 (s, *Si*). **HR-MS** (m/z): $[M]+Li^+$ calcd. For $C_{46}H_{72}O_7SiLi$, 771.5203, found: 771.5220.

3.4 NMR Spectra

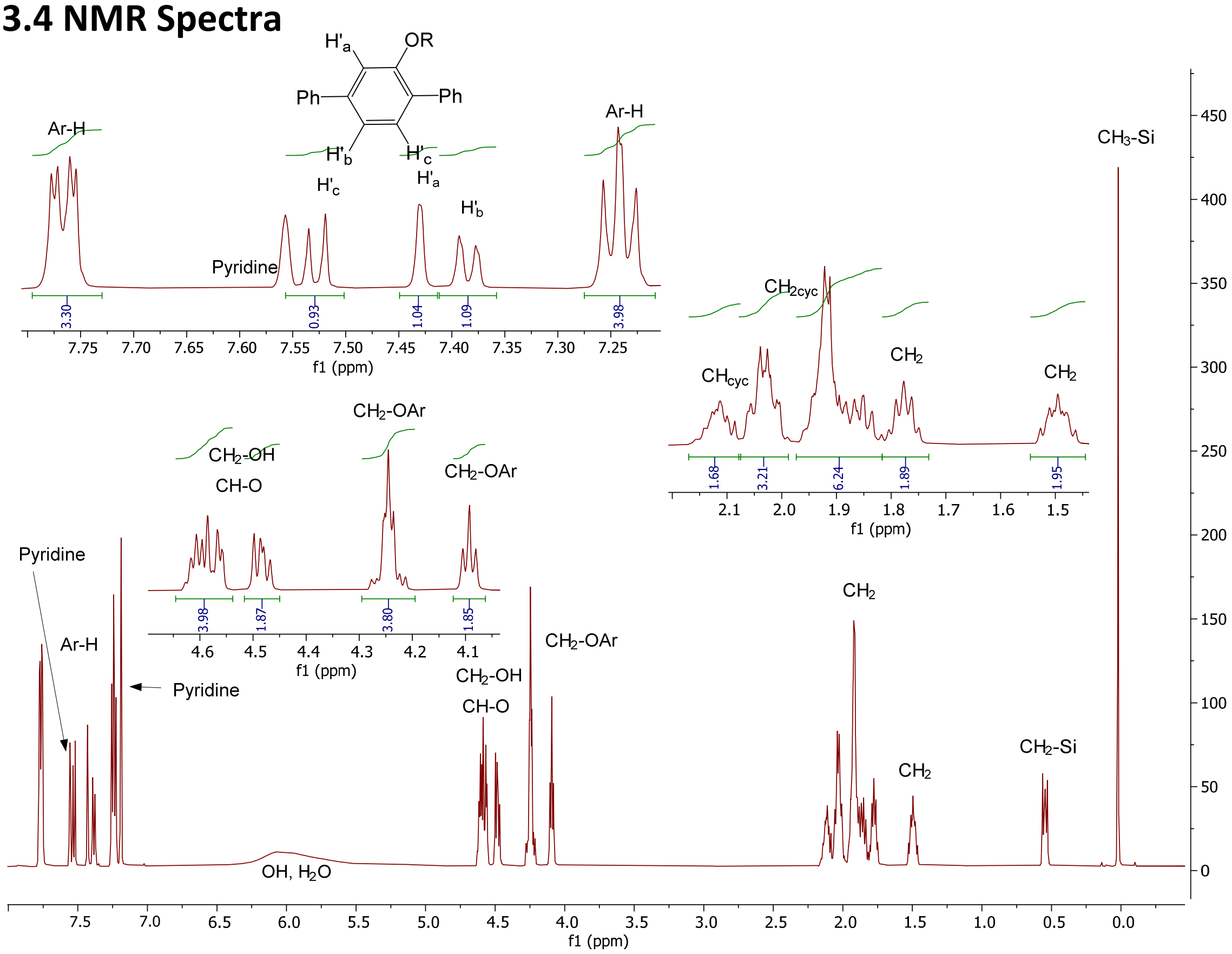

Figure S7a: ^{1}H-NMR-Spectrum of **2*c*4** (500 MHz, pyridine-d$_5$).

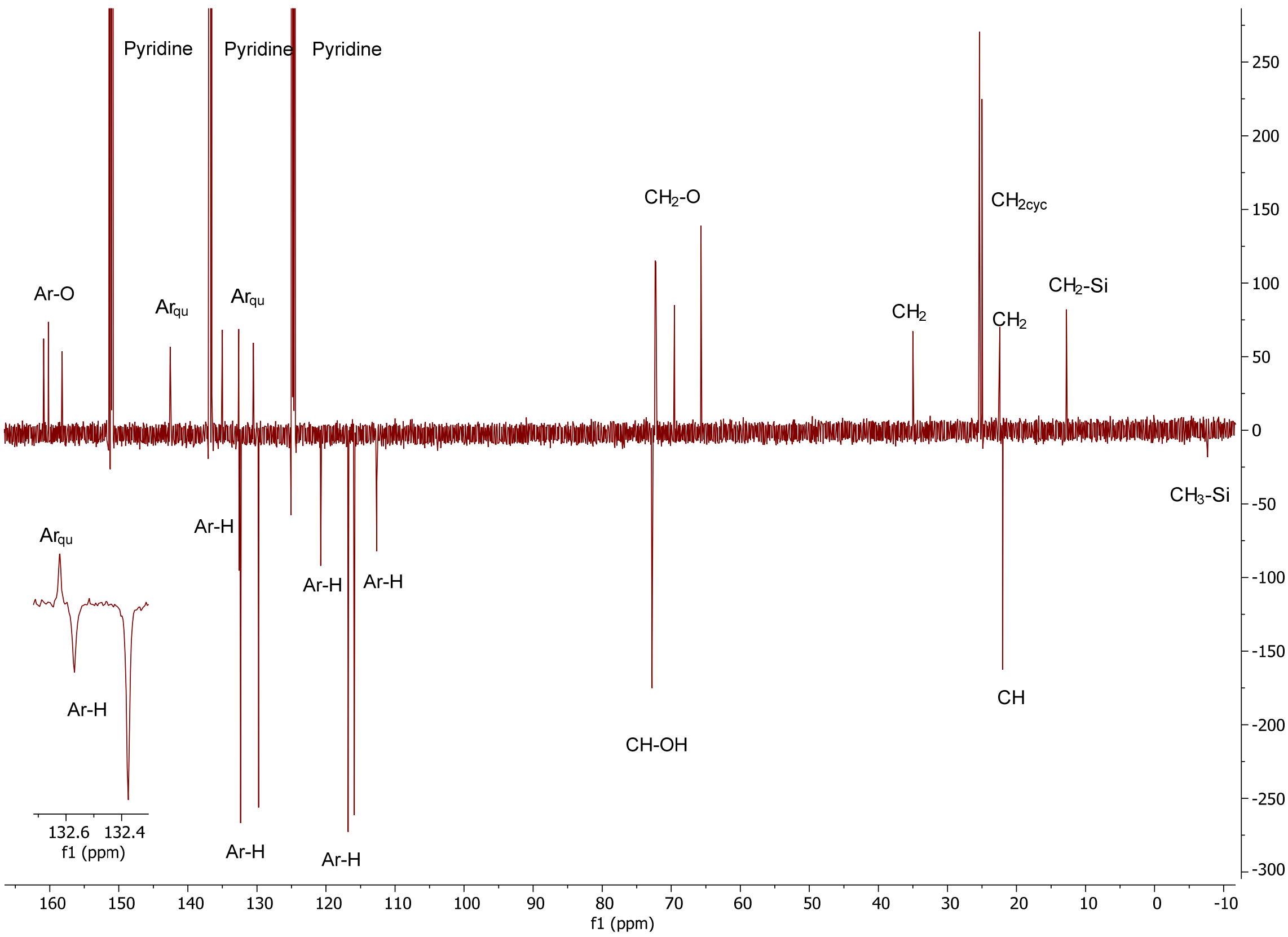

Figure S7b: ^{13}C-NMR-Spectrum of **2c4** (126 MHz, pyridine-d$_5$).

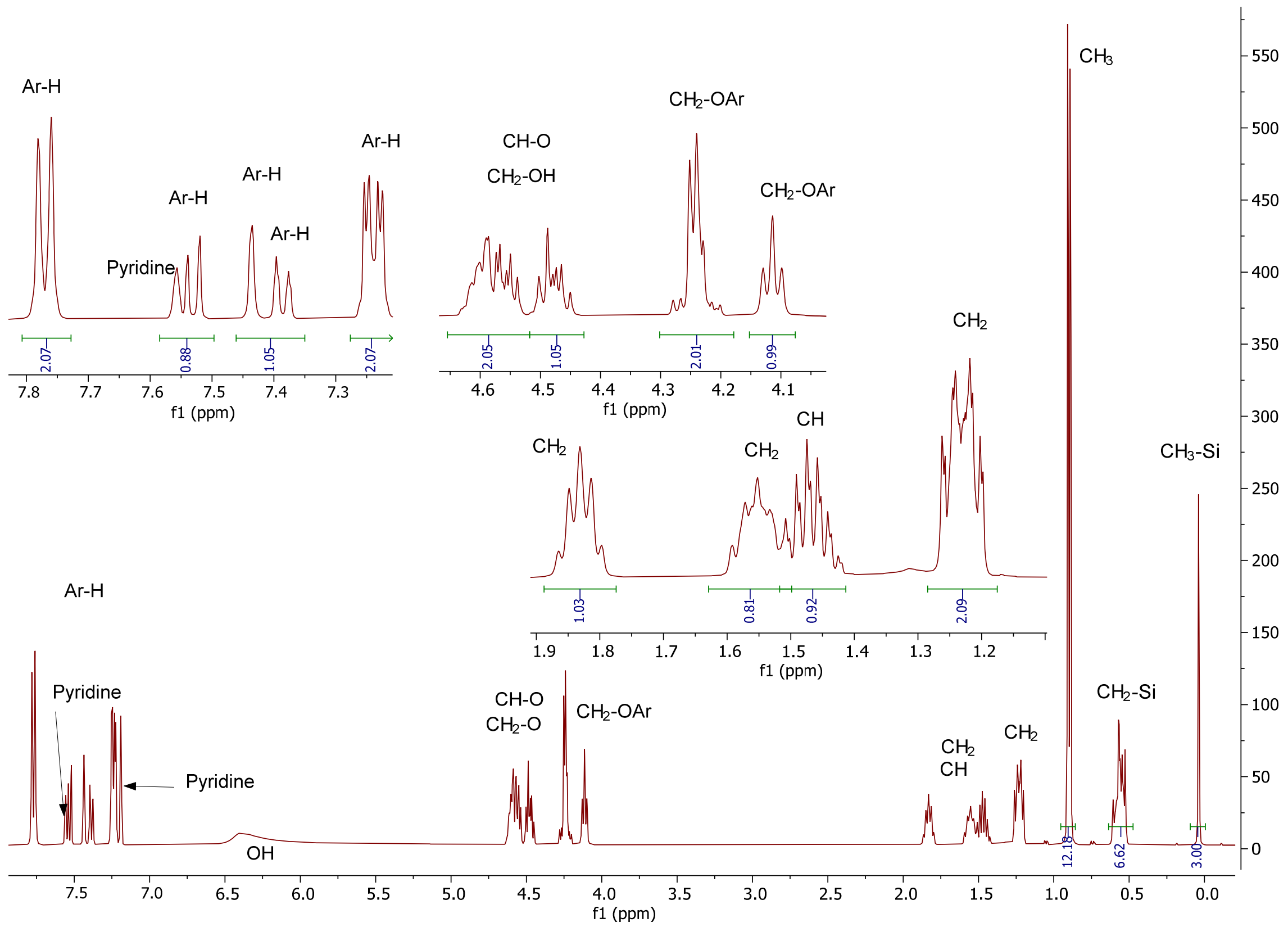

Figure S8a: ^{1}H-NMR-Spectrum of **2*i*5** (400 MHz, pyridine-d$_5$).

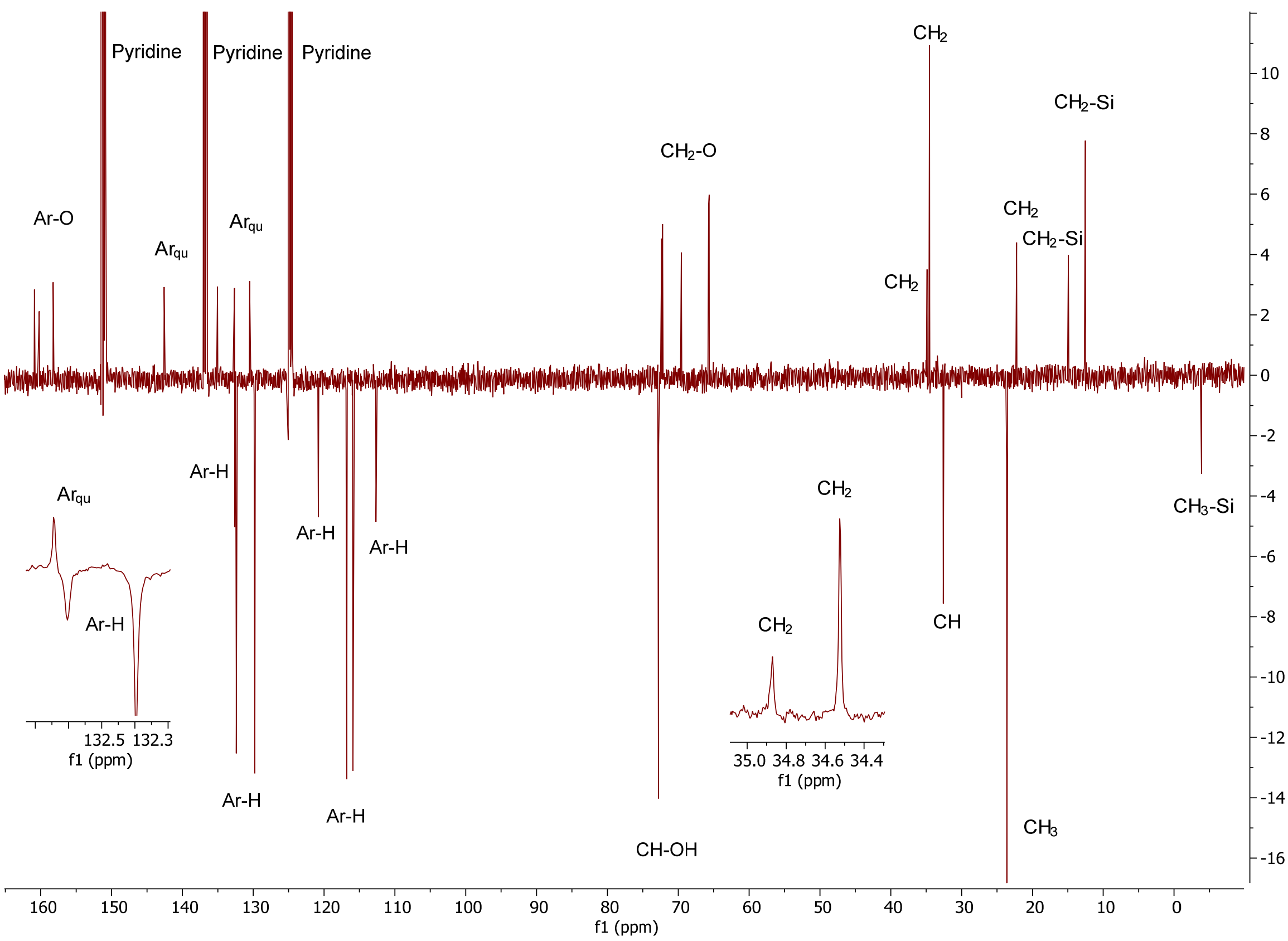

Figure S8b: ^{13}C-NMR-Spectrum of **2*i*5** (101 MHz, pyridine-d$_5$).

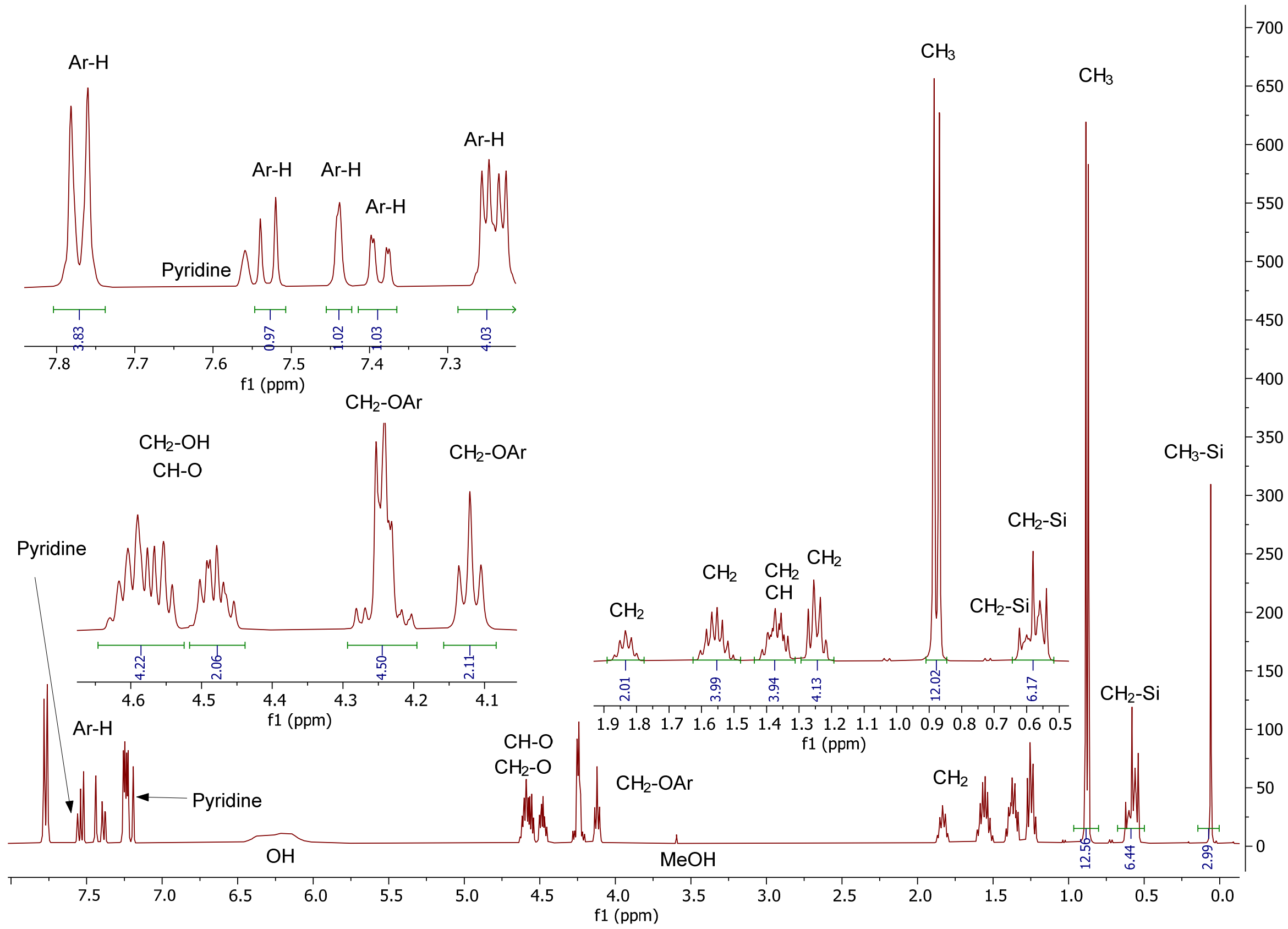

Figure S9a: ^{1}H-NMR-Spectrum of **2*i*6** (400 MHz, pyridine-d$_5$).

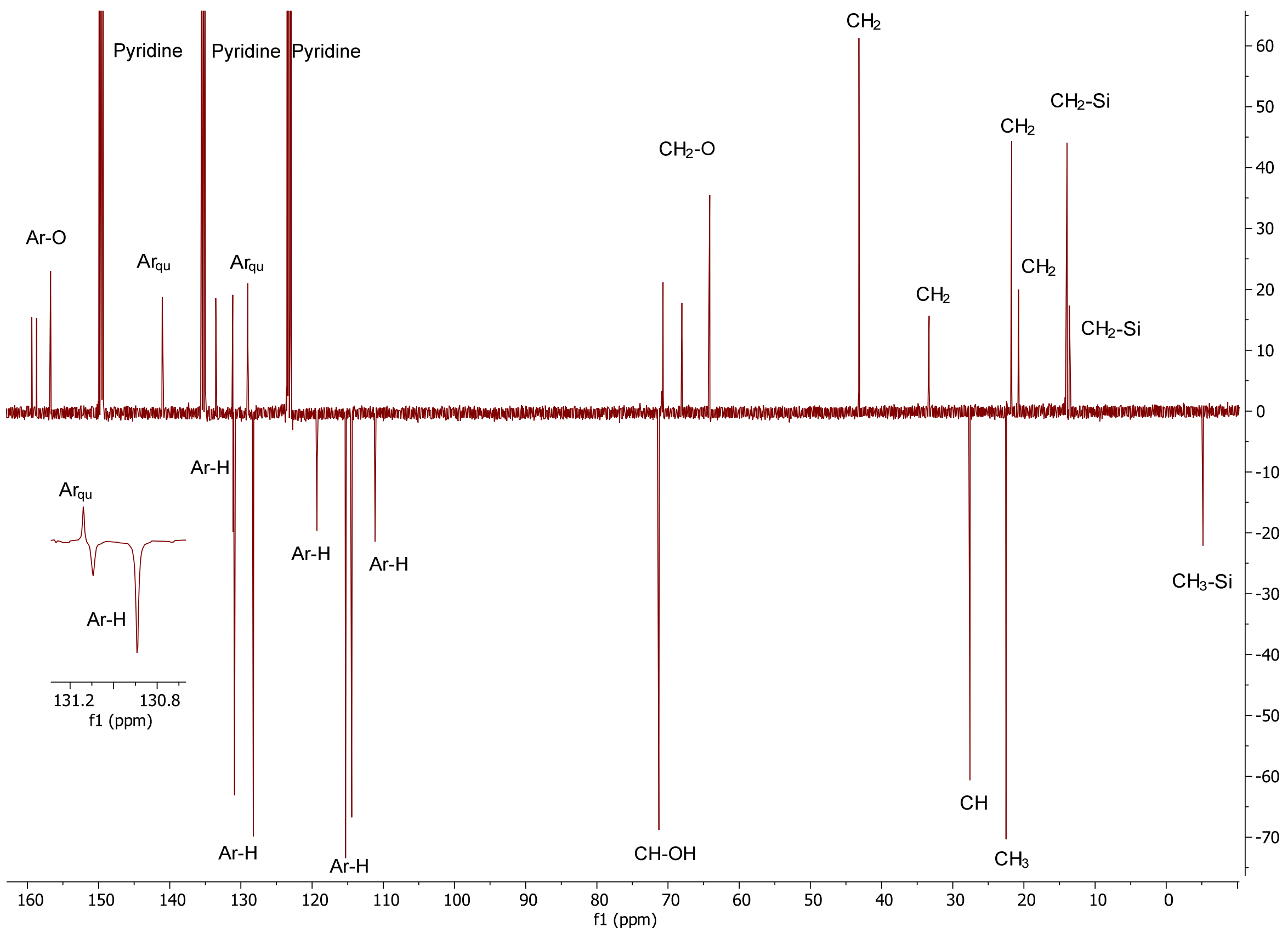

Figure S9b: ^{13}C-NMR-Spectrum of **2*i*6** (101 MHz, pyridine-d$_5$).

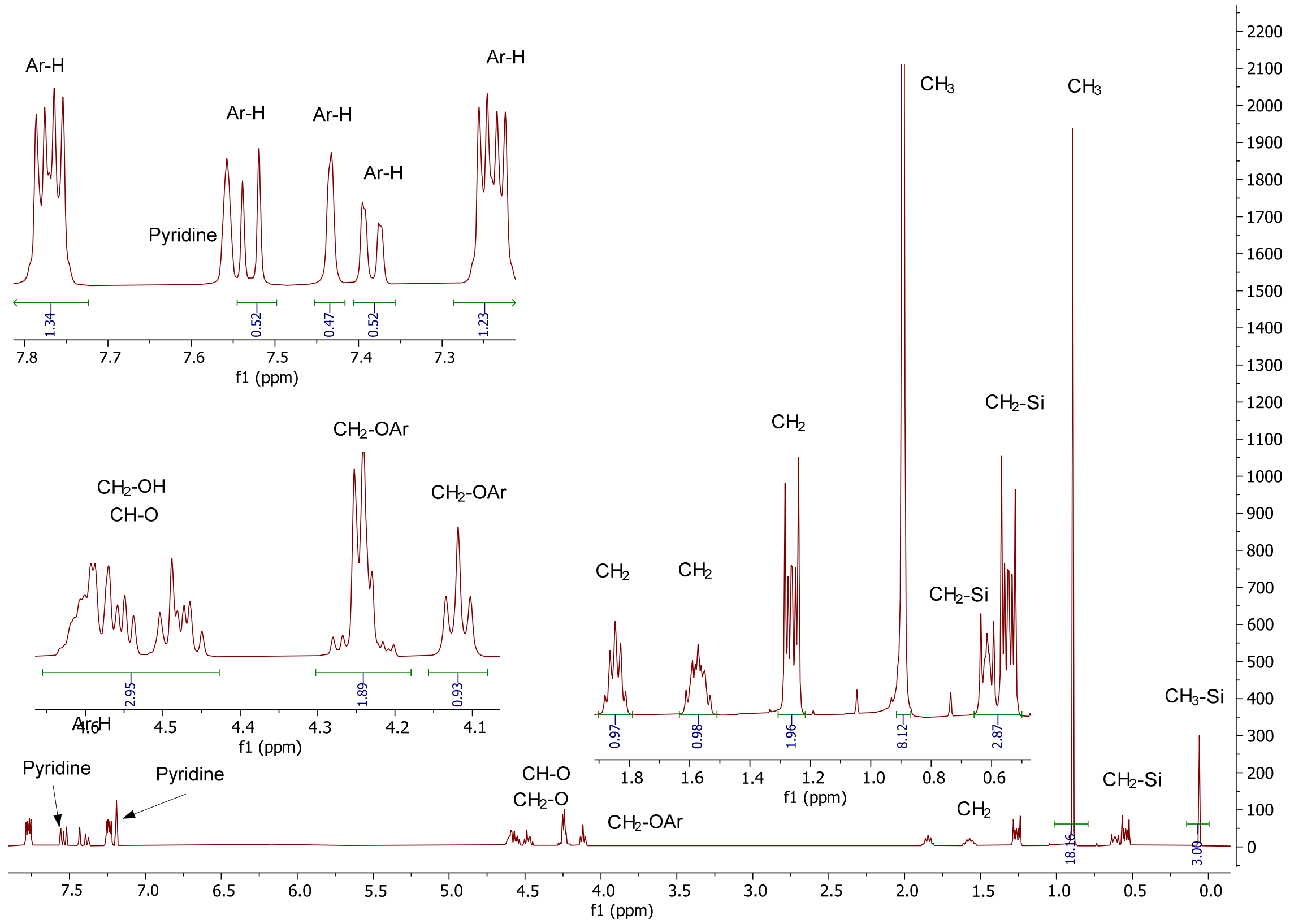

Figure S10a: ^{1}H-NMR-Spectrum of **2*t*6** (400 MHz, pyridine-d$_5$).

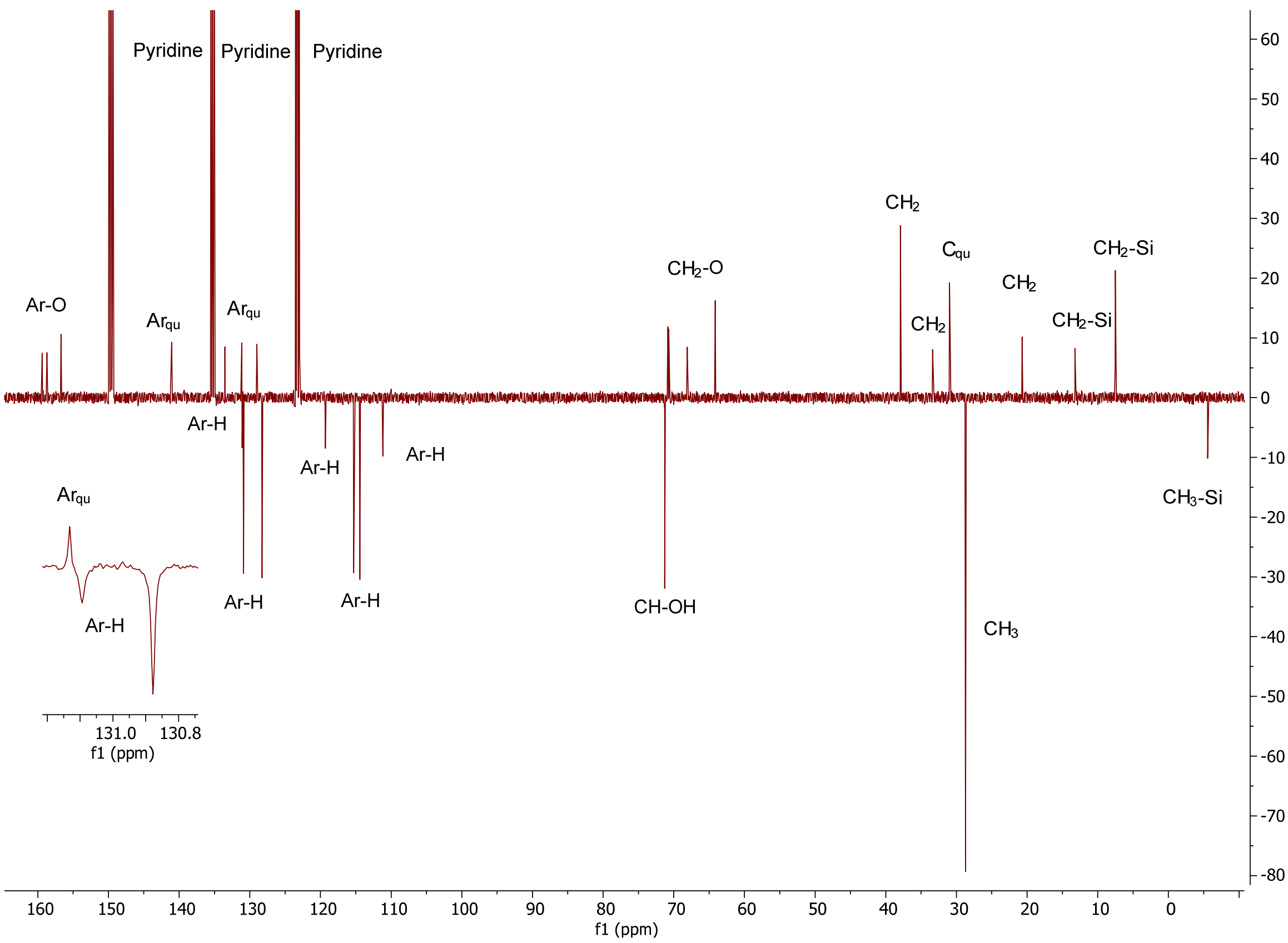

Figure S10b: ^{13}C-NMR-Spectrum of **2*t*6** (101 MHz, pyridine-d$_5$).

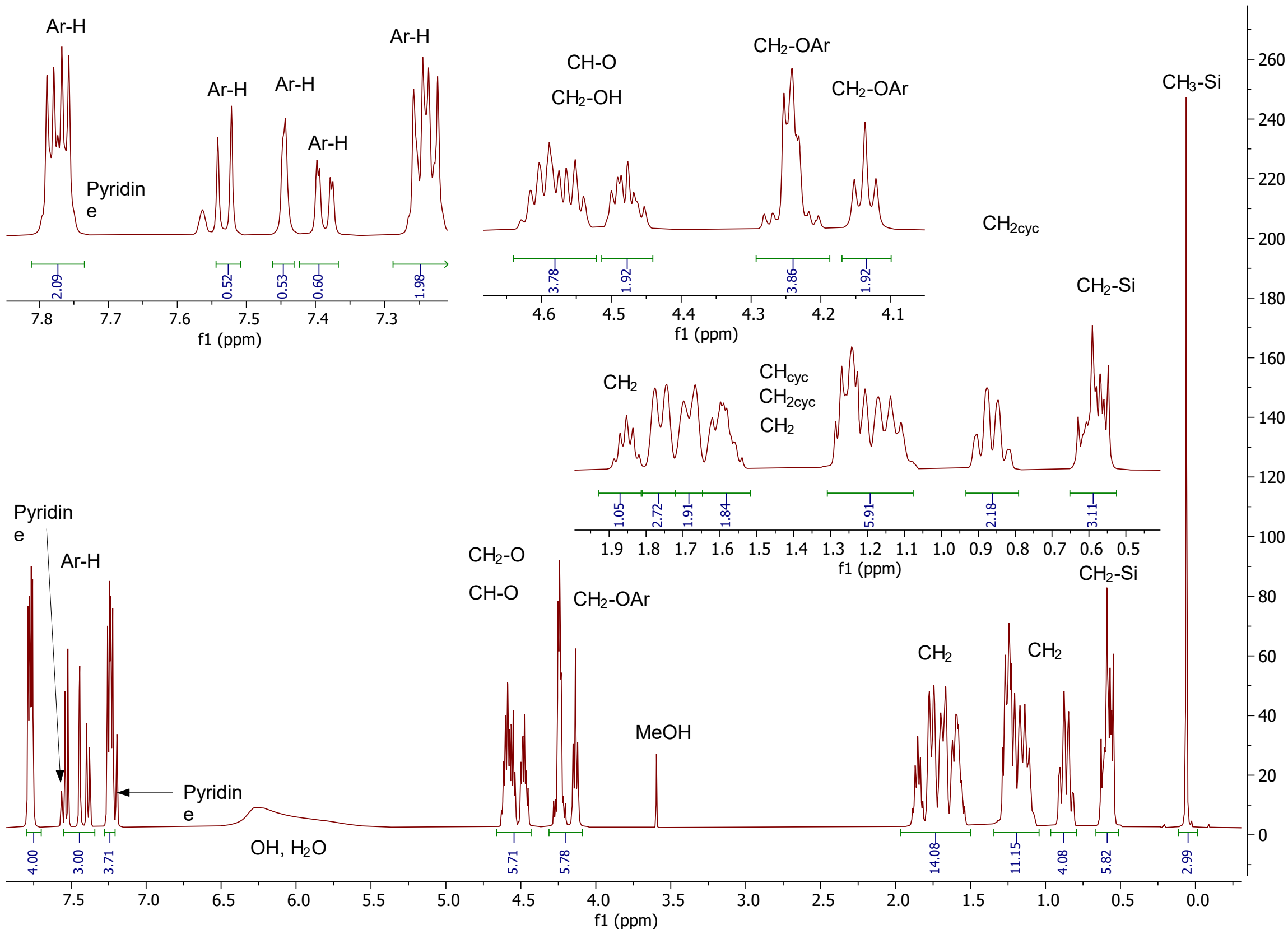

Figure S11a: ^{1}H-NMR-Spectrum of **2*c*8** (400 MHz, pyridine-d$_5$).

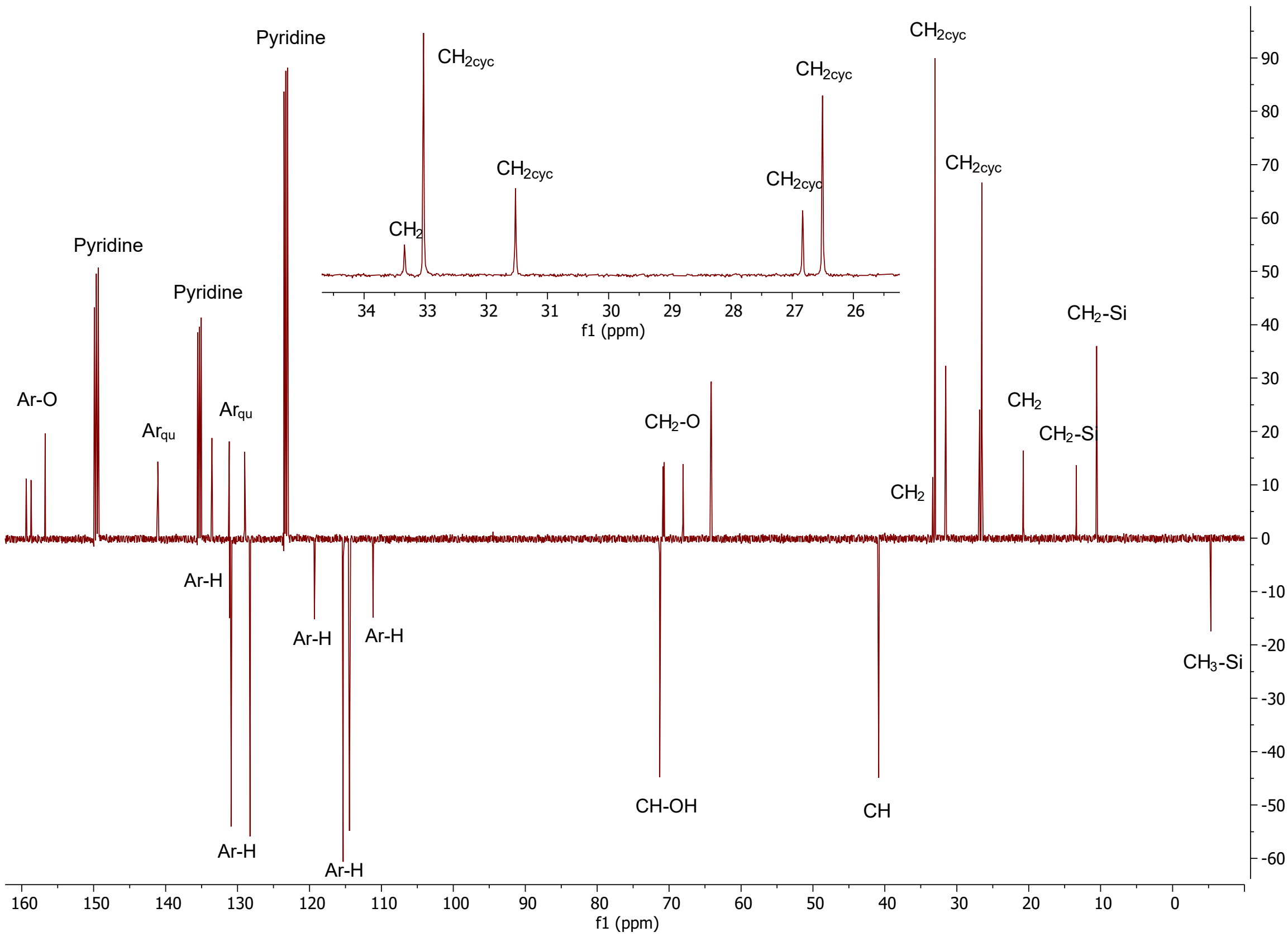

Figure S11b: ^{13}C-NMR-Spectrum of **2*c*8** (101 MHz, pyridine-d$_5$).

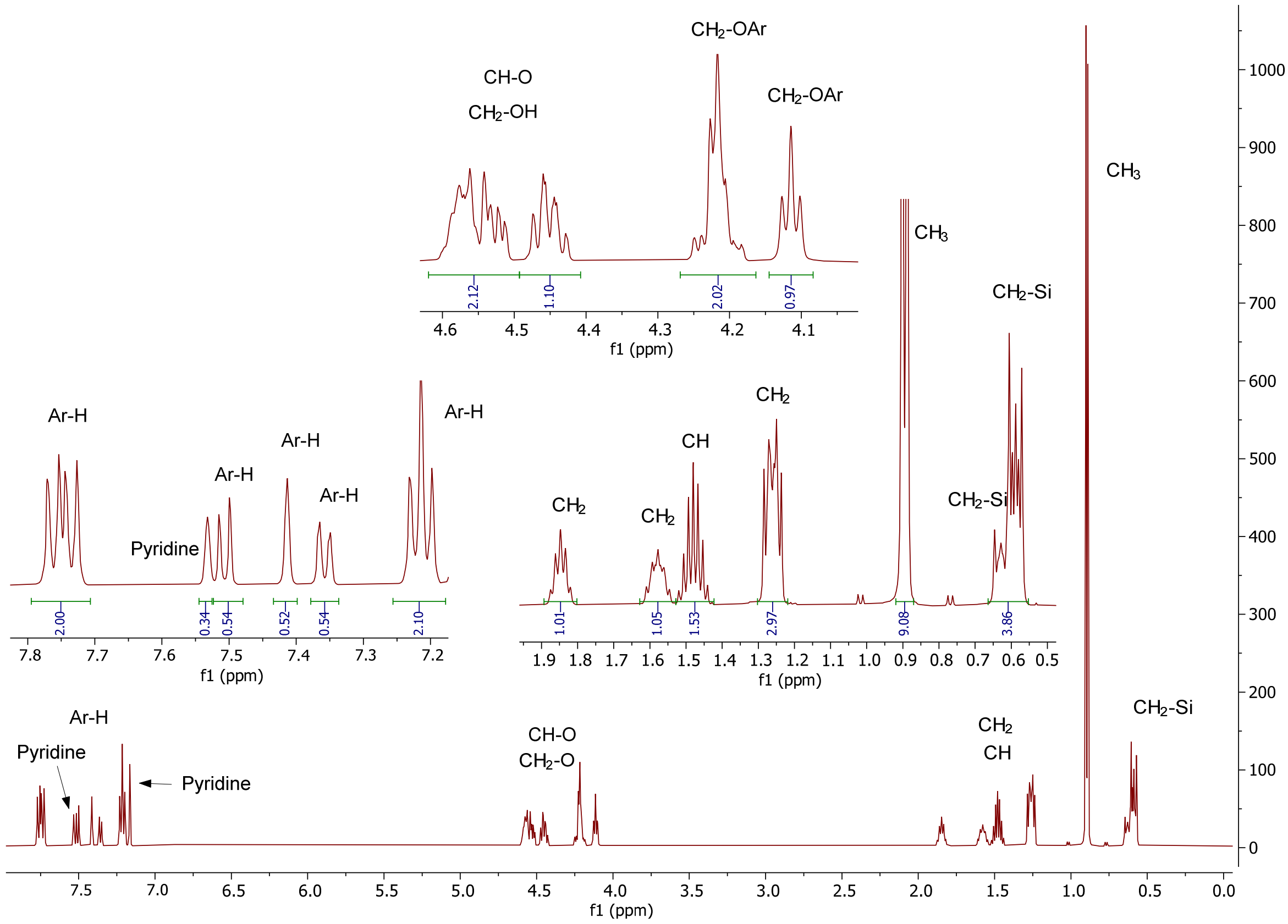

Figure S12a: ^{1}H-NMR-Spectrum of **3i5** (400 MHz, pyridine-d_5).

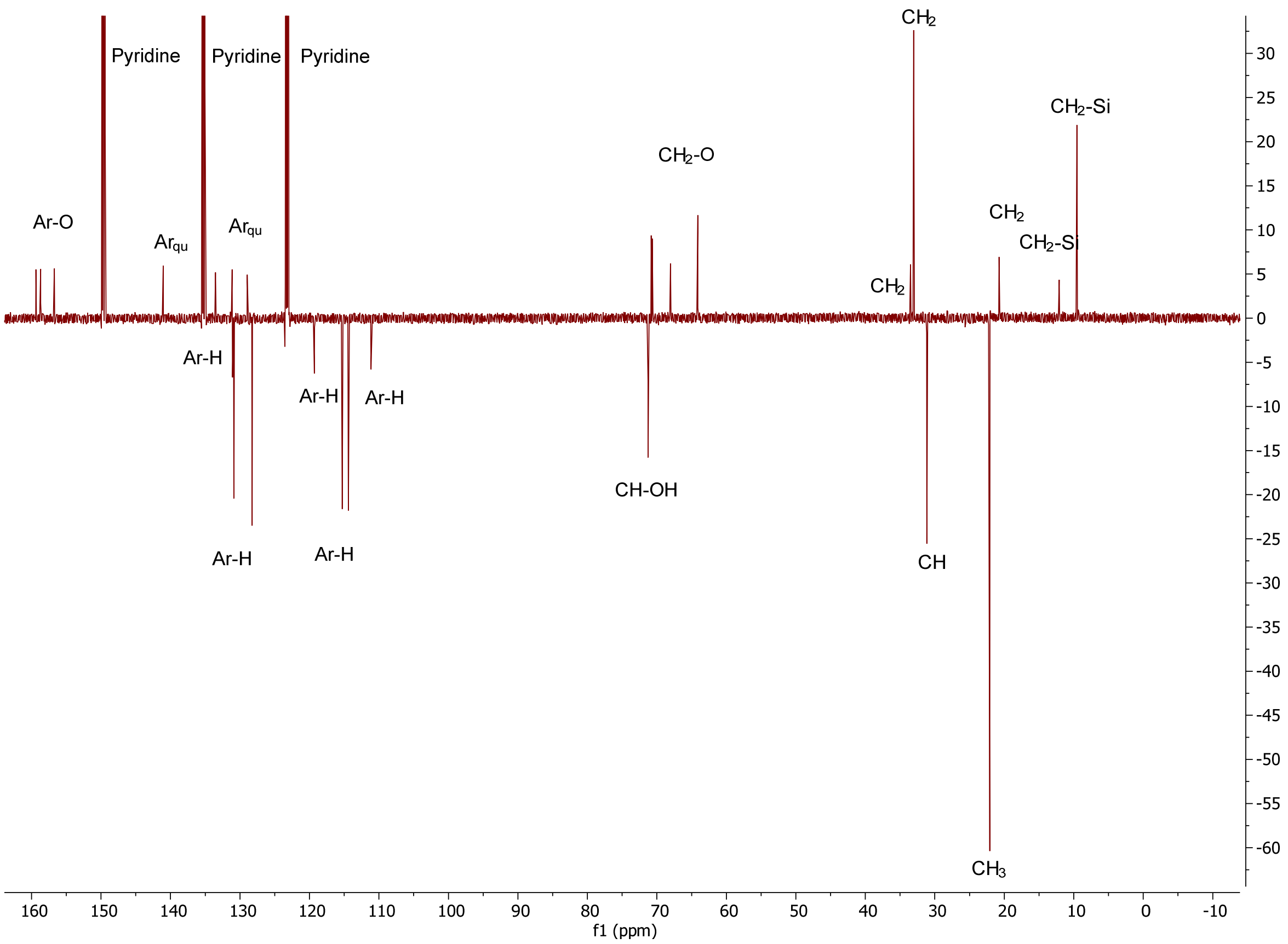

Figure S12b: ^{13}C-NMR-Spectrum of **3i5** (101 MHz, pyridine-d_5).

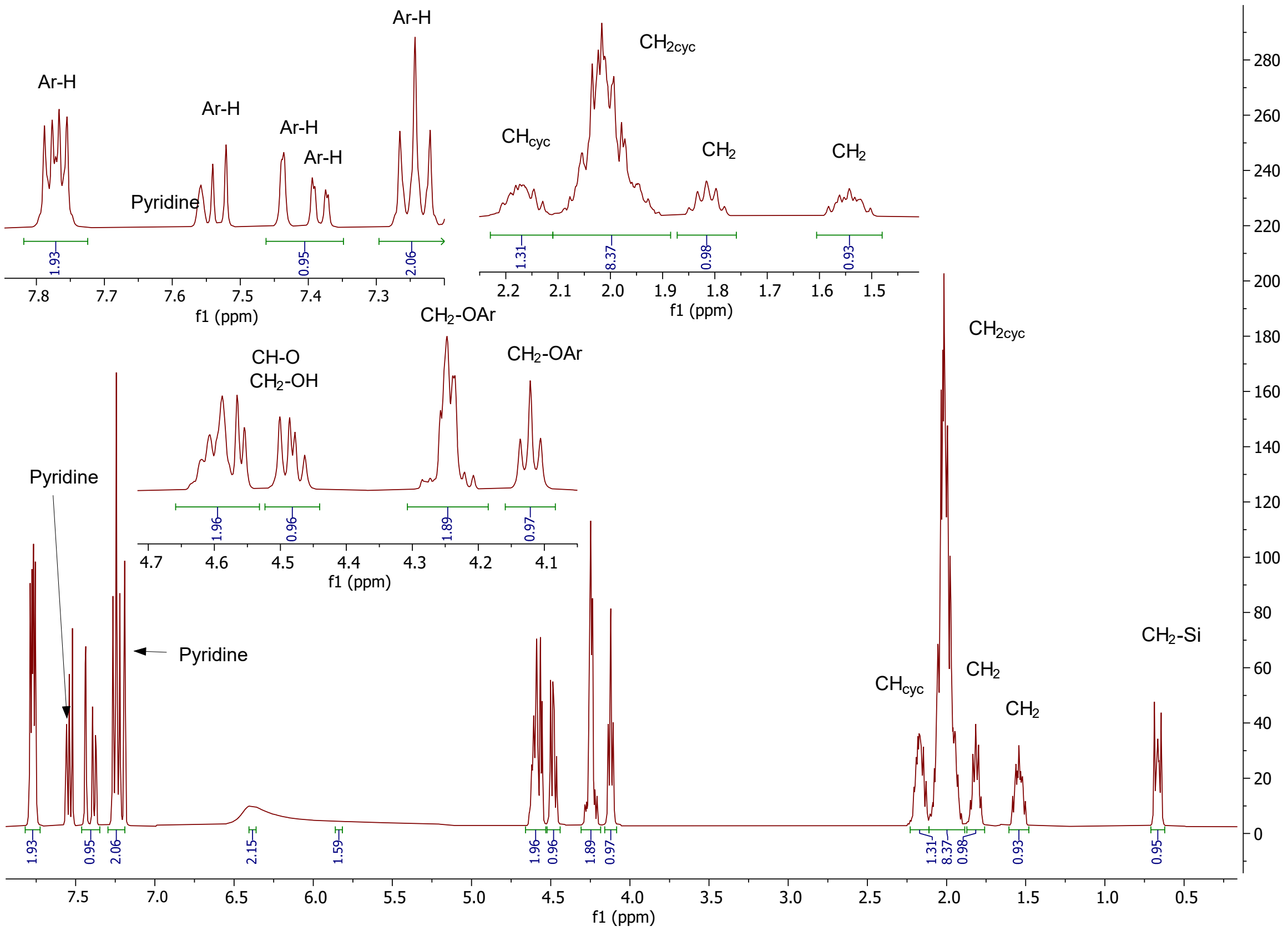

Figure S13a: ^{1}H-NMR-Spectrum of **3c4** (400 MHz, pyridine-d_5).

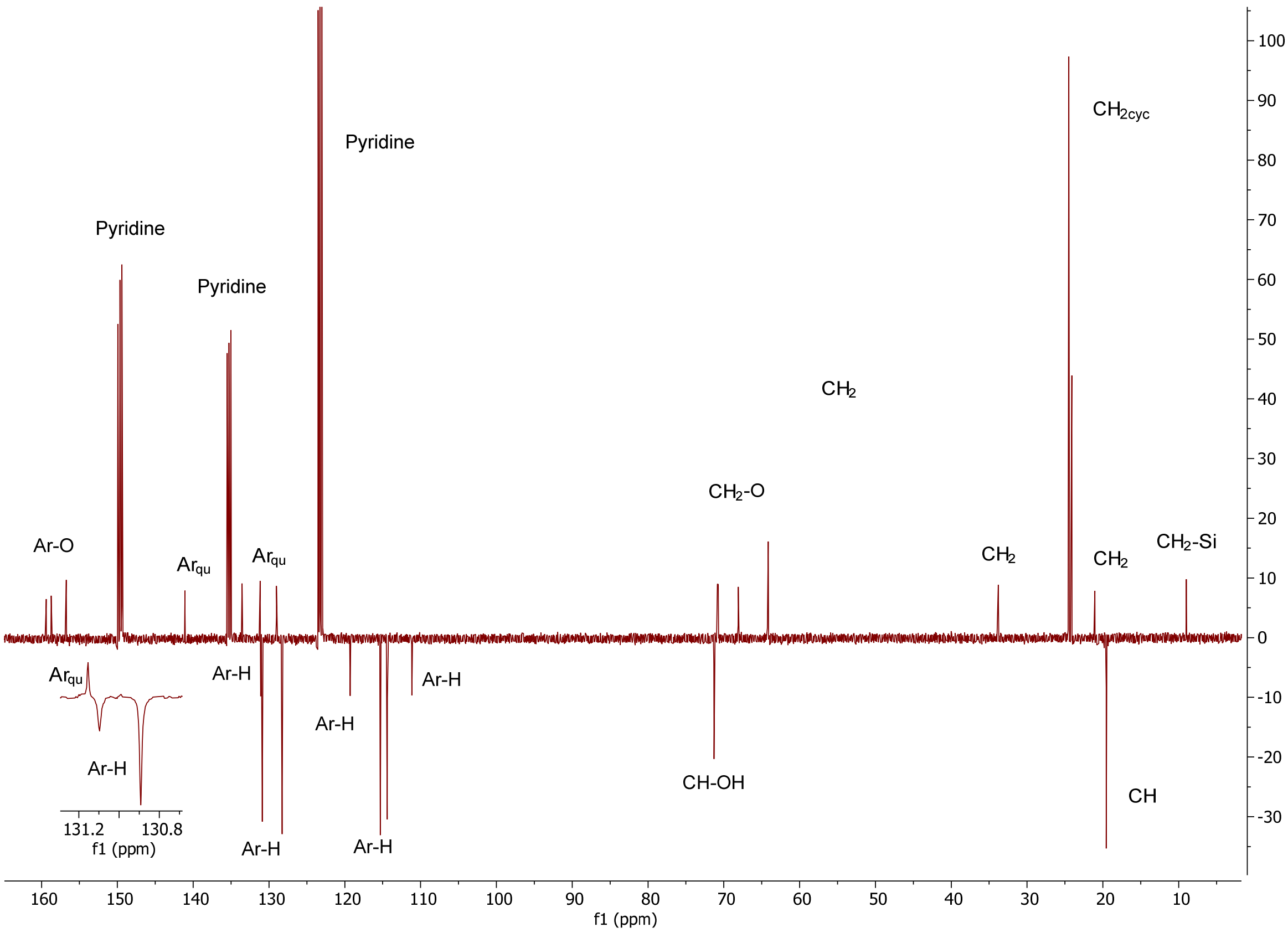

Figure S13b: ^{13}C-NMR-Spectrum of **3c4** (101 MHz, pyridine-d_5).

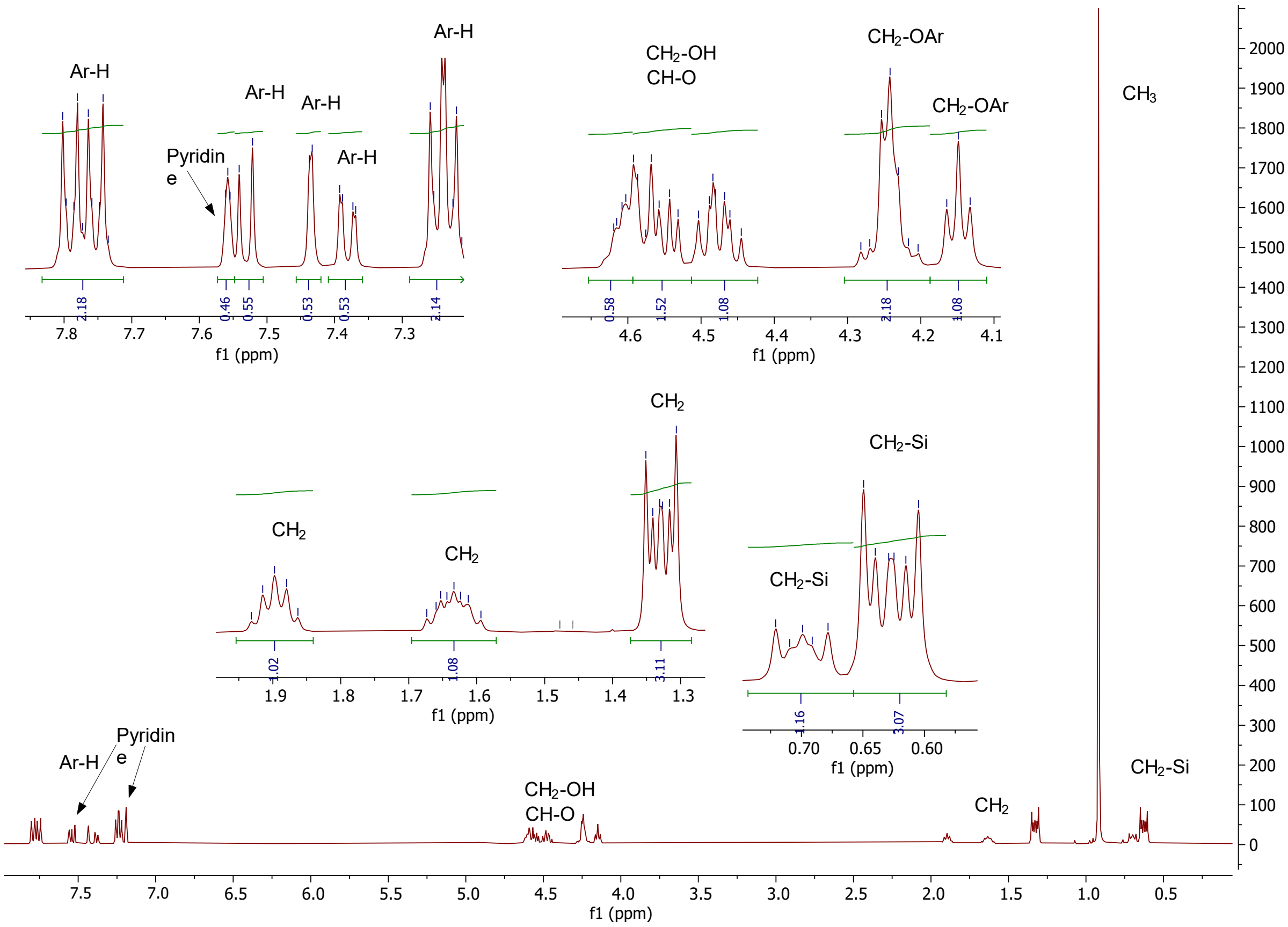

Figure S14a: ^{1}H-NMR-Spectrum of **3*t*6** (400 MHz, pyridine-d$_5$).

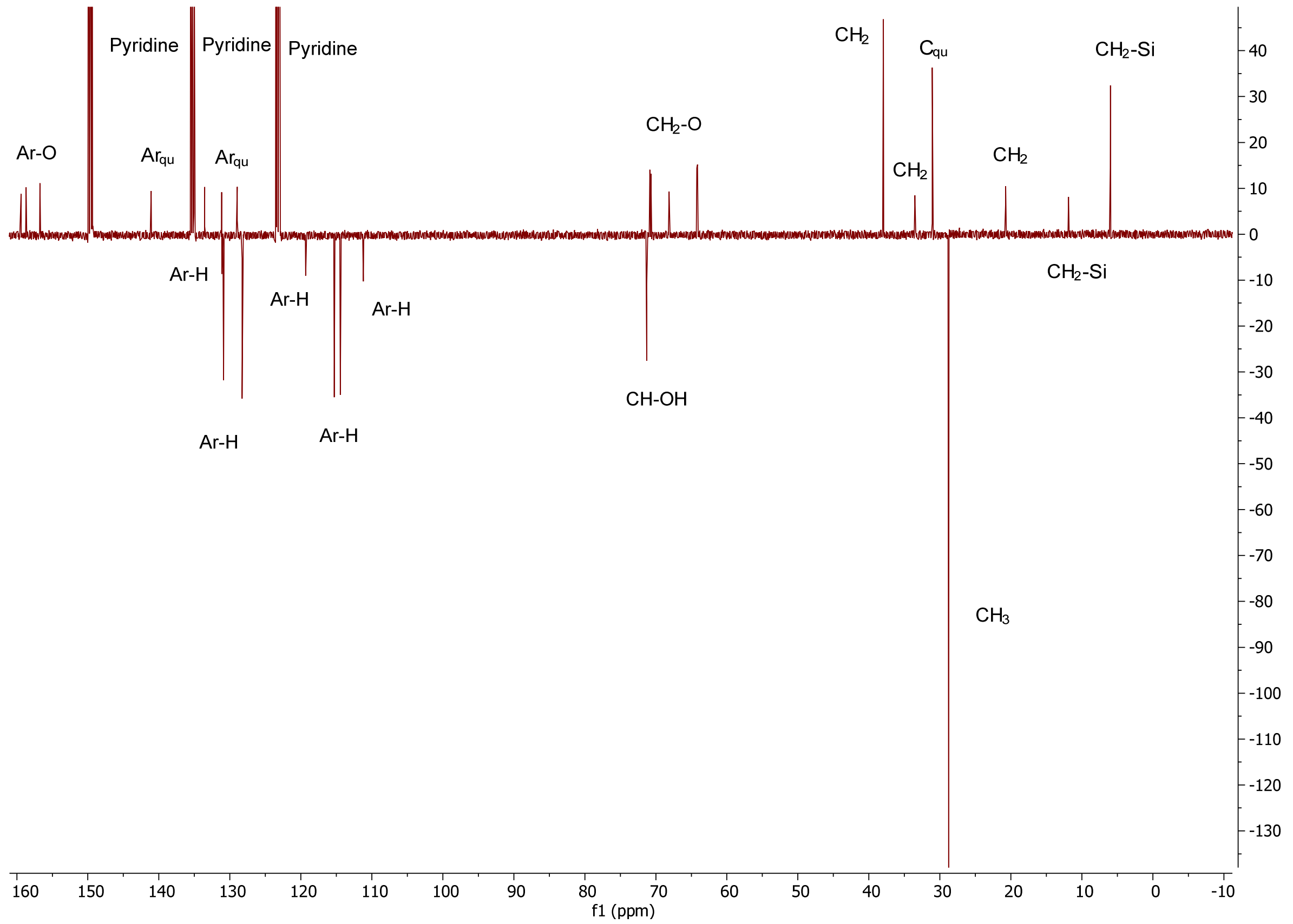

Figure S14b: ^{13}C-NMR-Spectrum of **3*t*6** (101 MHz, pyridine-d$_5$).

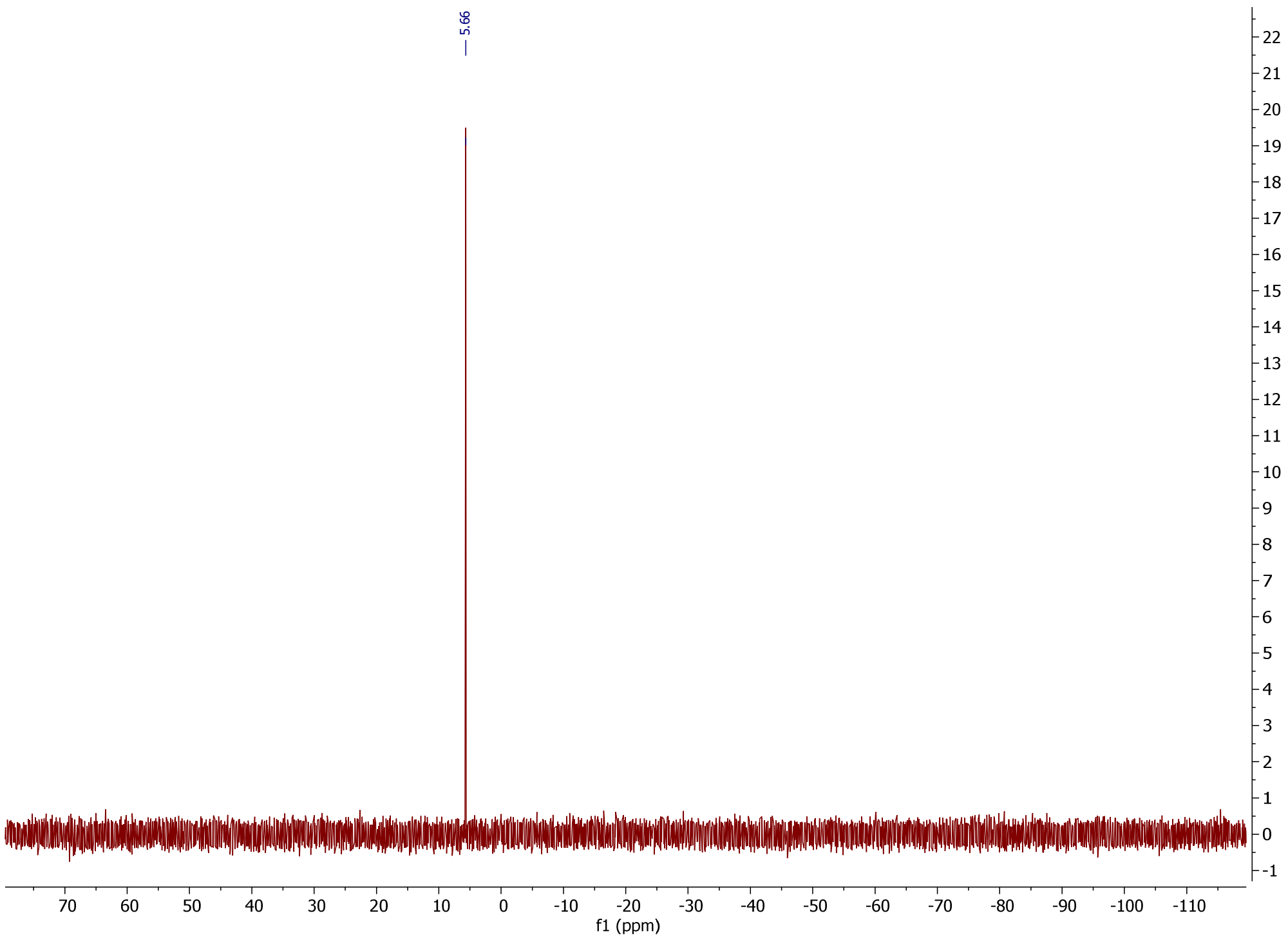

Figure S14c: ^{29}Si-NMR-Spectrum of ***3t6*** (100 MHz, pyridine-d$_5$).

References

S1 A. Immirzi, B. Perini, "Prediction of Density in Organic Crystals," *Acta Crystallographica Section A* 33 (1977): 216-218.

S2 C. Anders, M. Wagner, S. Poppe, C. Tschierske, "2D and 3D Nets by Self-Assembly of Carbosilane Functionalized Polyphilic Liquid Crystals," (2026): submitted to ZAAC.

S3 A. Lehmann, A. Scholte, M. Prehm, F. Liu, X. Zeng, G Ungar, C. Tschierske, "Soft Rectangular Sub-5 nm Tiling Patterns by Liquid Crystalline Self-Assembly of T-Shaped Bolapolyphiles", *Adv. Funct. Mater.* (2018): 1804162.

S4 H. G. O. Becker, R. Beckert, *Organikum*, 23. Auflage, Wiley VHC, Weinheim, 2009.

S5 R. L.-Y. Bao, R. Zhao, L. Shi, „Progress and developments in the turbo Grignard reagent i-PrMgCl·LiCl: a ten-year journey," *Chem. Commun.* 51 (2015): 6884-6900.

S6 B. T. Nguyen, "*Hydrosilation with Platinum Free Neat Copper Containing Catalyst."* US Patent 6713644 B1, March 30, 2004.

S7 B. N. Dolgov, Y. I. Khudobin, N. P. Kharitonov, "Reaction of trialkylsilanes with phenol in the presence of metal halides," *Izv. Akad. Nauk SSSR, Ser. Khim.* (1959): 1238–1243.

S8 Y.-C. Chiang, H.-C. Wu, H.-F. Wen, C.-C. Hung, C.-W. Hong, C.-C. Kuo, T. Higashihara, W. C. Chen, „Tailoring Carbosilane Side Chains toward Intrinsically Stretchable Semiconducting Polymers," *Macromolecules* 52 (2019): 4396-4404.

S9 A. Williamson, "Theory of etherification." *Philos. Mag.* 3 (1850): 350-356.

S10 R. van Rijsbergen, M. J. O. Anteunis, A. De Bruyn, „Selective removal of the isopropylidene group in 4-O-protected 1, 6-anhydro-2, 3-O-isopropylidene-β-d-mannopyranose and the conformational impact of it," *J. Carbohydr. Chem.* 2 (1983): 395-404.
